# Transcriptome-Wide Analysis of Messenger RNA Decay in Normal and Osteoarthritic Human Articular Chondrocytes

**DOI:** 10.1002/art.38849

**Published:** 2014-10-26

**Authors:** Simon R Tew, Benjamin T McDermott, Rory B Fentem, Mandy J Peffers, Peter D Clegg

**Affiliations:** 1 University of Liverpool, Leahurst Campus, NestonCheshire, UK

## Abstract

**Objective:**

Messenger RNA (mRNA) decay rates control not only gene expression levels, but also responsiveness to altered transcriptional input. We undertook this study to examine transcriptome-wide posttranscriptional regulation in both normal and osteoarthritic (OA) human articular chondrocytes.

**Methods:**

Human articular chondrocytes were isolated from normal or OA tissue. Equine articular chondrocytes were isolated from young or old horses at a commercial abattoir. RNA decay was measured across the transcriptome in human cells by microarray analysis following an actinomycin D chase. Messenger RNA levels in samples were confirmed using quantitative reverse transcription–polymerase chain reaction.

**Results:**

Examination of total mRNA expression levels demonstrated significant differences in the expression of transcripts between normal and OA chondrocytes. Interestingly, almost no difference was observed in total mRNA expression between chondrocytes from intact OA cartilage and those from fibrillated OA cartilage. Decay analysis revealed a set of rapidly turned over transcripts associated with transcriptional control and programmed cell death that were common to all chondrocytes and contained binding sites for abundant cartilage microRNAs. Many transcripts exhibited altered mRNA half-lives in human OA chondrocytes compared to normal cells. Specific transcripts whose decay rates were altered were generally less stable in these pathologic cells. Examination of selected genes in chondrocytes from young and old healthy horses did not identify any change in mRNA turnover.

**Conclusion:**

This is the first investigation into the “posttranscriptome” of the chondrocyte. It identifies a set of short-lived chondrocyte mRNAs likely to be highly responsive to altered transcriptional input as well as mRNAs whose decay rates are affected in OA chondrocytes.

Regulation of the rate of messenger RNA (mRNA) decay performs a role in the control of steady-state gene expression levels and the kinetics of gene regulation (1). Impaired mRNA decay can lead to accumulation of transcript with a consequent effect on levels of translated protein (2). The rate of mRNA turnover will also influence the speed at which relative changes in mRNA expression and protein translation occur in response to an alteration in transcriptional input (3). Short-lived mRNAs will be induced and turned off more rapidly than will more stable transcripts when transcription increases (4). The processes that regulate the rate of mRNA decay are complex and involve the interaction of regulatory sequences within the mRNAs themselves with proteins and noncoding RNAs such as microRNAs (miRNAs). There is great scope for such regulation to be further modulated, as the finite availability of these regulators is affected by changes in the network of protein-coding mRNAs and nontranslated pseudogene mRNAs (5,6).

A number of studies have examined transcriptome-wide control of gene expression in chondrocytes from healthy and arthritic cartilage and in chondrocytes exposed to various physiologically relevant conditions (7–14). These valuable studies continue to inform us about the mechanisms controlling chondrocyte gene expression and the cellular processes that are underpinned by these changes. By their nature, these current studies examine a snapshot of the levels of genes present in cells, and we are unable to infer from them how mRNA turnover is affected.

Currently, examination of chondrocyte mRNA turnover has been targeted to the regulation of specific genes. Chondrocytic cells have been shown to regulate genes such as inducible nitric oxide synthase and bone morphogenetic protein 2 posttranscriptionally (15,16). Our own work has shown that posttranscriptional control of the transcription factor SOX9 can be regulated through stress signaling pathways (17,18). These ongoing studies have also shown that hyperosmotic stress is able to induce changes in the rate of turnover of a number of other genes' mRNA, indicating that chondrocytes, like many other cells, use widespread regulation of mRNA turnover as a regulatory mechanism (19). Further indirect evidence of active posttranscriptional gene control in chondrocytes comes from studies of miRNA expression in chondrocytes. These small noncoding RNAs are able to control mRNA decay and protein translation by binding to seed sequences within target mRNAs (20). It is now clear that these molecules are critical for proper chondrocyte function (21) and are involved in diverse cellular processes including cell signaling (22,23), mechanical forces (24), and tissue pathology (22,25).

Analysis of mRNA decay using transcriptomic approaches has been applied to a number of cell types, and advances have been made in understanding how mRNA kinetics are affected by cytokine signaling and during cellular processes such as stem cell differentiation (26–28). In this study, we examined the transcriptome-wide rates of mRNA decay in human chondrocytes obtained from both osteoarthritic (OA) and normal tissue. This allowed us to examine for the first time the patterns of mRNA turnover in these cells and to begin to examine how this process is affected by pathology.

## MATERIALS AND METHODS

### Tissue collection and chondrocyte culture

Osteochondral tissue was taken from femoral condyles obtained from the knee joints of patients undergoing either limb resections as a treatment for osteosarcoma (Royal National Orthopaedic Hospital, Stanmore, Middlesex, UK) or total knee arthroplasty as a result of OA (Clatterbridge Hospital, Wirral, UK). Equine cartilage was obtained from the metacarpophalangeal joints of horses at a commercial abattoir. The age of the animals was determined from their legal documentation. Tissue was obtained with full ethical review board approval at each center. Cartilage was cut from the underlying subchondral bone, diced finely, and then digested by shaking overnight in 0.08% type II collagenase (Worthington) at 37°C. Isolated chondrocytes were plated at 100,000 cells/cm^2^ in 25-cm^2^ tissue culture flasks. The basal medium used throughout this study was Dulbecco's modified Eagle's medium (DMEM) containing 10% fetal bovine serum, 100 units/ml penicillin, 100 units/ml streptomycin, and 5 μg/ml amphotericin B (all medium constituents from Life Technologies).

### Grading of tissue pathology

From each tissue sample an osteochondral fragment grossly representative of the articular surface used for cartilage harvest was fixed in 4% formalin, dehydrated, decalcified, and processed into paraffin wax blocks. Sections (5 μm) were stained with hematoxylin and eosin or with Safranin O and were then graded by 2 blinded observers (BTM, PDC) using a modified Mankin grading system (29). Samples were categorized as normal if they were obtained from non-OA joints and had a score of <10, as having intact OA if they were from an OA joint and had a score of <10, and as having fibrillated OA if they were from an OA joint and had a score of ≥10 (Table1).

**Table 1:** Sample information and results of histologic grading

Category, age/sex*	Average histologic score	Sample reference
Intact normal		
18/M	3	N_M18†‡
15/M	0	N_M15†‡
14/M	1	N_M14‡
24/F	1	N_F24‡
21/M	3	N_M21†‡
37/F	2	N_F37†
16/F	1	N_F16†‡
Intact OA		
63/M	6	I_M63†‡
80/M	8	I_M80†‡
68/F	5	I_F68†‡
78/F	9	I_F78†‡
72/F	4	I_F72‡
66/M	4	I_M66‡
Fibrillated OA		
73/M	15	F_M73†
61/M	12	F_M61†
74/M	21	F_M74†
60/M	17	F_M60†

* OA = osteoarthritis.

† Used in microarray analysis.

‡ Used in real-time reverse transcription–polymerase chain reaction analysis.

### Screening of chondrocyte RNA decay using microarrays

Freshly isolated chondrocytes were allowed to adhere for 16 hours, and then their media were supplemented with 1 μ*M* actinomycin D (Sigma) for 0, 1, 3, or 5 hours. At each time point, the cell layer was extracted using TRIzol, and total RNA was purified using chloroform-based phase separation and subsequent precipitation with isopropanol. RNA pellets were dissolved in RNase-free H_2_O and stored at −80°C until required. Total RNA from the 0-, 1-, 3-, and 5-hour actinomycin D time points from 4 samples from each group were used to probe Illumina HT-12 BeadChip arrays at The Genome Centre core facility at Queen Mary University of London. RNA integrity was confirmed using an Agilent bioanalyzer, and RNA was then labeled with Cy3 using an Ambion TotalPrep kit before being hybridized to arrays using an Illumina BeadStation 500G. Non-normalized data were exported from Illumina BeadStudio software and used for subsequent analysis.

### Data analysis of RNA decay sample sets

Non-normalized expression data were normalized using R 3.0.2 for Mac OS X (http://cran.r-project.org/bin/macosx/) using a BeadArray package. For analysis of total mRNA levels, samples obtained from each donor at time 0 were quantile-normalized together before further data analysis. This data set is referred to as “total.” For mRNA decay measurements, probes that did not show significant hybridization above background at time 0 were excluded, as measurement of further decay of mRNA would not be possible. This was determined using the *P* value assigned to each hybridization. From the remaining data, the 4 sample points of each donor's RNA decay curve (0, 1, 3, and 5 hours) were taken and quantile-normalized to each other, independently of the other donors' sample points, to ensure that overall variations of gene expression between samples did not influence the rates of decay intrinsic to each sample (the “decay” data set). Data were submitted to ArrayExpress (http://www.ebi.ac.uk/arrayexpress/) for curation under accession no. E-MTAB-2723.

The “total” data were imported into the online National Institute on Aging Array Analysis tool (http://lgsun.grc.nia.nih.gov/ANOVA/index.html), where they were stratified by disease state (normal, intact OA, and fibrillated OA) and subjected to one-way analysis of variance. Significant differences in gene expression for the “total” data set were determined, and a 2-fold change with a *P* value less than 0.05 was considered significant.

The “decay” data set was analyzed in R. The slope of the decay curves was calculated using the following equation:

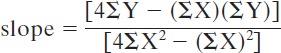
where Y is the natural logarithm of the expression level and X is the time of incubation with actinomycin D in hours. Each slope value was then converted into an mRNA half-life using the following equation:

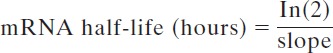


Longer half-life values were capped at 24 hours, and the data were filtered to include only genes whose half-life was a positive value in all samples. Mean values of mRNA half-lives in normal, intact OA, and fibrillated OA samples were calculated for each gene. Data for intact OA and fibrillated OA samples were also pooled to create a mean OA half-life. Ratios of half-lives from cells from different tissues were calculated, and those with >2-fold change were subjected to a 2-sample *t*-test. *P* values were adjusted to false discovery rates (FDRs), and those with values of less than 0.05 were then further screened by manual inspection of the raw data.

### Gene ontology analysis

Analysis of the rapidly turned over and differentially regulated mRNAs was performed using both the Panther Classification System web tool (www.pantherdb.org) and DAVID functional annotation clustering (30). Predicted miRNA targets of rapidly turned over chondrocyte mRNAs were identified using the Geneset2miRNA function within the www.BioProfiling.de web portal (31).

### MicroRNA microarray analysis

Cartilage was dissected from the femoral surface of osteochondral tissue obtained following total knee arthroplasty. Tissue from 2 female donors (ages 83 and 88 years) was cut into ∼5-mm^2^ pieces and cultured in serum-free DMEM (adjusted to 380 mOsm/liter by adding NaCl) for 24 hours. Tissue was then snap-frozen and powdered using a Braun Mikro-Dismembrator. RNA was isolated from these samples and used, then partially purified using TRIzol/chloroform before full purification using RNeasy Spin columns (Qiagen). Total RNA (1 μg) was sent to The Genome Centre and used to probe an Illumina Universal 12 v2 1536 BeadArray as previously described (19). Data from isolated chondrocytes from this earlier study were also reexamined.

### Real-time reverse transcription–polymerase chain reaction (RT-PCR) analysis

Total RNA was reverse-transcribed using Moloney murine leukemia virus reverse transcriptase, primed using random primers (Promega). Real-time RT-PCR was performed on an ABI 7300 system using MESA Blue SYBR Green reagent (Eurogentec). Expression levels were calculated by the 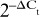 method (32). RPS13 was used as a reference expression gene due to its low variation in our microarray data set and its separate validation as being low in variance across a substantial number of other studies (33). Primer sequences are available on request from the corresponding author.

## RESULTS

Femoral cartilage was obtained from 7 non-OA and 10 OA human knee joints. Table1 shows the results of histologic grading of the tissue. The 7 non-OA tissue samples all had low scores when assessed histologically by the modified Mankin grading. The OA samples were split into 2 groups according to disease severity, as described in Materials and Methods: 6 intact samples with scores <10 and 4 fibrillated samples with scores ≥10.

**Figure 1:**
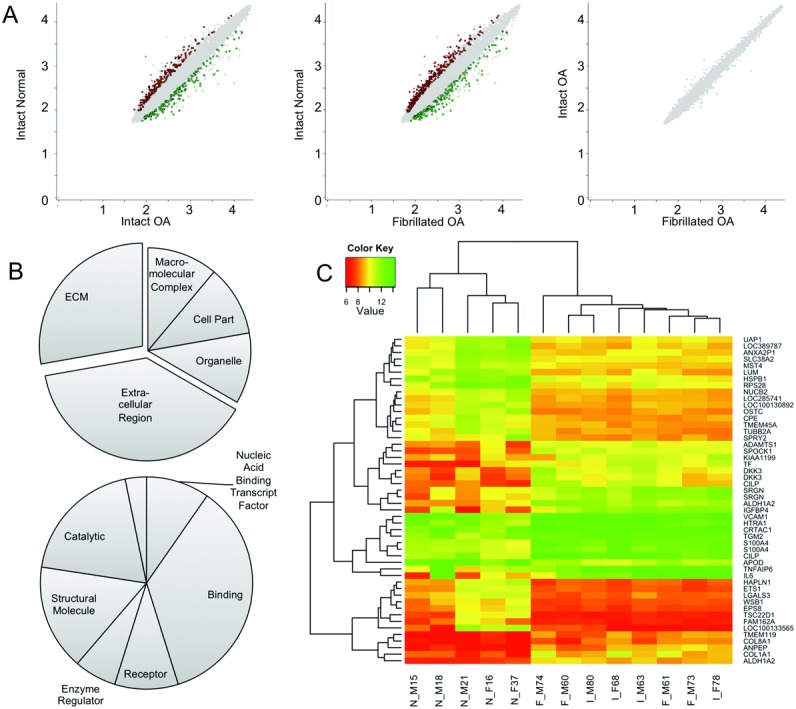
A, Scatterplots demonstrating the relationships between log_2_ gene expression levels between normal chondrocytes, intact osteoarthritic (OA) chondrocytes, and fibrillated OA chondrocytes (green and red dots represent genes that exhibit change between groups with *P* < 0.01). B, Pie charts representing the occurrence of major ontological annotations associated with genes significantly up-regulated and down-regulated in OA chondrocytes, either in location (top) or in process (bottom) categories of the Panther Classification System. Exploded regions on charts emphasize the number of extracellular-associated genes differentially regulated. C, Heatmap representing expression levels of the top 25 up-regulated and the top 25 down-regulated genes in OA chondrocytes. Both the gene and sample lists were subjected to hierarchical clustering. Color key indicates the normalized log_2_ hybridization values. Average histologic scores of samples at the bottom are shown in Table1. ECM = extracellular matrix.

Total RNA from each of the actinomycin D time points, taken from 5 normal samples and 4 samples from each OA group (indicated in Table1), was used to probe Illumina HT-12 microarrays. We first decided to examine the overall mRNA levels in each group, which was possible by simply examining the time 0 point of the decay curve for each sample. Our analysis was able to show that there were a number of significantly differentially regulated genes detectable between normal samples, intact OA samples, and fibrillated OA samples (Figure 1A). Gene ontology analysis using Panther (Figure 1B) showed enrichment of genes associated with the extracellular compartment, particularly the extracellular matrix (ECM), and genes involved in biologic processes such as binding, transcriptional regulation, and catalysis. In many cases, the genes identified had previously been associated with cartilage and OA (Figure 1C) (see Supplemental Table 1, available on the *Arthritis & Rheumatology* web site at http://onlinelibrary.wiley.com/doi/10.1002/art.38849/abstract). This gave us confidence that our cells, despite a short period of in vitro culture, retained intrinsic disease-dependent differences. The genes differentially regulated by OA were often similar regardless of whether the chondrocytes were from fibrillated or intact cartilage. Interestingly, when we compared overall mRNA expression levels between intact OA samples and fibrillated OA samples, we observed no statistically significant difference in expression (Figure 1A).

**Figure 2:**
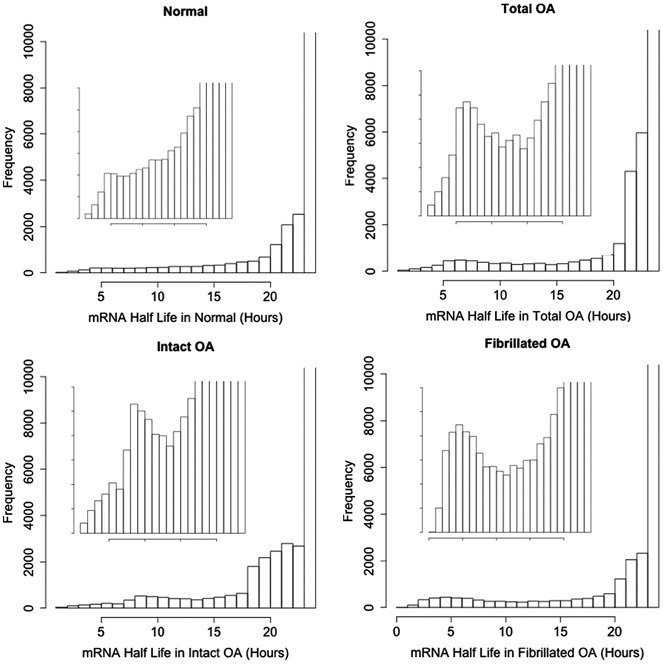
Profile of mRNA half-life in normal and osteoarthritic (OA) chondrocytes and in OA chondrocytes stratified into those isolated from intact OA and fibrillated OA tissue. The analysis caps longer half-lives at 24 hours, which accounts for the high peak at the far right in each panel (which extends beyond the limit of the y-axis in each case). Inserts show the same data on zoomed y-axis.

We next examined mRNA half-life in articular chondrocytes. Figure 2 shows the frequency of mRNA half-lives in each sample group. The data showed that the majority of mRNAs in chondrocytes were extremely stable. However, a substantial number of mRNAs in each sample set exhibited rapid turnover. We observed that the number of short-lived transcripts was higher in chondrocytes from OA tissue than in chondrocytes from normal tissue. This was the case when samples were stratified by intact OA or fibrillated OA and when all OA samples were combined together (total OA). We next examined transcripts that had a half-life of <6 hours across all samples, to identify mRNAs that were turned over quickly by chondrocytes regardless of pathology. We identified 213 genes that consistently exhibited an mRNA half-life of <6 hours (see Supplemental Table 2, available on the *Arthritis & Rheumatology* web site at http://onlinelibrary.wiley.com/doi/10.1002/art.38849/abstract), representing 0.6% of genes whose mRNA half-life was calculated. Using DAVID to perform functional annotation clustering, we found that many of these short-lived transcripts encoded genes localized to the nucleus and responsible for transcription (functional annotation score 8.5), genes involved in programmed cell death (functional annotation score 5.65), and genes that regulate embryonic development (functional annotation score 3.27).

As mRNA decay is determined in part through the actions of miRNAs, we were interested to know whether the rapidly turned over mRNAs were enriched for targets of particular miRNA molecules. Using the Geneset2miRNA function within the www.BioProfiling.de web portal, we found that the short-lived mRNAs were enriched for predicted targets of the miRNAs miR-410, miR-323.3p, miR-200b, miR-200c, and miR-429 (see Supplemental Table 2, available on the *Arthritis & Rheumatology* web site at http://onlinelibrary.wiley.com/doi/10.1002/art.38849/abstract). We took these miRNA predictions and compared them with microarray-based miRNA expression measurements that we previously conducted on isolated human chondrocytes and explanted human OA cartilage. We found that of the 1,145 miRNA molecules examined by the microarray, <10% hybridized to the array at sufficient levels to provide a signal of >10,000 light units. The miRNA miR-323.3p was among these highly expressed miRNAs. Furthermore, miR-410, miR-200b, and miR-200c were in the top 50% of miRNAs expressed in the chondrocytes and cartilage explants.

We next directly compared the mRNA half-lives in chondrocytes in normal and OA cartilage. We focused on transcripts whose half-life changed by a factor of 2. We used *t*-tests and subsequent FDR correction to determine the number of mRNAs whose half-life was significantly altered in the groups examined. We found that 51 mRNAs were identified when comparing normal and intact OA samples and 112 when comparing normal and fibrillated OA samples but none when comparing intact OA and fibrillated OA samples. Because there was no significant difference between intact OA and fibrillated OA samples, we chose to pool these data sets to maximize the power of the comparison to the normal samples. This allowed us to identify 395 mRNAs whose half-life was significantly altered in OA chondrocytes compared to normal chondrocytes (see Supplemental Table 3, available on the *Arthritis & Rheumatology* web site at http://onlinelibrary.wiley.com/doi/10.1002/art.38849/abstract).

**Figure 3:**
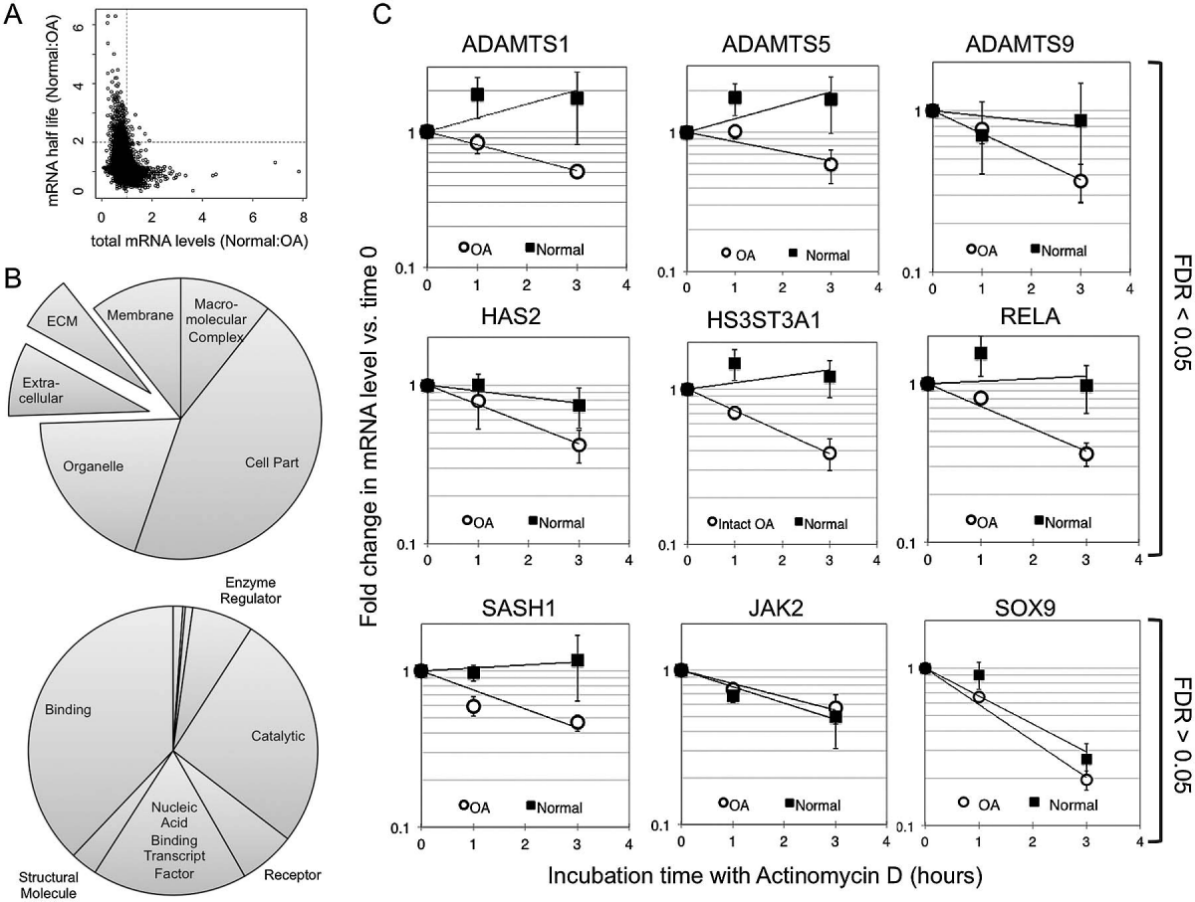
Effect of osteoarthritis (OA) on mRNA decay. A, Scatterplot relating the observed fold change in total mRNA levels between normal and OA cells to the fold change in mRNA half-life. The vertical line represents a normal:OA total mRNA ratio of 1:1, such that all points to the left of the line represent genes with increased expression in OA. The horizontal line represents a 2-fold change in the normal:OA mRNA half-life ratio, such that genes that are above this line are less stable in OA cells. B, Pie charts representing the occurrence of major ontological annotations associated with genes significantly up-regulated and down-regulated in OA chondrocytes, either in location (top) or in process (bottom) categories of the Panther Classification System. Exploded regions on charts emphasize the number of extracellular-associated genes differentially regulated. C, Real-time reverse transcription–polymerase chain reaction analysis of mRNA decay, confirming alterations between normal and intact OA chondrocytes in mRNA decay of selected genes. Values are mean ± SEM fold change in mRNA levels compared to 0 hours of the decay curve (data from 5 normal donors and 5 OA donors; see Table1). Data are presented for 6 genes in which the false discovery rate (FDR) was <0.05 in microarray analysis and for 3 genes in which the average half-life was altered but the FDR was >0.05. ECM = extracellular matrix.

Consistent with the distributions shown in Figure 3, the majority of significantly affected mRNAs became less stable in OA chondrocytes. Only 8 mRNAs were more stable, representing just 2% of those identified. We examined the relationship between the normal:OA ratio of mRNA half-life and steady-state mRNA expression, and we found that mRNAs whose average half-life was reduced in OA chondrocytes were generally expressed at higher levels (Figure 3A). We performed gene ontology analysis of the genes differentially regulated between these 2 groups using Panther. Compared to the list of genes whose steady state was altered by OA (Figure 1B), there was a lesser enrichment of genes associated with extracellular space and ECM location (Figure 3B), although the biologic properties were similarly varied. Further analysis of this gene list using DAVID indicated that mRNAs that were differentially regulated between each normal and OA sample were mainly associated with 4 annotation clusters: genes associated with transcriptional regulatory properties (functional annotation score 5.74), genes associated with the nuclear lumen (functional annotation score 3.29), genes that encode proteins containing a zinc finger domain (functional annotation score 3.22), and genes associated with regulation of programmed cell death (functional annotation score 1.9).

Quantitative RT-PCR confirmation of mRNA decay rates of differentially regulated genes of interest confirmed the microarray findings (Figure 3C). The aggrecanases ADAMTS1, ADAMTS5, and ADAMTS9, the hyaluronic acid synthase HAS2, the heparan sulfate sulfotransferase HS3ST3A1, and the NF-κB complex component RELA were examined by real-time RT-PCR. We also analyzed 3 genes that had demonstrated >2-fold change in half-life but that had FDR values of >0.05. JAK2 was chosen as it is a critical component of the JAK/STAT cytokine signaling pathway. SOX9 and SASH1 were chosen because we have previously shown their altered posttranscriptional regulation in chondrocytes. JAK2 (FDR 0.07) and SOX9 (FDR 0.51) did not exhibit a change in half-life; however, the quantitative RT-PCR data suggested that SASH1 (FDR 0.12) was differentially regulated.

**Figure 4:**
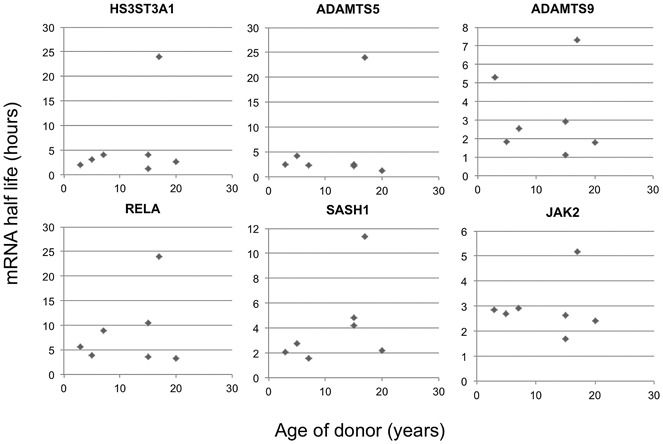
Effect of age on mRNA decay of candidate genes in equine articular chondrocytes. Plots show mRNA half-life measured in hours from freshly isolated cells, plotted against the age in years of the donor animal. Data are from 7 animals ranging in age from 3 years to 20 years. Symbols represent individual samples.

Due to the different ages of our normal human donors and our human donors with OA, we wished to examine how age affected the mRNA half-life of genes that we had identified in the study. To do this, we turned to an equine chondrocyte system, isolating the cells from animals whose age could be accurately identified at the time of death. We used the actinomycin D chase approach used with the human samples to create RNA decay curves, and from these we calculated RNA half-lives (Figure 4). We measured turnover of HS3ST3A1, ADAMTS5, ADAMTS9, RELA, and SASH1, all of which had differed in the quantitative RT-PCR analysis of normal and OA human samples. We also measured JAK2, which we had confirmed not to be differentially regulated in the human experiments. Samples were collected from horses ranging in age from 3 years (equivalent to a human age of ∼18 years) to 20 years (equivalent to a human age of ∼60 years) (34). We observed 1 outlying donor (age 17 years) that consistently exhibited higher half-lives for each RNA examined. Otherwise, we found no significant relationship between age and mRNA half-life for any of the genes examined.

## DISCUSSION

This study is the first to look in detail at the “posttranscriptome” of the human articular chondrocyte. Our findings show that chondrocytes, regardless of pathology, exhibit extensive control over the turnover of a selection of expressed transcripts. Furthermore, we present evidence that OA leads to altered posttranscriptional regulation of a number of genes. Previous studies of chondrocyte gene expression have captured a snapshot of gene expression under the specifically examined conditions. Our results begin to cast some light onto an additional dimension of gene expression within chondrocytes, due to the relationship between mRNA half-life and gene expression kinetics. We can begin to infer that genes whose mRNA is turned over rapidly are likely to be more responsive to transcriptional input than genes with pools of stable mRNA. This may have profound implications for our understanding of how chondrocytes regulate genes in response to changes in their environment.

Although our aim in this study was to examine RNA decay, we were able to use our data to first determine how OA affects steady-state mRNA levels, using the zero time point of our decay curves. There were major changes in genes associated with enzymatic activity and the ECM. Particular examples of these OA-related genes included genes encoding proteinases such as ADAMTS1, ADAMTS5, and HTRA1 (35–37). We also identified ECM macromolecules such as COL1A1, HAPLN1, and CILP and soluble regulatory molecules such as IL6, WNT5A, IGFBP4, APOD, and DKK3, which have previously been implicated in OA progression (38–43). This demonstrated that a different transcriptional profile, consistent with OA, was maintained following isolation of the cells from their ECM.

Interestingly, we found that the transcriptomes of chondrocytes from late-stage OA joints are remarkably similar, regardless of whether tissue was harvested from relatively intact or severely degenerated regions of the articular surface. This similarity between OA chondrocytes in different locations has been found previously by examining the transcriptional level of a number of candidate genes (44). It has also been shown that intact and degenerated OA cartilage are comparable in their stem cell content (45). However, other work has shown that gene expression changes can be found in cartilage at different stages of degeneration (13,14). In our experiments, it is possible that any difference in total mRNA expression that exists in OA cells from more or less severely affected tissue is lost during the 24-hour culture period but that changes between normal and OA cells persist.

Our preparation of a data set describing mRNA decay in chondrocytes has not previously been attempted. In general, in each cell type examined, the mRNAs were mostly very stable with their calculated half-life cut off by the analysis at 24 hours. Nevertheless, there were a number of rapidly turned over mRNAs in chondrocytes, and we were interested to find that there appeared to be an increase in the number of these rapidly turned over mRNAs in OA chondrocytes (Figure 2). Although this is a small change, it is consistent with findings in embryonic stem cells, which when allowed to differentiate, display relatively modest changes in mRNA decay rate profile (27). By examining a core set of mRNAs that are turned over rapidly (half-life <6 hours) in chondrocytes regardless of pathologic origin, we identified a group of uniformly short-lived transcripts. The gene ontologies associated with these short-lived mRNAs provides us with an increased appreciation of the processes that rely on rapid mRNA turnover. Given that we know that mRNA turnover rates will affect gene responsiveness, we can infer that it is important that the regulation of these processes is quick and flexible in chondrocytes. The primary groups of genes identified are those involved in transcriptional regulation as well as those localized to the nucleus. Furthermore, the ontology analysis suggests that quick mRNA turnover is associated with genes involved in the regulation of programmed cell death, which itself can be a rapid process in chondrocytes (46).

The rapid turnover of a large subset of mRNAs in chondrocytes will be controlled by regulatory factors with specificity for these transcripts. Numerous studies have already demonstrated a critical role of miRNAs in the shaping of gene expression posttranscriptionally, and key cartilage-related miRNAs continue to be discovered (47). The data presented here illustrate the consequences of this regulation in chondrocytes in terms of RNA turnover. This has allowed us to predict the types of miRNA that strongly influence the turnover of RNA in chondrocytes. We have done this by using bioinformatics tools to identify miRNAs whose seed sequences were enriched in our list of commonly unstable mRNAs. However, these lists contain many RNAs that do not contain these seed sequences, which suggests that other important factors exist, for instance, the influence of multiple lower affinity miRNAs or RNA binding proteins.

Despite the very similar profiles of mRNA half-lives in all the chondrocyte populations examined, we were excited to identify a number of transcripts whose decay rates were altered specifically in OA chondrocytes. These transcripts were largely less stable in OA chondrocytes but were also more highly expressed (Figure 3A). This indicates that altered transcription rates of these genes in OA are higher than steady-state levels alone would suggest. In addition, through this altered posttranscriptional regulation, OA may still substantially affect the regulatory dynamics controlling the expression of these genes, altering the speed of their response to further transcriptional stimulus (3). Further experiments examining the longitudinal expression kinetics of these identified genes in chondrocytes are clearly needed.

Our candidate gene confirmatory analysis also demonstrated differential regulation in a variety of genes, including RELA, a key transcription factor component of the NF-κB pathway, the transcription factor SASH1, which we have previously shown is controlled by hyperosmotic stimulation of chondrocytes (19), and HS3ST3A1, which encodes a heparan sulfate sulfotransferase enzyme. We were interested to find that the HAS2 mRNA was identified as being posttranscriptionally regulated in OA. It encodes the enzyme hyaluronic acid synthase 2, which performs a critical role in cartilage matrix assembly (48). It has been shown that HAS2 mRNA can be regulated posttranscriptionally in fibroblasts following glucocorticoid treatment (49) and in osteosarcoma cells by the natural antisense RNA HAS2-AS1 (50). We were also particularly interested to find that the ADAMTS1, ADAMTS5, and ADAMTS9 mRNAs, which encode 3 aggrecanase enzymes (36,51,52), were turned over differently in OA chondrocytes. Aggrecanases are responsible for the breakdown of the proteoglycan aggrecan in the cartilage ECM, which is crucial to healthy turnover of cartilage matrix but can contribute to the development of OA. ADAMTS5 has been identified as being critical to OA progression in mice (35), and posttranscriptional regulation of ADAMTS5 has already been implicated in the development of OA through the action of miR-140 (53). To our knowledge, this is the first observation of a change in aggrecanase mRNA decay kinetics in OA samples.

This is the first time that mRNA decay has been investigated across the transcriptome in chondrocytes. There are limitations in our current study that should be identified. We have been using cells that are isolated from tissue to ensure that the transcriptional inhibitor actinomycin D would have uniform, rapid access to them. Therefore, the cells will no longer experience environmental cues from the cartilage ECM. This may mean that a process of homogenization, due to the culture process, may mask subtler differences in cells originating from different tissues. We are currently developing ways of conducting these experiments in explant culture to avoid these issues. A further limitation is that the human chondrocyte experiments use normal tissue samples, which were obtained from donors who were considerably younger than the donors with OA. As a consequence, the differences that we have seen between cells from these samples could be described as being affected by aging and/or pathology. Therefore, to determine the influence of age on mRNA decay in chondrocytes, we analyzed young and old equine chondrocytes, and we found in a set of a candidate genes that aging does not influence mRNA turnover. This supports the notion that pathology is the driver of altered mRNA decay in our human study, although a future study examining aging in human tissue is warranted to confirm this.

In conclusion, using an in vitro human chondrocyte culture system, we have been able to shed light on the posttranscriptional regulatory landscape that exists within chondrocytes. This has allowed us to identify common patterns of mRNA turnover as well as specific differences that occur due to pathology. As our awareness of these processes develops, it should lead to improvements in our understanding of gene and protein regulation within cartilage during health and disease.
