# Supplementary material for: Transcriptome-Wide Analysis of Messenger RNA Decay in Normal and Osteoarthritic Human Articular Chondrocytes
Source: Arthritis Rheumatol. 2014 Oct 26;66(11):3052–61. doi: 10.1002/art.38849 (PMC4233952; doi:10.1002/art.38849)
Supplement: Supplementary file 1 [file art0066-3052-sd1.pdf]

Tew et al: Transcriptome-wide analysis of mRNA decay in normal and osteoarthritic human articular chondrocytes

Supplemental Data 1

This file contains four tab of data (in addition to this tab) which each have lists of genes that show significant (FDR < 0.05) up or down regulation between normal chondrocytes and chondrocytes from either intact or fibrillated tissue
The comparison is indicated by the header to the right.
Explanation of column names:
Illumina ID
Mean(Normal) = log10 mean expression level
Mean(Intact/Fibrillated) = log 10 mean expression level
LogRatio
FoldChng = unlogged fold change in expression
FDR = False Discovery Rate value
Symbol = Official Gene Symbol

Illumina ID	Mean(Normal)	Mean(Intact)	LogRatio	FoldChng	FDR	Symbol
ILMN_1699651	2.611	3.995	-1.384	24.22	1.50E-03	IL6
ILMN_1665865	2.590	3.640	-1.050	11.21	8.30E-03	IGFBP4
ILMN_1768425	2.436	3.287	-0.851	7.10	1.56E-02	TF
ILMN_1763837	1.980	2.748	-0.768	5.86	0.00E+00	ANPEP
ILMN_1785732	3.364	4.120	-0.756	5.70	4.72E-02	TNFAIP6
ILMN_2169152	2.832	3.568	-0.736	5.44	2.80E-03	SRGN
ILMN_1760347	2.888	3.621	-0.733	5.40	4.00E-04	SRGN
ILMN_1748538	2.805	3.535	-0.730	5.37	0.00E+00	ALDH1A2
ILMN_1738116	1.898	2.613	-0.715	5.19	1.00E-04	TMEM119
ILMN_2167758	2.440	3.135	-0.695	4.96	7.10E-03	CILP
ILMN_1746013	2.551	3.224	-0.673	4.71	1.00E-04	SPOCK1
ILMN_1723847	3.401	4.055	-0.654	4.51	5.00E-04	CILP
ILMN_1701308	2.307	2.957	-0.650	4.46	3.57E-02	COL1A1
ILMN_2398159	2.597	3.224	-0.628	4.24	4.50E-03	DKK3
ILMN_1685433	1.953	2.575	-0.622	4.18	3.30E-03	COL8A1
ILMN_2383707	2.221	2.841	-0.620	4.17	0.00E+00	ALDH1A2
ILMN_1800317	1.814	2.421	-0.607	4.05	2.80E-02	WNT5A
ILMN_1815673	2.519	3.099	-0.580	3.80	3.39E-02	DKK3
ILMN_1688780	3.339	3.899	-0.560	3.63	0.00E+00	S100A4
ILMN_1662619	2.155	2.687	-0.532	3.40	4.51E-02	TFPI
ILMN_1813704	2.788	3.312	-0.525	3.35	2.21E-02	KIAA1199
ILMN_1813341	2.068	2.585	-0.517	3.29	1.00E-04	PTGFR
ILMN_1671142	2.250	2.763	-0.513	3.26	1.40E-03	GPR68
ILMN_1673566	2.669	3.175	-0.506	3.20	1.80E-02	ADAMTS1
ILMN_1684306	3.325	3.823	-0.497	3.14	0.00E+00	S100A4
ILMN_1734611	2.136	2.625	-0.489	3.09	3.84E-02	BDKRB1
ILMN_1656057	2.145	2.631	-0.486	3.06	0.00E+00	PLAU
ILMN_1736078	2.631	3.110	-0.479	3.01	3.57E-02	THBS4
ILMN_1660031	2.258	2.729	-0.471	2.96	3.62E-02	P2RY6
ILMN_1676563	3.642	4.109	-0.467	2.93	2.14E-02	HTRA1
ILMN_1675797	2.328	2.791	-0.463	2.91	0.00E+00	EPDR1
ILMN_1792455	2.681	3.128	-0.446	2.80	1.60E-02	TMEM158
ILMN_2325763	2.622	3.067	-0.445	2.79	2.29E-02	VCAM1
ILMN_2402392	1.943	2.375	-0.433	2.71	2.20E-02	COL8A1
ILMN_1670926	2.469	2.900	-0.430	2.69	3.80E-03	CHST15
ILMN_1658384	3.749	4.176	-0.427	2.67	1.40E-03	CRTAC1
ILMN_1782938	2.322	2.745	-0.423	2.65	3.00E-04	SLC16A10
ILMN_1713499	2.449	2.872	-0.422	2.64	2.22E-02	WISP1
ILMN_1910180	2.729	3.148	-0.420	2.63	7.00E-04	
ILMN_1740015	2.460	2.878	-0.418	2.62	9.50E-03	CD14
ILMN_2395652	2.073	2.475	-0.402	2.52	1.00E-04	PTGFR
ILMN_1802780	1.822	2.216	-0.394	2.48	8.00E-04	M160
ILMN_2307903	3.781	4.171	-0.389	2.45	1.29E-02	VCAM1
ILMN_1656938	2.341	2.730	-0.389	2.45	4.80E-02	LOC731486
ILMN_3245564	1.860	2.248	-0.389	2.45	0.00E+00	RICH2
ILMN_1681515	2.425	2.811	-0.386	2.43	5.90E-03	CRLF1
ILMN_1732921	2.018	2.404	-0.386	2.43	4.00E-04	ITGB8
ILMN_1705750	3.612	3.997	-0.385	2.43	1.55E-02	TGM2
ILMN_1691892	2.419	2.804	-0.385	2.43	0.00E+00	TAGLN2
ILMN_1761858	1.974	2.359	-0.385	2.43	2.20E-03	MID1
ILMN_2090105	3.229	3.612	-0.383	2.41	6.00E-04	TAGLN2
ILMN_2396444	3.073	3.454	-0.381	2.40	2.21E-02	CD14
ILMN_1696048	2.671	3.050	-0.379	2.39	2.77E-02	C13orf33
ILMN_2385672	1.987	2.363	-0.376	2.38	1.10E-02	ELN
ILMN_1801710	2.015	2.390	-0.376	2.38	1.01E-02	APBB1IP
ILMN_1800787	2.139	2.512	-0.373	2.36	3.28E-02	RFTN1
ILMN_1760778	2.998	3.365	-0.367	2.33	8.30E-03	ENG

ILMN_1702973	1.896	2.259	-0.363	2.31	4.30E-03	TMEM166
ILMN_1727671	2.251	2.608	-0.356	2.27	6.60E-03	SSH1
ILMN_1717934	2.245	2.598	-0.353	2.26	0.00E+00	SYT11
ILMN_1758672	2.551	2.901	-0.350	2.24	1.90E-03	FAM107B
ILMN_1701643	2.223	2.567	-0.345	2.21	4.14E-02	GDPD5
ILMN_1746465	2.399	2.743	-0.345	2.21	2.51E-02	FJX1
ILMN_1680132	2.007	2.349	-0.342	2.20	1.00E-04	CADM1
ILMN_1690939	2.097	2.430	-0.333	2.15	4.79E-02	ECGF1
ILMN_1815745	2.523	2.851	-0.328	2.13	3.13E-02	SOX4
ILMN_2394250	2.662	2.988	-0.326	2.12	1.40E-03	PLEKHA1
ILMN_1773389	2.225	2.549	-0.324	2.11	2.84E-02	PLTP
ILMN_1780334	1.995	2.314	-0.319	2.08	2.20E-03	KCNJ2
ILMN_1779228	1.784	2.102	-0.319	2.08	2.80E-03	CDH2
ILMN_1912737	2.183	2.497	-0.314	2.06	4.30E-03	
ILMN_1766539	2.325	2.636	-0.311	2.05	1.30E-03	LOC643319
ILMN_1873621	2.802	3.111	-0.310	2.04	1.04E-02	NTN1
ILMN_2062468	3.217	3.527	-0.310	2.04	8.60E-03	IGFBP7
ILMN_2383305	2.797	3.104	-0.307	2.03	5.40E-03	GPATCH4
ILMN_2309156	2.463	2.769	-0.307	2.03	1.70E-03	PMEPA1
ILMN_1655884	2.712	3.018	-0.306	2.03	4.90E-03	ATP2A2
ILMN_1714700	2.381	2.686	-0.306	2.02	1.30E-03	TRIB2
ILMN_1811468	2.019	2.317	-0.298	1.99	5.90E-03	IRX3
ILMN_1741054	2.663	2.960	-0.297	1.98	1.64E-02	SLC5A6
ILMN_1851610	1.883	2.175	-0.292	1.96	0.00E+00	
ILMN_1677432	2.191	2.483	-0.292	1.96	2.40E-02	SRGAP1
ILMN_1656300	2.170	2.462	-0.292	1.96	2.52E-02	GFRA2
ILMN_1789510	2.951	3.242	-0.291	1.95	5.80E-03	STIP1
ILMN_1681886	2.204	2.494	-0.291	1.95	3.00E-03	ADAMTS5
ILMN_1787265	2.854	3.144	-0.290	1.95	1.23E-02	ZNF503
ILMN_1669631	2.083	2.372	-0.289	1.95	1.60E-03	GLRB
ILMN_2065022	1.901	2.190	-0.289	1.95	4.00E-04	KIAA0672
ILMN_1687440	2.369	2.657	-0.288	1.94	8.70E-03	HIPK2
ILMN_2229877	1.907	2.194	-0.288	1.94	1.16E-02	PCDH18
ILMN_2110252	2.764	3.051	-0.287	1.94	3.05E-02	NPM3
ILMN_1712684	3.358	3.644	-0.286	1.93	8.30E-03	FAM20C
ILMN_2366041	3.029	3.315	-0.286	1.93	2.76E-02	ITM2C
ILMN_1774823	2.888	3.171	-0.284	1.92	1.54E-02	RPL34
ILMN_1665736	3.175	3.456	-0.282	1.91	1.39E-02	LOC648024
ILMN_3244611	2.274	2.554	-0.280	1.90	1.56E-02	LOC100132060
ILMN_1678692	2.382	2.659	-0.277	1.89	1.10E-02	MPRIP
ILMN_1683271	3.771	4.046	-0.275	1.88	1.60E-03	TMSB4X
ILMN_1656628	2.815	3.089	-0.274	1.88	1.97E-02	WDR4
ILMN_1692077	2.116	2.388	-0.272	1.87	6.40E-03	MXRA7
ILMN_1798172	2.552	2.821	-0.269	1.86	1.16E-02	IPO4
ILMN_1674183	2.077	2.344	-0.267	1.85	4.34E-02	MT1JP
ILMN_1717490	2.606	2.873	-0.267	1.85	3.96E-02	RPL6
ILMN_1767253	2.448	2.714	-0.266	1.85	1.25E-02	RRP12
ILMN_1848913	2.037	2.302	-0.265	1.84	1.29E-02	
ILMN_1794392	3.117	3.381	-0.264	1.84	2.03E-02	DDX3X
ILMN_1771051	2.392	2.655	-0.262	1.83	3.62E-02	RPL29
ILMN_1676014	2.380	2.642	-0.262	1.83	1.25E-02	LOC728635
ILMN_1654262	2.583	2.844	-0.261	1.82	2.75E-02	ZMAT3
ILMN_3237632	2.828	3.087	-0.259	1.82	3.04E-02	NCLN
ILMN_1811104	2.946	3.205	-0.259	1.82	2.69E-02	KTELC1
ILMN_1757867	2.140	2.398	-0.258	1.81	1.20E-02	MTM
ILMN_2129927	2.311	2.567	-0.256	1.80	2.69E-02	EXT1
ILMN_2385220	2.506	2.761	-0.255	1.80	1.98E-02	DFFA
ILMN_1696485	2.559	2.814	-0.255	1.80	2.49E-02	HNRNPAB

ILMN_1781999	2.337	2.592	-0.255	1.80	1.59E-02	ABCF2
ILMN_1669362	3.474	3.729	-0.255	1.80	1.47E-02	IGFBP6
ILMN_1741200	2.454	2.707	-0.254	1.79	2.40E-02	RFX5
ILMN_1710740	2.058	2.312	-0.254	1.79	1.25E-02	C2
ILMN_1809352	1.840	2.093	-0.253	1.79	0.00E+00	PRL
ILMN_1785252	2.473	2.725	-0.252	1.79	4.11E-02	SLC26A6
ILMN_1730794	2.119	2.370	-0.251	1.78	4.40E-02	SERTAD4
ILMN_3242540	1.847	2.097	-0.250	1.78	1.00E-04	CD163L1
ILMN_1756784	2.205	2.455	-0.250	1.78	1.77E-02	FREQ
ILMN_1858599	2.209	2.458	-0.249	1.77	1.98E-02	
ILMN_1812926	2.490	2.738	-0.248	1.77	1.48E-02	ANTXR2
ILMN_1705908	2.871	3.118	-0.247	1.77	4.56E-02	RPL7L1
ILMN_2315789	2.408	2.654	-0.246	1.76	2.60E-02	PTPRD
ILMN_1795930	2.185	2.430	-0.246	1.76	3.93E-02	PTGER4
ILMN_1774547	2.934	3.179	-0.245	1.76	4.75E-02	MPRIP
ILMN_1795218	2.716	2.960	-0.244	1.75	3.57E-02	DHX30
ILMN_1799814	2.520	2.764	-0.244	1.75	3.05E-02	WDR57
ILMN_1727815	2.107	2.350	-0.243	1.75	1.47E-02	CFI
ILMN_1809364	1.916	2.159	-0.243	1.75	2.70E-03	NTF3
ILMN_1704305	2.778	3.019	-0.241	1.74	4.72E-02	NIP7
ILMN_1744308	2.391	2.630	-0.239	1.73	2.69E-02	DHX33
ILMN_1701933	1.975	2.214	-0.239	1.73	7.40E-03	SNCA
ILMN_1735499	2.467	2.706	-0.239	1.73	2.98E-02	DCBLD2
ILMN_2189222	2.429	2.667	-0.239	1.73	4.68E-02	KLHL8
ILMN_1710885	3.798	4.034	-0.236	1.72	1.30E-02	LOC387867
ILMN_1695485	2.053	2.289	-0.236	1.72	2.20E-02	C9orf109
ILMN_1728024	2.323	2.557	-0.234	1.71	2.79E-02	TUBG1
ILMN_1703487	3.066	3.299	-0.232	1.71	4.68E-02	LMO4
ILMN_3206343	3.374	3.606	-0.232	1.71	4.09E-02	LOC644914
ILMN_1738657	1.891	2.123	-0.231	1.70	2.20E-03	SATB2
ILMN_1813379	1.904	2.132	-0.228	1.69	1.03E-02	TNFRSF9
ILMN_1701243	2.334	2.562	-0.227	1.69	4.14E-02	C10orf2
ILMN_2411745	2.493	2.720	-0.227	1.69	4.17E-02	EML1
ILMN_1784113	2.126	2.350	-0.224	1.67	3.04E-02	NAT14
ILMN_1665148	3.631	3.852	-0.222	1.67	4.35E-02	LOC648249
ILMN_1801307	1.878	2.098	-0.220	1.66	2.20E-03	TNFSF10
ILMN_1685834	1.978	2.197	-0.219	1.65	4.94E-02	AMPH
ILMN_1672608	1.989	2.206	-0.217	1.65	3.28E-02	PPAP2B
ILMN_1728218	1.893	2.109	-0.216	1.65	2.80E-03	RBPMS
ILMN_1808590	1.987	2.202	-0.214	1.64	1.59E-02	GUCY1A3
ILMN_1782275	1.936	2.146	-0.210	1.62	2.92E-02	P2RY6
ILMN_1745599	1.906	2.112	-0.206	1.61	6.40E-03	LOC643977
ILMN_3240900	1.851	2.056	-0.206	1.61	1.30E-03	LOC100134728
ILMN_1675756	2.005	2.210	-0.205	1.60	3.93E-02	KCNJ15
ILMN_1762095	1.882	2.085	-0.203	1.60	6.70E-03	TMTC4
ILMN_1797362	1.957	2.159	-0.203	1.59	3.57E-02	LIMK1
ILMN_2233783	1.908	2.106	-0.197	1.58	2.69E-02	CD38
ILMN_1666775	1.906	2.096	-0.190	1.55	7.10E-03	CACNA1C
ILMN_1797764	1.935	2.115	-0.180	1.51	2.98E-02	RPL22L1
ILMN_2206474	1.796	1.975	-0.179	1.51	1.64E-02	TMEM90B
ILMN_1717799	1.899	2.077	-0.178	1.51	2.83E-02	PRKCE
ILMN_1687213	1.861	2.038	-0.178	1.51	2.99E-02	C8orf13
ILMN_1677723	1.925	2.102	-0.177	1.50	2.53E-02	ANGPT1
ILMN_1756928	1.805	1.980	-0.176	1.50	6.00E-04	RTN1
ILMN_1657039	1.869	2.037	-0.168	1.47	6.50E-03	FILIP1
ILMN_1747347	1.922	2.088	-0.166	1.47	4.09E-02	C17orf60
ILMN_1663519	1.852	2.018	-0.167	1.47	2.99E-02	SLC24A3
ILMN_1788955	1.819	1.980	-0.161	1.45	2.69E-02	PDLIM1

ILMN_2313378	1.866	2.019	-0.153	1.42	1.74E-02	SPAG6
ILMN_2413779	1.753	1.904	-0.151	1.42	1.56E-02	SEZ6L2
ILMN_1804339	1.833	1.979	-0.146	1.40	1.49E-02	CAMK1G
ILMN_2322996	1.845	1.990	-0.145	1.40	1.87E-02	EYA2
ILMN_1813528	1.858	1.997	-0.139	1.38	2.68E-02	CCBE1
ILMN_3279611	1.815	1.954	-0.138	1.37	4.05E-02	LOC100131960
ILMN_1692477	1.855	1.989	-0.134	1.36	2.37E-02	MGAT5B
ILMN_1689086	1.868	1.998	-0.129	1.35	3.76E-02	CTSC
ILMN_1861059	1.829	1.955	-0.126	1.34	2.81E-02	
ILMN_1791511	1.847	1.966	-0.120	1.32	3.57E-02	TMEM176A

Illumina ID	Mean(Normal)	Mean(Intact)	LogRatio	FoldChng	FDR	Symbol
ILMN_3239610	3.217	1.981	1.237	17.25	1.00E-04	LOC100133565
ILMN_2412380	2.801	2.098	0.703	5.05	2.16E-02	TSC22D1
ILMN_2210519	3.064	2.474	0.590	3.89	1.04E-02	HAPLN1
ILMN_1780170	3.891	3.308	0.582	3.82	0.00E+00	APOD
ILMN_2167805	3.435	2.861	0.574	3.75	1.30E-03	LUM
ILMN_2122103	2.993	2.434	0.559	3.62	1.10E-02	ETS1
ILMN_1803647	2.672	2.129	0.542	3.48	3.57E-02	FAM162A
ILMN_1651699	2.881	2.352	0.529	3.38	8.30E-03	EPS8
ILMN_2056167	3.190	2.664	0.526	3.36	1.17E-02	OSTC
ILMN_1803788	3.084	2.561	0.524	3.34	8.00E-04	LGALS3
ILMN_1665823	3.365	2.847	0.518	3.30	4.33E-02	LOC389787
ILMN_2089329	3.174	2.663	0.511	3.24	1.16E-02	SPRY2
ILMN_2353161	2.410	1.907	0.503	3.19	3.30E-03	MSLN
ILMN_1731374	3.221	2.725	0.496	3.13	0.00E+00	CPE
ILMN_1651228	3.607	3.118	0.489	3.08	1.98E-02	RPS28
ILMN_2041101	3.449	2.972	0.477	3.00	1.35E-02	ANXA2P1
ILMN_1768582	2.531	2.056	0.475	2.99	3.28E-02	PPP2CB
ILMN_2038775	3.196	2.729	0.467	2.93	5.50E-03	TUBB2A
ILMN_1664802	2.882	2.415	0.467	2.93	2.20E-03	WSB1
ILMN_1655913	3.257	2.793	0.464	2.91	1.00E-04	NUCB2
ILMN_2221006	2.647	2.184	0.463	2.90	1.39E-02	RAD21
ILMN_1675448	2.880	2.422	0.458	2.87	2.69E-02	ZFP36L1
ILMN_1674236	3.577	3.119	0.458	2.87	1.70E-03	HSPB1
ILMN_2045419	2.512	2.058	0.454	2.85	4.30E-03	BNIP3L
ILMN_2148913	3.186	2.738	0.449	2.81	2.20E-03	TMEM45A
ILMN_1704760	2.688	2.242	0.446	2.79	4.58E-02	BZW1
ILMN_1694810	2.506	2.068	0.438	2.74	1.00E-04	PANX2
ILMN_1656386	2.754	2.318	0.436	2.73	5.80E-03	SEC24D
ILMN_3216336	3.168	2.736	0.432	2.70	2.27E-02	LOC285741
ILMN_1674620	2.914	2.488	0.426	2.67	1.16E-02	SGCE
ILMN_3197767	2.851	2.426	0.425	2.66	3.54E-02	LOC645691
ILMN_1742461	3.375	2.951	0.424	2.66	2.40E-02	UAP1
ILMN_3195253	3.202	2.782	0.420	2.63	1.95E-02	LOC100130892
ILMN_3203444	2.898	2.482	0.416	2.61	0.00E+00	LOC100132535
ILMN_2371590	3.025	2.618	0.407	2.56	3.79E-02	DDX17
ILMN_1676159	3.294	2.890	0.403	2.53	1.00E-04	MST4
ILMN_1775814	2.743	2.341	0.402	2.53	0.00E+00	GHR
ILMN_1808824	3.022	2.624	0.398	2.50	2.65E-02	NEBL
ILMN_2181540	2.663	2.264	0.399	2.50	1.98E-02	YY1
ILMN_2328776	3.034	2.636	0.398	2.50	2.00E-04	MST4
ILMN_1658015	2.462	2.065	0.397	2.50	4.21E-02	MBNL2
ILMN_1746664	2.680	2.284	0.396	2.49	4.21E-02	WSB2
ILMN_2202940	2.820	2.425	0.395	2.48	1.80E-02	CHPT1
ILMN_1792997	3.282	2.888	0.394	2.48	6.00E-04	NPTN
ILMN_1714709	2.921	2.528	0.394	2.48	0.00E+00	OLFM1
ILMN_2219712	2.545	2.153	0.391	2.46	2.69E-02	HMGB2
ILMN_2364272	2.845	2.460	0.386	2.43	6.40E-03	MBNL2
ILMN_2220283	2.886	2.504	0.382	2.41	3.76E-02	HNRPA1L-2
ILMN_1651799	3.411	3.029	0.382	2.41	5.00E-04	SLC38A2
ILMN_1757702	2.704	2.326	0.378	2.39	3.05E-02	LOC647673
ILMN_1802205	2.808	2.432	0.375	2.37	1.95E-02	RHOB
ILMN_2322806	2.567	2.192	0.375	2.37	7.00E-04	CAST
ILMN_1698100	2.643	2.268	0.374	2.37	1.90E-02	ANXA2P1
ILMN_1689400	2.590	2.222	0.367	2.33	3.57E-02	CLK1
ILMN_1764090	2.912	2.549	0.363	2.31	1.30E-03	AK3L1
ILMN_2358457	3.031	2.673	0.358	2.28	3.39E-02	ATF4
ILMN_2196347	2.439	2.086	0.353	2.26	2.21E-02	CDKN1B

ILMN_3231881	2.601	2.251	0.350	2.24	3.92E-02	LOC728026
ILMN_1692177	3.241	2.892	0.350	2.24	9.00E-03	TSC22D1
ILMN_1666894	3.504	3.155	0.349	2.23	2.63E-02	CSPG4
ILMN_2228732	2.349	2.000	0.349	2.23	1.50E-03	CCNG2
ILMN_2327994	2.220	1.872	0.349	2.23	3.93E-02	AZIN1
ILMN_1722532	2.461	2.115	0.346	2.22	7.00E-04	JMJD1A
ILMN_3241234	3.409	3.065	0.344	2.21	5.30E-03	LOC730278
ILMN_1700896	2.879	2.536	0.343	2.20	3.05E-02	SAP30
ILMN_1670439	2.757	2.414	0.343	2.20	2.89E-02	FYTTD1
ILMN_1687080	3.616	3.274	0.342	2.20	1.98E-02	LOC402251
ILMN_1679045	2.530	2.190	0.340	2.19	2.84E-02	SBDS
ILMN_2079786	2.664	2.326	0.337	2.18	6.40E-03	NUAK1
ILMN_1693136	2.702	2.365	0.337	2.17	1.74E-02	VTI1B
ILMN_2398107	2.904	2.570	0.334	2.16	1.58E-02	ASNS
ILMN_1788778	2.737	2.406	0.331	2.14	4.97E-02	11-Sep
ILMN_1690894	2.389	2.060	0.328	2.13	1.69E-02	TRA1P2
ILMN_1663313	3.516	3.190	0.327	2.12	1.98E-02	AMY1C
ILMN_1765019	2.622	2.297	0.325	2.11	3.87E-02	SACM1L
ILMN_2117508	2.301	1.977	0.325	2.11	1.88E-02	CTHRC1
ILMN_2342841	2.449	2.125	0.324	2.11	1.13E-02	AFTPH
ILMN_1673421	2.839	2.518	0.321	2.09	3.43E-02	LOC440704
ILMN_1711408	2.228	1.908	0.321	2.09	3.70E-02	ANXA4
ILMN_1709479	2.983	2.662	0.321	2.09	1.90E-02	YAP1
ILMN_2150465	3.135	2.814	0.321	2.09	1.39E-02	C5orf28
ILMN_3235803	2.306	1.988	0.319	2.08	2.51E-02	LOC653458
ILMN_1668861	2.506	2.188	0.318	2.08	2.47E-02	LOC732165
ILMN_1742025	3.220	2.904	0.317	2.07	3.70E-03	OLFM1
ILMN_1713752	2.302	1.987	0.315	2.07	4.33E-02	SERINC3
ILMN_1685140	2.879	2.569	0.309	2.04	6.00E-04	PRRC1
ILMN_1693334	3.322	3.013	0.309	2.04	3.70E-03	P4HA1
ILMN_1723123	3.286	2.978	0.308	2.03	4.36E-02	FGFR3
ILMN_1727738	2.162	1.856	0.306	2.02	2.20E-03	RAB33B
ILMN_1734696	2.909	2.604	0.306	2.02	1.58E-02	FRG1
ILMN_2186482	2.983	2.678	0.305	2.02	2.03E-02	TMED7
ILMN_2373266	2.589	2.284	0.305	2.02	1.53E-02	SFRS12
ILMN_2153916	2.478	2.175	0.304	2.01	4.56E-02	HSPA2
ILMN_3302499	2.630	2.330	0.300	2.00	4.40E-02	LOC730990
ILMN_2383913	2.457	2.159	0.298	1.99	1.55E-02	WDR33
ILMN_1669788	2.629	2.331	0.298	1.98	2.89E-02	NUDT14
ILMN_1692948	2.353	2.056	0.297	1.98	3.90E-02	CCDC90B
ILMN_1787567	3.721	3.426	0.295	1.97	3.15E-02	TSC22D1
ILMN_1745110	3.014	2.721	0.293	1.96	2.47E-02	LAPTM4A
ILMN_2331163	2.589	2.297	0.292	1.96	1.24E-02	CUL4A
ILMN_3279712	2.873	2.581	0.291	1.96	2.20E-03	LOC642590
ILMN_3239548	2.414	2.124	0.290	1.95	2.84E-02	LOC100132740
ILMN_1747744	3.149	2.860	0.289	1.95	3.04E-02	LHFPL2
ILMN_1681467	2.226	1.937	0.289	1.94	4.60E-03	RAB11FIP4
ILMN_2102960	2.280	1.992	0.288	1.94	4.79E-02	KIAA1370
ILMN_1829845	2.787	2.500	0.287	1.94	4.03E-02	
ILMN_1694426	2.765	2.478	0.287	1.94	6.50E-03	ROR2
ILMN_1770053	2.877	2.590	0.287	1.93	4.79E-02	RBBP7
ILMN_1721758	2.006	1.720	0.286	1.93	4.33E-02	ID4
ILMN_2154322	3.182	2.897	0.285	1.93	3.57E-02	SEMA3E
ILMN_1785765	3.269	2.985	0.284	1.92	1.55E-02	TM9SF2
ILMN_2153332	2.343	2.059	0.284	1.92	4.18E-02	ATXN1
ILMN_1664449	2.968	2.685	0.282	1.92	8.20E-03	ALG5
ILMN_2371055	2.983	2.704	0.279	1.90	3.75E-02	EFNA1
ILMN_1803819	3.125	2.846	0.279	1.90	1.36E-02	IQGAP1

ILMN_2147105	2.828	2.550	0.279	1.90	6.40E-03	LOC440348
ILMN_3187357	2.218	1.939	0.279	1.90	4.39E-02	LOC100130746
ILMN_1725090	2.876	2.597	0.279	1.90	8.90E-03	CTHRC1
ILMN_2046611	2.517	2.240	0.277	1.89	4.76E-02	MCOLN3
ILMN_2294976	2.734	2.458	0.276	1.89	2.52E-02	RNASE4
ILMN_3240117	3.104	2.831	0.273	1.87	1.60E-02	AIDA
ILMN_1733176	3.099	2.826	0.273	1.87	1.60E-02	LIMS1
ILMN_3250345	2.332	2.062	0.270	1.86	3.89E-02	LOC283788
ILMN_1693421	3.017	2.748	0.270	1.86	1.16E-02	RPN2
ILMN_1725387	2.302	2.033	0.270	1.86	2.80E-03	TMEM200A
ILMN_2063586	3.759	3.489	0.269	1.86	1.16E-02	CLIC4
ILMN_2091375	2.483	2.214	0.270	1.86	1.39E-02	KRCC1
ILMN_2362581	2.553	2.286	0.267	1.85	2.69E-02	FNDC3A
ILMN_1764396	2.224	1.958	0.266	1.85	4.33E-02	HDAC4
ILMN_1800078	2.170	1.904	0.265	1.84	3.57E-02	LMO2
ILMN_3310416	2.325	2.061	0.264	1.84	2.70E-03	SNORD114-3
ILMN_1805800	2.622	2.358	0.263	1.83	4.16E-02	RAB5A
ILMN_2136971	2.098	1.835	0.262	1.83	3.95E-02	FABP3
ILMN_3291472	2.374	2.113	0.262	1.83	3.63E-02	LOC442727
ILMN_2151441	2.434	2.173	0.261	1.82	8.70E-03	FAM103A1
ILMN_2356955	2.215	1.956	0.259	1.81	1.59E-02	PLAGL1
ILMN_2107613	2.309	2.051	0.257	1.81	1.39E-02	RHOJ
ILMN_1778617	2.277	2.020	0.257	1.81	3.05E-02	TAF9
ILMN_2145997	2.297	2.040	0.257	1.81	5.10E-03	SP4
ILMN_1775962	2.434	2.178	0.255	1.80	2.21E-02	MCOLN3
ILMN_1665945	3.014	2.759	0.254	1.80	2.63E-02	ACBD3
ILMN_2338921	2.568	2.314	0.254	1.79	3.93E-02	C4orf41
ILMN_1703622	2.199	1.946	0.253	1.79	1.50E-03	PPIB
ILMN_2182704	3.356	3.105	0.251	1.78	2.64E-02	BIRC2
ILMN_1761131	3.034	2.784	0.250	1.78	2.69E-02	PECI
ILMN_2062381	2.335	2.085	0.249	1.78	8.50E-03	LCOR
ILMN_1738095	2.701	2.452	0.249	1.78	1.65E-02	PER2
ILMN_1751227	2.468	2.219	0.249	1.77	1.65E-02	LOC401321
ILMN_1707337	2.337	2.091	0.246	1.76	2.52E-02	MSTO1
ILMN_2126832	2.308	2.062	0.246	1.76	3.57E-02	SEC24A
ILMN_1661888	2.520	2.274	0.246	1.76	4.09E-02	MEF2A
ILMN_3307659	2.529	2.284	0.245	1.76	2.22E-02	SFT2D2
ILMN_1738407	2.237	1.995	0.242	1.74	4.72E-02	ULBP1
ILMN_2353358	2.453	2.213	0.240	1.74	3.66E-02	LGALS8
ILMN_2213558	3.776	3.538	0.238	1.73	3.03E-02	TMED10P
ILMN_2408645	2.882	2.645	0.237	1.73	4.21E-02	LOC653566
ILMN_2190414	2.543	2.307	0.236	1.72	3.06E-02	ZNF83
ILMN_1696975	2.669	2.434	0.235	1.72	3.04E-02	USP1
ILMN_1726693	2.296	2.061	0.234	1.72	1.64E-02	GTF2H1
ILMN_2230566	2.631	2.398	0.234	1.71	3.46E-02	RAB40B
ILMN_1806790	2.255	2.022	0.233	1.71	1.49E-02	ROBO1
ILMN_1772998	2.368	2.135	0.233	1.71	2.52E-02	LOC647436
ILMN_3303965	2.887	2.654	0.232	1.71	4.99E-02	ZC3H11B
ILMN_1794187	2.357	2.125	0.232	1.71	2.87E-02	FBXL3
ILMN_1796537	2.098	1.866	0.232	1.71	3.30E-03	FYB
ILMN_1689976	2.786	2.555	0.231	1.70	4.20E-02	EDIL3
ILMN_1661917	4.099	3.870	0.229	1.69	1.49E-02	LOC644039
ILMN_1663149	2.244	2.016	0.227	1.69	3.15E-02	SRP54
ILMN_3298829	2.165	1.938	0.227	1.69	3.41E-02	LOC729505
ILMN_2212690	2.106	1.879	0.227	1.69	1.79E-02	ZC3H7A
ILMN_3251436	2.118	1.893	0.225	1.68	1.27E-02	DENND4C
ILMN_3282395	2.238	2.013	0.225	1.68	1.47E-02	LOC646966
ILMN_1656670	2.422	2.202	0.220	1.66	4.68E-02	HLA-G

ILMN_1683959	2.204	1.988	0.216	1.64	3.39E-02	MED13L
ILMN_1675106	2.210	1.995	0.215	1.64	1.47E-02	YIPF2
ILMN_2133784	2.386	2.173	0.213	1.63	4.79E-02	PATE2
ILMN_3242462	2.231	2.020	0.212	1.63	1.68E-02	UHRF1BP1
ILMN_1801068	2.200	1.989	0.212	1.63	3.93E-02	DACT1
ILMN_2398388	2.273	2.061	0.212	1.63	3.43E-02	APH1A
ILMN_2320906	2.186	1.975	0.211	1.63	2.16E-02	RTN3
ILMN_3201480	4.143	3.933	0.210	1.62	2.14E-02	LOC643358
ILMN_2204664	2.225	2.017	0.208	1.61	1.64E-02	NBPF14
ILMN_1702946	2.252	2.045	0.207	1.61	3.02E-02	THUMPD1
ILMN_2135339	2.279	2.072	0.207	1.61	3.62E-02	C3orf70
ILMN_1801923	2.226	2.026	0.199	1.58	4.11E-02	ATF1
ILMN_2223720	2.143	1.944	0.199	1.58	8.30E-03	ATMIN
ILMN_1739622	2.251	2.052	0.199	1.58	4.45E-02	PPP1R12A
ILMN_1682233	2.187	1.990	0.196	1.57	2.90E-02	ESCO1
ILMN_1781173	2.002	1.824	0.178	1.51	2.42E-02	HDAC9
ILMN_1723969	2.045	1.872	0.173	1.49	2.80E-03	PLCB1
ILMN_1776640	1.992	1.820	0.172	1.49	1.64E-02	MPL
ILMN_2148193	1.996	1.832	0.164	1.46	4.77E-02	MPPED2
ILMN_3236373	2.078	1.922	0.157	1.43	4.34E-02	MSL2
ILMN_1780496	2.068	1.920	0.148	1.41	4.34E-02	MGC12760
ILMN_3277321	2.018	1.875	0.142	1.39	4.79E-02	LOC392264
ILMN_1712211	1.998	1.861	0.137	1.37	3.72E-02	FLJ10246
ILMN_3294741	2.012	1.877	0.136	1.37	2.76E-02	LOC644496
ILMN_1779333	2.003	1.875	0.128	1.34	4.09E-02	MSRB3

Illumina ID	Mean(Normal)	Mean(Fibrillated	LogRatio	FoldChng	FDR	Symbol
ILMN_1699651	2.611	3.826	-1.215	16.39	8.40E-03	IL6
ILMN_1763837	1.980	2.804	-0.823	6.66	0.00E+00	ANPEP
ILMN_2167758	2.440	3.224	-0.784	6.08	1.10E-03	CILP
ILMN_1738116	1.898	2.635	-0.737	5.46	0.00E+00	TMEM119
ILMN_1792455	2.681	3.411	-0.730	5.37	0.00E+00	TMEM158
ILMN_1748538	2.805	3.515	-0.711	5.14	0.00E+00	ALDH1A2
ILMN_1746013	2.551	3.238	-0.687	4.86	0.00E+00	SPOCK1
ILMN_1723847	3.401	4.077	-0.676	4.75	2.00E-04	CILP
ILMN_1800317	1.814	2.488	-0.675	4.73	9.20E-03	WNT5A
ILMN_1701308	2.307	2.973	-0.666	4.63	2.55E-02	COL1A1
ILMN_1707124	2.196	2.838	-0.642	4.39	2.08E-02	TFPI
ILMN_2383707	2.221	2.849	-0.627	4.24	0.00E+00	ALDH1A2
ILMN_1760347	2.888	3.514	-0.625	4.22	4.70E-03	SRGN
ILMN_2169152	2.832	3.452	-0.620	4.17	1.83E-02	SRGN
ILMN_1662619	2.155	2.773	-0.618	4.15	1.09E-02	TFPI
ILMN_2398159	2.597	3.214	-0.617	4.14	4.70E-03	DKK3
ILMN_2212878	2.056	2.660	-0.604	4.02	2.08E-02	ESM1
ILMN_1815673	2.519	3.115	-0.596	3.94	2.39E-02	DKK3
ILMN_2387385	2.119	2.712	-0.593	3.91	2.60E-03	IGFBP1
ILMN_1739001	2.570	3.155	-0.585	3.84	6.00E-04	TACSTD2
ILMN_1688780	3.339	3.920	-0.581	3.81	0.00E+00	S100A4
ILMN_1751161	2.409	2.986	-0.577	3.78	3.15E-02	COL7A1
ILMN_1789418	2.069	2.638	-0.570	3.71	7.00E-04	GSTT2
ILMN_1684306	3.325	3.849	-0.524	3.34	0.00E+00	S100A4
ILMN_3240433	2.015	2.535	-0.520	3.31	0.00E+00	GSTT2B
ILMN_1671142	2.250	2.768	-0.518	3.29	1.10E-03	GPR68
ILMN_1685433	1.953	2.463	-0.510	3.23	2.55E-02	COL8A1
ILMN_1673566	2.669	3.152	-0.483	3.04	2.43E-02	ADAMTS1
ILMN_1702973	1.896	2.372	-0.476	2.99	0.00E+00	TMEM166
ILMN_1656057	2.145	2.618	-0.472	2.97	0.00E+00	PLAU
ILMN_1670926	2.469	2.924	-0.455	2.85	1.40E-03	CHST15
ILMN_2355549	1.987	2.440	-0.453	2.84	3.00E-04	GSTT2
ILMN_1675797	2.328	2.777	-0.450	2.82	0.00E+00	EPDR1
ILMN_1654396	2.280	2.717	-0.438	2.74	8.80E-03	ITGB2
ILMN_1717934	2.245	2.669	-0.425	2.66	0.00E+00	SYT11
ILMN_1769388	1.968	2.391	-0.423	2.65	0.00E+00	GJB2
ILMN_2062468	3.217	3.639	-0.422	2.64	0.00E+00	IGFBP7
ILMN_1800540	2.537	2.958	-0.421	2.64	7.00E-03	CD55
ILMN_1681515	2.425	2.839	-0.415	2.60	1.80E-03	CRLF1
ILMN_3245564	1.860	2.264	-0.405	2.54	0.00E+00	RICH2
ILMN_1910180	2.729	3.128	-0.399	2.51	1.40E-03	
ILMN_1760778	2.998	3.392	-0.393	2.47	2.80E-03	ENG
ILMN_1761858	1.974	2.367	-0.393	2.47	1.40E-03	MID1
ILMN_1800787	2.139	2.526	-0.388	2.44	2.07E-02	RFTN1
ILMN_1712112	2.734	3.121	-0.387	2.44	3.90E-03	RCAN1
ILMN_1696048	2.671	3.056	-0.385	2.43	2.08E-02	C13orf33
ILMN_1656938	2.341	2.722	-0.381	2.40	4.95E-02	LOC731486
ILMN_3244611	2.274	2.651	-0.377	2.38	1.00E-04	LOC100132060
ILMN_1692077	2.116	2.488	-0.373	2.36	0.00E+00	MXRA7
ILMN_1701204	2.232	2.604	-0.372	2.35	1.22E-02	VEGFC
ILMN_1652413	1.849	2.218	-0.369	2.34	3.68E-02	UCN2
ILMN_1655884	2.712	3.079	-0.367	2.33	2.00E-04	ATP2A2
ILMN_2175912	2.381	2.747	-0.367	2.33	2.62E-02	ITGB2
ILMN_1688703	2.010	2.372	-0.361	2.30	1.37E-02	C17orf47
ILMN_2396444	3.073	3.433	-0.360	2.29	3.14E-02	CD14
ILMN_1696347	2.434	2.793	-0.359	2.29	1.75E-02	CTSC
ILMN_1701643	2.223	2.580	-0.357	2.28	2.78E-02	GDPD5

ILMN_1705750	3.612	3.967	-0.355	2.26	2.78E-02	TGM2
ILMN_2134974	2.309	2.663	-0.354	2.26	9.00E-04	RAB38
ILMN_1707727	3.475	3.824	-0.349	2.23	3.14E-02	ANGPTL4
ILMN_1812926	2.490	2.836	-0.346	2.22	0.00E+00	ANTXR2
ILMN_1674183	2.077	2.419	-0.343	2.20	2.90E-03	MT1JP
ILMN_1785252	2.473	2.815	-0.342	2.20	1.10E-03	SLC26A6
ILMN_1813341	2.068	2.410	-0.342	2.20	3.43E-02	PTGFR
ILMN_1883255	2.038	2.380	-0.342	2.20	1.40E-02	LOC729254
ILMN_1658384	3.749	4.087	-0.337	2.17	2.13E-02	CRTAC1
ILMN_1687440	2.369	2.704	-0.335	2.16	8.00E-04	HIPK2
ILMN_2090105	3.229	3.564	-0.335	2.16	4.10E-03	TAGLN2
ILMN_2367239	3.362	3.695	-0.333	2.15	9.40E-03	RCAN1
ILMN_1764850	2.841	3.173	-0.332	2.15	1.95E-02	HPCAL1
ILMN_1802780	1.822	2.149	-0.327	2.12	1.04E-02	M160
ILMN_1691892	2.419	2.745	-0.326	2.12	3.00E-04	TAGLN2
ILMN_1678170	1.774	2.100	-0.326	2.12	4.91E-02	MME
ILMN_1811468	2.019	2.343	-0.324	2.11	1.50E-03	IRX3
ILMN_1656300	2.170	2.491	-0.322	2.10	8.80E-03	GFRA2
ILMN_1873621	2.802	3.119	-0.318	2.08	6.60E-03	NTN1
ILMN_1819384	2.262	2.577	-0.315	2.06	2.54E-02	
ILMN_1683271	3.771	4.084	-0.313	2.06	1.00E-04	TMSB4X
ILMN_1669362	3.474	3.787	-0.313	2.06	7.00E-04	IGFBP6
ILMN_2309156	2.463	2.774	-0.312	2.05	1.10E-03	PMEPA1
ILMN_2065022	1.901	2.211	-0.311	2.04	0.00E+00	KIAA0672
ILMN_1848913	2.037	2.345	-0.308	2.03	1.50E-03	
ILMN_1789510	2.951	3.257	-0.306	2.02	2.30E-03	STIP1
ILMN_1804789	2.449	2.753	-0.304	2.01	4.97E-02	KIAA1967
ILMN_3307906	2.534	2.836	-0.302	2.00	1.77E-02	PALMD
ILMN_1779228	1.784	2.086	-0.302	2.00	5.20E-03	CDH2
ILMN_1685397	2.586	2.887	-0.301	2.00	2.88E-02	ITGA3
ILMN_1781761	1.809	2.108	-0.299	1.99	5.00E-04	ENPP4
ILMN_1766539	2.325	2.622	-0.297	1.98	2.20E-03	LOC643319
ILMN_1912737	2.183	2.481	-0.297	1.98	7.40E-03	
ILMN_1727671	2.251	2.548	-0.296	1.98	3.41E-02	SSH1
ILMN_1671151	2.012	2.307	-0.296	1.98	1.70E-02	RARB
ILMN_1665736	3.175	3.468	-0.294	1.97	7.50E-03	LOC648024
ILMN_2394250	2.662	2.956	-0.293	1.97	5.50E-03	PLEKHA1
ILMN_2395652	2.073	2.366	-0.293	1.96	1.61E-02	PTGFR
ILMN_1794501	1.974	2.266	-0.293	1.96	1.83E-02	HAS3
ILMN_1743836	2.714	3.005	-0.291	1.95	6.40E-03	MXRA7
ILMN_1680132	2.007	2.293	-0.286	1.93	2.90E-03	CADM1
ILMN_2129927	2.311	2.595	-0.284	1.92	8.80E-03	EXT1
ILMN_1678692	2.382	2.666	-0.284	1.92	6.80E-03	MPRIP
ILMN_3194638	1.843	2.126	-0.283	1.92	3.35E-02	FAM176A
ILMN_1732921	2.018	2.300	-0.282	1.92	2.44E-02	ITGB8
ILMN_1717639	2.020	2.302	-0.282	1.91	1.81E-02	SIK1
ILMN_1758672	2.551	2.831	-0.280	1.91	2.25E-02	FAM107B
ILMN_1811104	2.946	3.225	-0.279	1.90	1.21E-02	KTELC1
ILMN_1851610	1.883	2.161	-0.278	1.90	2.00E-04	
ILMN_3304898	2.944	3.220	-0.276	1.89	1.56E-02	LOC92755
ILMN_3306742	2.735	3.011	-0.276	1.89	1.19E-02	SIGMAR1
ILMN_1688480	3.476	3.751	-0.275	1.88	1.63E-02	CCND1
ILMN_1781999	2.337	2.612	-0.275	1.88	6.10E-03	ABCF2
ILMN_1696485	2.559	2.834	-0.275	1.88	1.08E-02	HNRNPAB
ILMN_1787265	2.854	3.128	-0.274	1.88	1.76E-02	ZNF503
ILMN_1792885	2.212	2.475	-0.263	1.83	2.88E-02	CTSC
ILMN_1677432	2.191	2.453	-0.262	1.83	4.93E-02	SRGAP1
ILMN_2399893	2.952	3.212	-0.260	1.82	4.95E-02	RPS24

ILMN_3237632	2.828	3.087	-0.259	1.82	2.77E-02	NCLN
ILMN_1815666	2.901	3.160	-0.259	1.81	3.06E-02	ATP2A2
ILMN_1680618	2.958	3.211	-0.253	1.79	2.58E-02	MYC
ILMN_1695422	2.661	2.914	-0.253	1.79	3.46E-02	NCL
ILMN_2129505	2.642	2.895	-0.253	1.79	1.95E-02	CYBASC3
ILMN_1728024	2.323	2.574	-0.250	1.78	1.35E-02	TUBG1
ILMN_2374340	3.077	3.326	-0.249	1.77	4.29E-02	PLAUR
ILMN_1704305	2.778	3.026	-0.248	1.77	3.31E-02	NIP7
ILMN_1767253	2.448	2.695	-0.247	1.77	2.09E-02	RRP12
ILMN_2326282	2.634	2.877	-0.243	1.75	2.78E-02	C19orf6
ILMN_1721541	1.973	2.216	-0.243	1.75	4.29E-02	WIF1
ILMN_2334989	3.022	3.263	-0.241	1.74	3.61E-02	CCT3
ILMN_1756784	2.205	2.444	-0.239	1.73	2.29E-02	FREQ
ILMN_1809364	1.916	2.155	-0.239	1.73	3.00E-03	NTF3
ILMN_1651262	3.441	3.678	-0.237	1.73	3.34E-02	HNRNPAB
ILMN_1736441	2.635	2.870	-0.235	1.72	3.76E-02	PDXP
ILMN_1681886	2.204	2.438	-0.234	1.72	2.83E-02	ADAMTS5
ILMN_1672608	1.989	2.224	-0.234	1.72	1.48E-02	PPAP2B
ILMN_1814221	1.912	2.146	-0.234	1.71	4.06E-02	NPTX1
ILMN_2357809	2.281	2.514	-0.233	1.71	2.48E-02	G3BP1
ILMN_1747412	2.018	2.251	-0.233	1.71	1.45E-02	DPP3
ILMN_1690040	2.087	2.319	-0.232	1.71	2.28E-02	TM7SF2
ILMN_1693410	2.528	2.758	-0.230	1.70	2.59E-02	BRI3BP
ILMN_3206343	3.374	3.603	-0.230	1.70	3.83E-02	LOC644914
ILMN_1729115	2.532	2.762	-0.229	1.70	3.10E-02	LOC651816
ILMN_1735499	2.467	2.696	-0.229	1.70	3.59E-02	DCBLD2
ILMN_1798172	2.552	2.778	-0.226	1.68	4.43E-02	IPO4
ILMN_1669631	2.083	2.308	-0.226	1.68	2.61E-02	GLRB
ILMN_1712587	2.190	2.415	-0.225	1.68	3.06E-02	ARSB
ILMN_1696585	2.013	2.237	-0.224	1.67	1.37E-02	FERMT1
ILMN_1724609	2.595	2.819	-0.224	1.67	4.30E-02	SLC2A8
ILMN_1722397	1.860	2.083	-0.224	1.67	1.89E-02	LOC729137
ILMN_1695485	2.053	2.276	-0.223	1.67	3.10E-02	C9orf109
ILMN_1665148	3.631	3.849	-0.218	1.65	4.30E-02	LOC648249
ILMN_1782275	1.936	2.152	-0.216	1.65	2.02E-02	P2RY6
ILMN_1709039	2.074	2.290	-0.215	1.64	3.91E-02	RPL13
ILMN_1738657	1.891	2.103	-0.211	1.63	6.80E-03	SATB2
ILMN_1797362	1.957	2.166	-0.209	1.62	2.43E-02	LIMK1
ILMN_2206474	1.796	2.004	-0.208	1.61	2.30E-03	TMEM90B
ILMN_1760714	3.781	3.987	-0.206	1.61	3.67E-02	RPS3
ILMN_2389064	1.841	2.047	-0.205	1.60	4.03E-02	C15orf48
ILMN_1710885	3.798	3.999	-0.201	1.59	4.37E-02	LOC387867
ILMN_1788955	1.819	2.020	-0.201	1.59	1.70E-03	PDLIM1
ILMN_1775405	1.928	2.123	-0.195	1.57	4.56E-02	ARL4A
ILMN_2337058	1.995	2.189	-0.194	1.56	3.91E-02	PORCN
ILMN_1813379	1.904	2.095	-0.191	1.55	4.03E-02	TNFRSF9
ILMN_1687213	1.861	2.051	-0.190	1.55	1.45E-02	C8orf13
ILMN_1728218	1.893	2.082	-0.190	1.55	1.25E-02	RBPMS
ILMN_1738420	1.998	2.188	-0.190	1.55	4.82E-02	TMEM201
ILMN_1806576	1.943	2.131	-0.188	1.54	3.34E-02	LOC651137
ILMN_1762095	1.882	2.067	-0.185	1.53	1.56E-02	TMTC4
ILMN_1713401	1.901	2.086	-0.185	1.53	3.34E-02	LOC646607
ILMN_2325168	1.953	2.137	-0.184	1.53	4.51E-02	ARRB1
ILMN_1688886	1.888	2.071	-0.183	1.52	1.37E-02	GPC5
ILMN_3244640	1.936	2.115	-0.179	1.51	2.23E-02	SNORD96A
ILMN_1801307	1.878	2.054	-0.176	1.50	2.48E-02	TNFSF10
ILMN_1803686	1.884	2.060	-0.176	1.50	2.82E-02	ADA
ILMN_1658989	1.922	2.096	-0.173	1.49	2.78E-02	MEX3B

ILMN_1756928	1.805	1.978	-0.173	1.49	7.00E-04	RTN1
ILMN_1760849	1.824	1.997	-0.172	1.49	6.10E-03	NETO2
ILMN_1745599	1.906	2.071	-0.165	1.46	4.32E-02	LOC643977
ILMN_3240900	1.851	2.015	-0.164	1.46	1.87E-02	LOC100134728
ILMN_1728445	1.925	2.088	-0.164	1.46	4.51E-02	IGFBP1
ILMN_1745684	1.834	1.986	-0.152	1.42	4.00E-03	FLJ30375
ILMN_1761322	1.811	1.963	-0.151	1.42	1.24E-02	FHOD3
ILMN_1832106	1.891	2.036	-0.144	1.39	4.30E-02	TTC28
ILMN_1804339	1.833	1.972	-0.140	1.38	1.94E-02	CAMK1G
ILMN_2413779	1.753	1.891	-0.138	1.37	3.09E-02	SEZ6L2
ILMN_1668092	1.809	1.944	-0.135	1.36	2.09E-02	ESAM
ILMN_1813528	1.858	1.988	-0.130	1.35	3.83E-02	CCBE1
ILMN_1672626	1.848	1.976	-0.129	1.35	2.45E-02	GYPC
ILMN_1660200	1.863	1.983	-0.121	1.32	3.67E-02	LOC652175
ILMN_1817239	1.848	1.967	-0.119	1.31	4.09E-02	

Illumina ID	Mean(Normal)	Mean(Fibrillated	LogRatio	FoldChng	FDR	Symbol
ILMN_3239610	3.217	2.199	1.019	10.44	4.30E-03	LOC100133565
ILMN_2148527	3.695	2.974	0.721	5.26	4.35E-02	H19
ILMN_2412380	2.801	2.100	0.701	5.03	1.94E-02	TSC22D1
ILMN_3267017	3.422	2.732	0.690	4.90	1.74E-02	LOC100129028
ILMN_2210519	3.064	2.418	0.647	4.43	2.60E-03	HAPLN1
ILMN_2167805	3.435	2.792	0.643	4.39	1.00E-04	LUM
ILMN_1803788	3.084	2.451	0.634	4.30	0.00E+00	LGALS3
ILMN_1651228	3.607	3.033	0.574	3.75	2.70E-03	RPS28
ILMN_1789244	3.216	2.651	0.564	3.67	1.11E-02	SOX8
ILMN_1651699	2.881	2.319	0.563	3.65	3.00E-03	EPS8
ILMN_2122103	2.993	2.434	0.559	3.62	9.20E-03	ETS1
ILMN_3275345	3.566	3.014	0.552	3.56	4.10E-03	LOC100132291
ILMN_3271122	3.243	2.700	0.543	3.49	3.83E-02	LOC100129742
ILMN_1803647	2.672	2.132	0.540	3.47	3.20E-02	FAM162A
ILMN_2353161	2.410	1.871	0.539	3.46	1.10E-03	MSLN
ILMN_2181892	2.832	2.306	0.526	3.36	4.12E-02	BEX2
ILMN_3214532	3.287	2.767	0.520	3.31	4.66E-02	LOC100131205
ILMN_2275502	2.484	1.965	0.519	3.30	4.97E-02	RAPH1
ILMN_1675448	2.880	2.363	0.517	3.29	7.30E-03	ZFP36L1
ILMN_2056167	3.190	2.675	0.515	3.28	1.24E-02	OSTC
ILMN_2038775	3.196	2.689	0.507	3.21	1.40E-03	TUBB2A
ILMN_2371590	3.025	2.520	0.505	3.20	3.60E-03	DDX17
ILMN_2041101	3.449	2.945	0.504	3.19	6.20E-03	ANXA2P1
ILMN_3261439	2.977	2.474	0.503	3.19	4.75E-02	LOC100128098
ILMN_1664802	2.882	2.381	0.501	3.17	7.00E-04	WSB1
ILMN_3301065	3.835	3.337	0.498	3.15	4.99E-02	LOC728590
ILMN_1689725	2.839	2.356	0.484	3.05	2.39E-02	RPLP1
ILMN_2224103	2.539	2.058	0.482	3.03	2.09E-02	PAPSS1
ILMN_1731374	3.221	2.745	0.476	2.99	0.00E+00	CPE
ILMN_1768582	2.531	2.058	0.473	2.97	3.06E-02	PPP2CB
ILMN_1652073	3.694	3.238	0.457	2.86	2.38E-02	LOC653658
ILMN_2044226	2.412	1.959	0.453	2.84	3.10E-02	PPP3CA
ILMN_1674236	3.577	3.133	0.445	2.78	2.30E-03	HSPB1
ILMN_1808824	3.022	2.580	0.442	2.77	8.60E-03	NEBL
ILMN_1655913	3.257	2.817	0.440	2.75	3.00E-04	NUCB2
ILMN_3211906	3.334	2.896	0.438	2.74	4.21E-02	LOC647030
ILMN_3226875	3.427	2.990	0.437	2.74	2.48E-02	LOC728590
ILMN_1658015	2.462	2.028	0.435	2.72	1.76E-02	MBNL2
ILMN_2045419	2.512	2.078	0.434	2.72	6.60E-03	BNIP3L
ILMN_1814719	2.919	2.486	0.433	2.71	9.20E-03	RBP4
ILMN_1723123	3.286	2.854	0.432	2.71	7.00E-04	FGFR3
ILMN_2340935	3.228	2.800	0.428	2.68	2.08E-02	WBP5
ILMN_2148913	3.186	2.758	0.428	2.68	3.90E-03	TMEM45A
ILMN_1651799	3.411	2.983	0.428	2.68	0.00E+00	SLC38A2
ILMN_1775814	2.743	2.316	0.427	2.67	0.00E+00	GHR
ILMN_3238053	2.993	2.569	0.425	2.66	3.91E-02	LOC100129211
ILMN_1656386	2.754	2.331	0.423	2.65	7.30E-03	SEC24D
ILMN_1795243	3.090	2.672	0.419	2.62	4.97E-02	LOC220433
ILMN_3197767	2.851	2.434	0.418	2.62	3.56E-02	LOC645691
ILMN_1780170	3.891	3.474	0.417	2.61	4.00E-03	APOD
ILMN_1674620	2.914	2.500	0.414	2.60	1.30E-02	SGCE
ILMN_3195253	3.202	2.793	0.410	2.57	2.09E-02	LOC100130892
ILMN_3178252	3.404	2.994	0.409	2.57	3.86E-02	LOC100129379
ILMN_2364272	2.845	2.436	0.409	2.57	2.30E-03	MBNL2
ILMN_1692956	2.824	2.415	0.409	2.56	4.56E-02	LOC643949
ILMN_2050654	2.571	2.163	0.408	2.56	1.40E-03	SAV1
ILMN_3211887	2.753	2.346	0.408	2.56	3.69E-02	LOC727821

ILMN_1714709	2.921	2.513	0.408	2.56	0.00E+00	OLFM1
ILMN_1785703	2.991	2.586	0.406	2.55	3.06E-02	LMOD3
ILMN_2313158	2.937	2.532	0.406	2.54	7.00E-03	MBNL1
ILMN_2221006	2.647	2.242	0.405	2.54	3.75E-02	RAD21
ILMN_2192694	2.842	2.440	0.402	2.53	3.14E-02	EIF3M
ILMN_2374692	3.098	2.699	0.399	2.51	3.69E-02	WAC
ILMN_2181540	2.663	2.264	0.398	2.50	1.77E-02	YY1
ILMN_2202940	2.820	2.425	0.394	2.48	1.62E-02	CHPT1
ILMN_1680279	2.959	2.570	0.389	2.45	3.45E-02	USP49
ILMN_1696839	3.640	3.250	0.389	2.45	7.40E-03	RPS27
ILMN_1706117	3.624	3.237	0.387	2.44	3.65E-02	LOC391656
ILMN_3300471	3.252	2.868	0.384	2.42	2.08E-02	LOC730255
ILMN_1788778	2.737	2.355	0.382	2.41	1.28E-02	11-Sep
ILMN_2183331	2.511	2.129	0.382	2.41	1.04E-02	RBM7
ILMN_3178258	2.716	2.335	0.381	2.41	3.01E-02	FABP5L2
ILMN_1670439	2.757	2.376	0.381	2.40	9.80E-03	FYTTD1
ILMN_1693136	2.702	2.323	0.379	2.39	4.00E-03	VTI1B
ILMN_1689552	2.817	2.439	0.379	2.39	1.62E-02	FAM63A
ILMN_2262275	2.796	2.419	0.378	2.39	4.80E-02	TRIM13
ILMN_2106658	3.349	2.972	0.377	2.38	2.01E-02	BLZF1
ILMN_2328776	3.034	2.659	0.375	2.37	7.00E-04	MST4
ILMN_1689400	2.590	2.217	0.373	2.36	2.82E-02	CLK1
ILMN_1694810	2.506	2.133	0.373	2.36	2.50E-03	PANX2
ILMN_2204909	3.013	2.642	0.371	2.35	3.91E-02	XRCC2
ILMN_3244319	2.985	2.617	0.368	2.33	3.68E-02	CCDC125
ILMN_1792997	3.282	2.914	0.368	2.33	1.60E-03	NPTN
ILMN_1669788	2.629	2.263	0.366	2.33	2.50E-03	NUDT14
ILMN_1693421	3.017	2.652	0.365	2.32	0.00E+00	RPN2
ILMN_3265143	2.689	2.326	0.364	2.31	4.29E-02	LOC100129502
ILMN_1764090	2.912	2.548	0.364	2.31	1.10E-03	AK3L1
ILMN_1750100	2.539	2.176	0.363	2.31	3.85E-02	TUBB4Q
ILMN_1718136	3.604	3.242	0.362	2.30	3.14E-02	UQCRHL
ILMN_1698100	2.643	2.281	0.361	2.30	2.29E-02	ANXA2P1
ILMN_2079786	2.664	2.303	0.360	2.29	2.20E-03	NUAK1
ILMN_1855682	2.389	2.030	0.360	2.29	1.25E-02	
ILMN_2322806	2.567	2.208	0.359	2.28	1.40E-03	CAST
ILMN_2219712	2.545	2.186	0.358	2.28	4.72E-02	HMGB2
ILMN_2196347	2.439	2.081	0.358	2.28	1.72E-02	CDKN1B
ILMN_1700896	2.879	2.523	0.356	2.27	1.96E-02	SAP30
ILMN_1676159	3.294	2.938	0.355	2.27	1.10E-03	MST4
ILMN_1742025	3.220	2.866	0.355	2.26	5.00E-04	OLFM1
ILMN_1757702	2.704	2.349	0.355	2.26	4.46E-02	LOC647673
ILMN_3302499	2.630	2.276	0.354	2.26	9.00E-03	LOC730990
ILMN_3231550	2.752	2.399	0.354	2.26	2.83E-02	LOC100131718
ILMN_3231881	2.601	2.249	0.353	2.25	3.20E-02	LOC728026
ILMN_1782459	2.627	2.280	0.347	2.22	1.63E-02	OSBPL8
ILMN_3235803	2.306	1.960	0.347	2.22	9.80E-03	LOC653458
ILMN_1722532	2.461	2.115	0.346	2.22	7.00E-04	JMJD1A
ILMN_1802205	2.808	2.464	0.343	2.20	3.62E-02	RHOB
ILMN_2150465	3.135	2.791	0.343	2.20	5.20E-03	C5orf28
ILMN_3289262	2.775	2.434	0.341	2.19	2.02E-02	LOC100131261
ILMN_1679045	2.530	2.190	0.340	2.19	2.48E-02	SBDS
ILMN_2371055	2.983	2.644	0.340	2.19	4.60E-03	EFNA1
ILMN_1738095	2.701	2.364	0.337	2.17	1.00E-04	PER2
ILMN_2228732	2.349	2.013	0.336	2.17	2.30E-03	CCNG2
ILMN_1717180	2.536	2.200	0.336	2.17	3.14E-02	MTMR6
ILMN_1668861	2.506	2.171	0.335	2.16	1.33E-02	LOC732165
ILMN_1711408	2.228	1.896	0.332	2.15	2.48E-02	ANXA4

ILMN_1709479	2.983	2.652	0.330	2.14	1.26E-02	YAP1
ILMN_1663313	3.516	3.187	0.329	2.14	1.63E-02	AMY1C
ILMN_1765019	2.622	2.294	0.328	2.13	3.14E-02	SACM1L
ILMN_3264073	2.836	2.509	0.327	2.13	4.00E-03	LOC100130070
ILMN_3287093	3.606	3.278	0.327	2.12	1.72E-02	LOC646819
ILMN_3203444	2.898	2.572	0.327	2.12	3.00E-04	LOC100132535
ILMN_3243744	2.628	2.304	0.324	2.11	1.95E-02	EAPP
ILMN_3279712	2.873	2.553	0.320	2.09	3.00E-04	LOC642590
ILMN_1673421	2.839	2.521	0.318	2.08	3.23E-02	LOC440704
ILMN_1805668	3.541	3.225	0.315	2.07	4.26E-02	ZNF486
ILMN_1747744	3.149	2.834	0.315	2.07	1.26E-02	LHFPL2
ILMN_1665982	2.453	2.139	0.314	2.06	1.11E-02	AKTIP
ILMN_3237385	2.641	2.328	0.313	2.05	3.85E-02	NRBF2
ILMN_3274790	2.620	2.307	0.312	2.05	1.61E-02	LOC648921
ILMN_3200539	2.446	2.134	0.312	2.05	4.66E-02	LOC100131672
ILMN_1726308	2.881	2.570	0.311	2.05	3.89E-02	FAM10A4
ILMN_1803819	3.125	2.816	0.309	2.04	3.20E-03	IQGAP1
ILMN_1674706	3.040	2.731	0.309	2.04	2.23E-02	MTHFD2
ILMN_3293676	3.780	3.472	0.308	2.03	7.40E-03	LOC100131387
ILMN_1805800	2.622	2.314	0.308	2.03	9.20E-03	RAB5A
ILMN_3241234	3.409	3.102	0.307	2.03	1.61E-02	LOC730278
ILMN_1705111	2.541	2.234	0.307	2.03	1.24E-02	FNDC3A
ILMN_1693334	3.322	3.016	0.307	2.03	3.60E-03	P4HA1
ILMN_3210538	3.790	3.484	0.306	2.02	2.10E-03	LOC646785
ILMN_1694426	2.765	2.459	0.306	2.02	2.30E-03	ROR2
ILMN_1690894	2.389	2.084	0.305	2.02	2.91E-02	TRA1P2
ILMN_2186482	2.983	2.680	0.303	2.01	1.91E-02	TMED7
ILMN_1727738	2.162	1.859	0.303	2.01	2.20E-03	RAB33B
ILMN_2342841	2.449	2.147	0.301	2.00	1.89E-02	AFTPH
ILMN_2398107	2.904	2.605	0.299	1.99	3.56E-02	ASNS
ILMN_2154322	3.182	2.884	0.299	1.99	2.08E-02	SEMA3E
ILMN_3266944	2.568	2.270	0.298	1.99	1.63E-02	LOC100129599
ILMN_3297898	3.121	2.824	0.297	1.98	3.83E-02	LOC729769
ILMN_2117508	2.301	2.005	0.296	1.98	3.59E-02	CTHRC1
ILMN_2194009	2.468	2.172	0.296	1.98	1.04E-02	ABCC4
ILMN_1675646	2.253	1.957	0.295	1.97	3.34E-02	FN1
ILMN_2383913	2.457	2.163	0.294	1.97	1.56E-02	WDR33
ILMN_1787567	3.721	3.428	0.294	1.97	2.97E-02	TSC22D1
ILMN_3239895	2.375	2.082	0.294	1.97	3.09E-02	LOC100134053
ILMN_2373266	2.589	2.296	0.293	1.96	1.89E-02	SFRS12
ILMN_2124187	2.576	2.283	0.293	1.96	1.07E-02	TSC22D2
ILMN_1739259	2.629	2.337	0.292	1.96	4.21E-02	UBE4A
ILMN_3245517	2.607	2.316	0.291	1.96	1.12E-02	LOC100134273
ILMN_3300797	4.033	3.743	0.290	1.95	4.51E-02	LOC729090
ILMN_2338921	2.568	2.278	0.289	1.95	1.11E-02	C4orf41
ILMN_1666376	2.460	2.173	0.288	1.94	8.10E-03	TRIM56
ILMN_1667626	2.216	1.931	0.285	1.93	1.67E-02	EGLN3
ILMN_2102960	2.280	1.996	0.284	1.92	4.58E-02	KIAA1370
ILMN_1783852	2.222	1.938	0.283	1.92	3.35E-02	CD164
ILMN_1810838	3.077	2.793	0.283	1.92	1.96E-02	MTDH
ILMN_1681467	2.226	1.942	0.283	1.92	5.10E-03	RAB11FIP4
ILMN_2129234	2.818	2.535	0.283	1.92	3.06E-02	TMEM47
ILMN_1725387	2.302	2.020	0.282	1.91	1.30E-03	TMEM200A
ILMN_1734696	2.909	2.628	0.282	1.91	2.86E-02	FRG1
ILMN_1786972	3.115	2.834	0.282	1.91	1.67E-02	SARS
ILMN_2147105	2.828	2.547	0.281	1.91	4.70E-03	LOC440348
ILMN_2153332	2.343	2.063	0.280	1.91	4.03E-02	ATXN1
ILMN_1745110	3.014	2.734	0.280	1.91	3.13E-02	LAPTM4A

ILMN_2088825	2.402	2.122	0.280	1.91	1.93E-02	CENTB2
ILMN_1685140	2.879	2.599	0.280	1.90	2.90E-03	PRRC1
ILMN_1788701	2.482	2.203	0.280	1.90	3.96E-02	PSIP1
ILMN_2362581	2.553	2.275	0.278	1.89	1.66E-02	FNDC3A
ILMN_3243175	3.173	2.895	0.277	1.89	1.67E-02	LOC100132727
ILMN_1707312	3.096	2.821	0.276	1.89	2.09E-02	NFIL3
ILMN_2356955	2.215	1.941	0.273	1.88	7.90E-03	PLAGL1
ILMN_2059173	3.005	2.731	0.273	1.88	4.97E-02	SLC35E1
ILMN_3251620	2.274	2.001	0.273	1.87	4.46E-02	JMY
ILMN_2052208	2.840	2.567	0.273	1.87	1.88E-02	GADD45A
ILMN_3239548	2.414	2.141	0.272	1.87	4.09E-02	LOC100132740
ILMN_2054442	2.863	2.591	0.272	1.87	2.48E-02	ZNF146
ILMN_2097410	3.493	3.222	0.271	1.87	4.59E-02	DAPP1
ILMN_1691789	2.593	2.323	0.270	1.86	4.87E-02	SMNDC1
ILMN_2064132	2.141	1.872	0.269	1.86	1.83E-02	NANP
ILMN_1760490	3.621	3.352	0.269	1.86	1.37E-02	ACVR1
ILMN_2107613	2.309	2.039	0.269	1.86	7.40E-03	RHOJ
ILMN_1761131	3.034	2.765	0.269	1.86	1.25E-02	PECI
ILMN_2091375	2.483	2.214	0.269	1.86	1.24E-02	KRCC1
ILMN_1672947	2.258	1.990	0.268	1.85	3.58E-02	CAST
ILMN_3178792	3.037	2.769	0.268	1.85	3.06E-02	HNRNPA2B1
ILMN_1732216	3.318	3.052	0.266	1.85	2.02E-02	NARS
ILMN_1764396	2.224	1.958	0.266	1.84	3.83E-02	HDAC4
ILMN_1689976	2.786	2.520	0.266	1.84	1.11E-02	EDIL3
ILMN_2062381	2.335	2.070	0.265	1.84	3.20E-03	LCOR
ILMN_2331163	2.589	2.325	0.264	1.84	2.62E-02	CUL4A
ILMN_1664449	2.968	2.703	0.264	1.84	1.39E-02	ALG5
ILMN_1690114	2.573	2.310	0.263	1.83	4.51E-02	PTPLAD2
ILMN_2124155	2.255	1.993	0.262	1.83	3.41E-02	ATP11B
ILMN_1714759	3.357	3.096	0.261	1.83	1.70E-02	CNIH4
ILMN_2190414	2.543	2.282	0.261	1.82	1.11E-02	ZNF83
ILMN_2246548	2.390	2.130	0.260	1.82	3.43E-02	GSTTP2
ILMN_1703622	2.199	1.939	0.260	1.82	8.00E-04	PPIB
ILMN_1778202	2.634	2.375	0.260	1.82	3.93E-02	FLJ40722
ILMN_1761474	2.198	1.938	0.260	1.82	2.57E-02	LOC654189
ILMN_3241970	2.389	2.131	0.258	1.81	3.83E-02	POLR2J2
ILMN_3310416	2.325	2.068	0.257	1.81	3.20E-03	SNORD114-3
ILMN_1726693	2.296	2.039	0.257	1.81	5.10E-03	GTF2H1
ILMN_1785765	3.269	3.012	0.257	1.81	3.18E-02	TM9SF2
ILMN_1766000	2.277	2.020	0.257	1.81	2.80E-03	PM20D2
ILMN_1660549	2.420	2.165	0.255	1.80	1.77E-02	GPR177
ILMN_1806790	2.255	2.001	0.254	1.79	4.70E-03	ROBO1
ILMN_1720088	2.205	1.952	0.253	1.79	4.32E-02	SFRS12
ILMN_1805646	2.796	2.543	0.253	1.79	3.94E-02	SS18
ILMN_2223130	2.409	2.156	0.253	1.79	2.94E-02	SMARCA5
ILMN_1674128	2.159	1.906	0.253	1.79	3.14E-02	CWC22
ILMN_2230566	2.631	2.379	0.252	1.79	1.58E-02	RAB40B
ILMN_1732080	2.703	2.450	0.252	1.79	4.61E-02	SUMO1P3
ILMN_3201480	4.143	3.891	0.252	1.79	2.20E-03	LOC643358
ILMN_1808777	2.840	2.589	0.251	1.78	1.83E-02	EHD2
ILMN_1696975	2.669	2.418	0.251	1.78	1.53E-02	USP1
ILMN_3207738	2.489	2.238	0.251	1.78	4.54E-02	LOC646527
ILMN_1794187	2.357	2.106	0.251	1.78	1.26E-02	FBXL3
ILMN_3242462	2.231	1.983	0.249	1.77	2.10E-03	UHRF1BP1
ILMN_2392080	2.178	1.929	0.249	1.77	4.75E-02	DCAF6
ILMN_3291472	2.374	2.126	0.248	1.77	4.75E-02	LOC442727
ILMN_1733176	3.099	2.852	0.247	1.77	3.32E-02	LIMS1
ILMN_1696066	2.281	2.034	0.247	1.77	2.45E-02	CARS

ILMN_2096985	2.142	1.897	0.245	1.76	3.14E-02	ALDH6A1
ILMN_2386891	2.176	1.931	0.244	1.76	3.14E-02	GLT8D1
ILMN_1694075	2.699	2.455	0.244	1.75	4.48E-02	GADD45A
ILMN_1688702	3.702	3.458	0.244	1.75	2.39E-02	PJA2
ILMN_3240117	3.104	2.860	0.244	1.75	3.67E-02	AIDA
ILMN_2126832	2.308	2.064	0.244	1.75	3.34E-02	SEC24A
ILMN_2151441	2.434	2.192	0.242	1.75	1.61E-02	FAM103A1
ILMN_3289171	2.209	1.969	0.240	1.74	1.57E-02	LOC100131572
ILMN_2398388	2.273	2.034	0.238	1.73	1.04E-02	APH1A
ILMN_1661917	4.099	3.863	0.237	1.72	9.20E-03	LOC644039
ILMN_1707337	2.337	2.101	0.236	1.72	3.10E-02	MSTO1
ILMN_1652955	3.490	3.254	0.236	1.72	4.66E-02	LOC648622
ILMN_2142979	2.337	2.101	0.236	1.72	2.02E-02	PTBP2
ILMN_1658531	2.338	2.103	0.235	1.72	2.64E-02	LOC653702
ILMN_1775962	2.434	2.199	0.234	1.72	3.86E-02	MCOLN3
ILMN_2395728	2.901	2.666	0.235	1.72	3.42E-02	HNRPUL1
ILMN_2063586	3.759	3.525	0.234	1.71	3.54E-02	CLIC4
ILMN_2320906	2.186	1.953	0.233	1.71	7.00E-03	RTN3
ILMN_2353358	2.453	2.221	0.232	1.71	4.12E-02	LGALS8
ILMN_2137536	2.738	2.509	0.230	1.70	4.75E-02	ZZZ3
ILMN_2182704	3.356	3.127	0.229	1.70	4.67E-02	BIRC2
ILMN_2083243	2.434	2.205	0.229	1.69	3.10E-02	MNAT1
ILMN_3307659	2.529	2.301	0.228	1.69	3.56E-02	SFT2D2
ILMN_2346137	2.409	2.181	0.227	1.69	2.51E-02	ZNF557
ILMN_1796537	2.098	1.872	0.226	1.68	4.00E-03	FYB
ILMN_1811029	2.401	2.176	0.225	1.68	2.62E-02	TLK1
ILMN_1775829	2.189	1.963	0.225	1.68	1.24E-02	PERP
ILMN_2182348	2.056	1.831	0.225	1.68	1.02E-02	SMC3
ILMN_2406132	3.995	3.772	0.223	1.67	2.60E-02	LILRB3
ILMN_1678437	2.082	1.860	0.222	1.67	4.61E-02	FRY
ILMN_2204664	2.225	2.005	0.220	1.66	8.40E-03	NBPF14
ILMN_1881960	2.194	1.974	0.220	1.66	2.95E-02	
ILMN_2358980	2.394	2.175	0.219	1.66	3.15E-02	ILK
ILMN_2076250	2.113	1.898	0.215	1.64	1.61E-02	GPBP1L1
ILMN_2382354	2.389	2.178	0.211	1.63	4.44E-02	SENP7
ILMN_1683959	2.204	1.994	0.210	1.62	3.67E-02	MED13L
ILMN_1685978	2.367	2.158	0.209	1.62	4.51E-02	ATPIF1
ILMN_2145997	2.297	2.091	0.206	1.61	3.83E-02	SP4
ILMN_1776487	2.257	2.052	0.205	1.60	3.09E-02	TADA1L
ILMN_3282395	2.238	2.034	0.204	1.60	2.97E-02	LOC646966
ILMN_1672661	2.240	2.037	0.203	1.59	3.36E-02	SP110
ILMN_3251436	2.118	1.916	0.202	1.59	2.97E-02	DENND4C
ILMN_1771286	2.186	1.985	0.201	1.59	2.44E-02	LOC653513
ILMN_3305899	2.129	1.930	0.199	1.58	1.24E-02	LOC728572
ILMN_1702946	2.252	2.054	0.198	1.58	3.69E-02	THUMPD1
ILMN_1786359	2.211	2.016	0.195	1.57	4.29E-02	LOC346950
ILMN_1801923	2.226	2.033	0.193	1.56	4.56E-02	ATF1
ILMN_1736068	2.150	1.958	0.192	1.56	3.69E-02	CNOT8
ILMN_2196335	2.162	1.972	0.190	1.55	2.43E-02	C12orf11
ILMN_1776640	1.992	1.805	0.188	1.54	5.60E-03	MPL
ILMN_1781173	2.002	1.817	0.186	1.53	1.48E-02	HDAC9
ILMN_2100258	2.140	1.956	0.183	1.52	3.83E-02	PCDHB16
ILMN_1761833	2.096	1.918	0.177	1.50	1.94E-02	SLC40A1
ILMN_2414366	2.041	1.868	0.173	1.49	1.11E-02	KAT5
ILMN_2223720	2.143	1.977	0.166	1.47	3.83E-02	ATMIN
ILMN_3288529	2.127	1.964	0.163	1.46	4.88E-02	LOC645630
ILMN_2232494	1.995	1.833	0.162	1.45	2.29E-02	C5orf42
ILMN_1683129	2.095	1.937	0.158	1.44	4.56E-02	CCNL1

ILMN_1743067	2.072	1.920	0.152	1.42	3.83E-02	ZNF701
ILMN_1730303	2.066	1.913	0.152	1.42	3.54E-02	KBTBD7
ILMN_3276005	2.077	1.929	0.148	1.41	4.83E-02	LOC648283
ILMN_1723969	2.045	1.905	0.141	1.38	2.48E-02	PLCB1
ILMN_1805765	1.968	1.832	0.136	1.37	1.39E-02	CMYA5
ILMN_1716736	1.981	1.846	0.135	1.36	1.42E-02	CD80
ILMN_1792419	1.957	1.824	0.133	1.36	3.13E-02	FRMD7
ILMN_3294741	2.012	1.886	0.126	1.34	4.24E-02	LOC644496
ILMN_1784130	1.963	1.840	0.123	1.33	3.83E-02	LOC647054
ILMN_3243248	1.984	1.865	0.119	1.31	4.98E-02	SNORD87
