# Supplementary material for: Transcriptome-Wide Analysis of Messenger RNA Decay in Normal and Osteoarthritic Human Articular Chondrocytes
Source: Arthritis Rheumatol. 2014 Oct 26;66(11):3052–61. doi: 10.1002/art.38849 (PMC4233952; doi:10.1002/art.38849)
Supplement: Supplementary file 2 [file art0066-3052-sd2.pdf]

Tew et al - Transcriptome-wide analysis of mRNA decay in normal and osteoarthritic human articular chondrocytes

Supplemental Data 2

Short Lived mRNAs - 284 mRNAs identified as commonly having a half life of < 6 hours in intact normal, intact OA and fibrillated OA cells. This constitutes
Column names are self explanatory for this data

GeneSet2miRNA Output - results of analysis of short lived mRNAs with the Gene set 2miRNA tool at http://www.bioprofiling.de/index.html
Column Names:
# - Rank in analysis (sorted by p value)
microRNA name - - note the number after the dot refers to whether the miRNA was identified under "soft" (4) or "strict" (5) threshold.
I_A - The number of genes from the input list predicted to be the target of the MicroRNA (in brackets the size of the input list is reported)
I_B - The total number of genes predicted to be the target of the MicroRNA (in brackets the number of annotated genes in the whole genome is given)
Odds Ratio - (I A/size_input_list)/(I B/size_reference_list) The ratio of occurrence for "MicroRNA model" in the input list to the occurrence for "MicroRNA mod e
p - value - The P-value of the enrichment (adjusted for multiple testing by Bonferroni correction or Monte-Carlo simulations)
Gene - Genes identified from input list as being targets of the microRNA

mRNAs with half life <6 hours in all samples examined

Description	Illumina ID	Gene Name
nuclear receptor interacting protein 1 [Source:HGNC Symbol;Acc:8001]	ILMN_1718629	NRIP1
INO80 complex subunit [Source:HGNC Symbol;Acc:26956]	ILMN_1678362	INO80
KN motif and ankyrin repeat domains 1 [Source:HGNC Symbol;Acc:19309]	ILMN_1773427	KANK1
RNA terminal phosphate cyclase-like 1 [Source:HGNC Symbol;Acc:17687]	ILMN_1813766	RCL1
	ILMN_3255792	RP11-264C15.2
T-cell lymphoma invasion and metastasis 2 [Source:HGNC Symbol;Acc:11806]	ILMN_2358560	TIAM2
pleckstrin homology domain containing, family O member 2 [Source:HGNC Symbol;Acc:30026]	ILMN_1689968	PLEKHO2
protein tyrosine phosphatase, receptor type, E [Source:HGNC Symbol;Acc:9669]	ILMN_2383611	PTPRE
dual specificity phosphatase 14 [Source:HGNC Symbol;Acc:17007]	ILMN_1666546	DUSP14
ras homolog family member B [Source:HGNC Symbol;Acc:668]	ILMN_1802205	RHOB
mitogen-activated protein kinase 6 [Source:HGNC Symbol;Acc:6879]	ILMN_1757287	MAPK6
BTG family, member 2 [Source:HGNC Symbol;Acc:1131]	ILMN_1770085	BTG2
vestigial like 4 (Drosophila) [Source:HGNC Symbol;Acc:28966]	ILMN_1768480	VGLL4
nuclear receptor coactivator 5 [Source:HGNC Symbol;Acc:15909]	ILMN_1770035	NCOA5
leucine rich repeat containing 37B pseudogene 1 [Source:HGNC Symbol;Acc:25390]	ILMN_3245600	LRRC37BP1
ankyrin repeat domain 17 [Source:HGNC Symbol;Acc:23575]	ILMN_1712019	ANKRD17
BCL2-associated athanogene 3 [Source:HGNC Symbol;Acc:939]	ILMN_1659766	BAG3
Myb/SANT-like DNA-binding domain containing 4 with coiled-coils [Source:HGNC Symbol;Acc:29383]	ILMN_1801124	MSANTD4
Rap guanine nucleotide exchange factor (GEF) 2 [Source:HGNC Symbol;Acc:16854]	ILMN_3247261	RAPGEF2
ring finger protein 144B [Source:HGNC Symbol;Acc:21578]	ILMN_1752526	RNF144B
SAM and SH3 domain containing 1 [Source:HGNC Symbol;Acc:19182]	ILMN_2185984	SASH1
interferon regulatory factor 2 binding protein-like [Source:HGNC Symbol;Acc:14282]	ILMN_1804396	IRF2BPL
KIAA1737 [Source:HGNC Symbol;Acc:20365]	ILMN_2097858	KIAA1737
	ILMN_1704418	AC099522.1
tuftelin interacting protein 11 [Source:HGNC Symbol;Acc:17165]	ILMN_2408102	TFIP11
FOS-like antigen 1 [Source:HGNC Symbol;Acc:13718]	ILMN_1771841	FOSL1
insulin receptor substrate 1 [Source:HGNC Symbol;Acc:6125]	ILMN_1759232	IRS1
zinc finger, MIZ-type containing 1 [Source:HGNC Symbol;Acc:16493]	ILMN_1771627	ZMIZ1
SMAD family member 4 [Source:HGNC Symbol;Acc:6770]	ILMN_1741477	SMAD4
THAP domain containing 11 [Source:HGNC Symbol;Acc:23194]	ILMN_1780699	THAP11
	ILMN_3284366	RP11-153M3.1
adrenomedullin [Source:HGNC Symbol;Acc:259]	ILMN_1708934	ADM
Cbp/p300-interacting transactivator, with Glu/Asp-rich carboxy-terminal domain, 2 [Source:HGNC Symbol;Acc:1987]	ILMN_1663092	CITED2
CCR4-NOT transcription complex, subunit 4 [Source:HGNC Symbol;Acc:7880]	ILMN_1772677	CNOT4
X-box binding protein 1 [Source:HGNC Symbol;Acc:12801]	ILMN_1809433	XBP1
X-box binding protein 1 [Source:HGNC Symbol;Acc:12801]	ILMN_2365465	XBP1
Rho GTPase activating protein 21 [Source:HGNC Symbol;Acc:23725]	ILMN_1811592	ARHGAP21
SERTA domain containing 1 [Source:HGNC Symbol;Acc:17932]	ILMN_1794017	SERTAD1
zinc finger CCCH-type containing 4 [Source:HGNC Symbol;Acc:17808]	ILMN_2087575	ZC3H4
RAB11 family interacting protein 5 (class I) [Source:HGNC Symbol;Acc:24845]	ILMN_1740604	RAB11FIP5
regulator of calcineurin 1 [Source:HGNC Symbol;Acc:3040]	ILMN_2367239	RCAN1
forkhead box D2 [Source:HGNC Symbol;Acc:3803]	ILMN_1789400	FOXD2
limb bud and heart development [Source:HGNC Symbol;Acc:29532]	ILMN_2315979	LBH
runt-related transcription factor 1 [Source:HGNC Symbol;Acc:10471]	ILMN_1801504	RUNX1
inhibitor of DNA binding 3, dominant negative helix-loop-helix protein [Source:HGNC Symbol;Acc:5362]	ILMN_1732296	ID3
basic helix-loop-helix family, member e40 [Source:HGNC Symbol;Acc:1046]	ILMN_1768534	BHLHE40
zinc finger, BED-type containing 5 [Source:HGNC Symbol;Acc:30803]	ILMN_1664424	ZBED5
StAR-related lipid transfer (START) domain containing 13 [Source:HGNC Symbol;Acc:19164]	ILMN_2341254	STARD13
translocation associated membrane protein 2 [Source:HGNC Symbol;Acc:16855]	ILMN_1788783	TRAM2
Yae1 domain containing 1 [Source:HGNC Symbol;Acc:24857]	ILMN_1793508	YAE1D1
angiopoietin-like 4 [Source:HGNC Symbol;Acc:16039]	ILMN_1707727	ANGPTL4
perilipin 2 [Source:HGNC Symbol;Acc:248]	ILMN_2138765	PLIN2
CDC42 effector protein (Rho GTPase binding) 4 [Source:HGNC Symbol;Acc:17147]	ILMN_1745223	CDC42EP4
SRY (sex determining region Y)-box 8 [Source:HGNC Symbol;Acc:11203]	ILMN_1789244	SOX8
bone morphogenetic protein 2 [Source:HGNC Symbol;Acc:1069]	ILMN_1722718	BMP2
forkhead box J2 [Source:HGNC Symbol;Acc:24818]	ILMN_1731648	FOXJ2
chondroitin sulfate synthase 1 [Source:HGNC Symbol;Acc:17198]	ILMN_1791576	CHSY1
solute carrier family 20 (phosphate transporter), member 1 [Source:HGNC Symbol;Acc:10946]	ILMN_1672662	SLC20A1
kelch-like family member 9 [Source:HGNC Symbol;Acc:18732]	ILMN_1664466	KLHL9
zinc finger CCCH-type containing 10 [Source:HGNC Symbol;Acc:25893]	ILMN_2075847	ZC3H10
chromosome 10 open reading frame 10 [Source:HGNC Symbol;Acc:23355]	ILMN_1767556	C10orf10
SMAD family member 6 [Source:HGNC Symbol;Acc:6772]	ILMN_1767068	SMAD6
	ILMN_1844692	RP11-815I9.4
zinc finger protein 503 [Source:HGNC Symbol;Acc:23589]	ILMN_1787265	ZNF503
zinc finger protein 22 [Source:HGNC Symbol;Acc:13012]	ILMN_1798533	ZNF22
zinc finger protein 22 [Source:HGNC Symbol;Acc:13012]	ILMN_2117904	ZNF22
RAMP2 antisense RNA 1 [Source:HGNC Symbol;Acc:44358]	ILMN_3294106	RAMP2-AS1
	ILMN_2315979	AC104698.1
forkhead box O1 [Source:HGNC Symbol;Acc:3819]	ILMN_1738816	FOXO1
upregulator of cell proliferation [Source:HGNC Symbol;Acc:30890]	ILMN_2386818	URGCP
Dab, mitogen-responsive phosphoprotein, homolog 2 (Drosophila) [Source:HGNC Symbol;Acc:2662]	ILMN_2128428	DAB2
Dab, mitogen-responsive phosphoprotein, homolog 2 (Drosophila) [Source:HGNC Symbol;Acc:2662]	ILMN_1764228	DAB2
peroxisome proliferator-activated receptor gamma, coactivator-related 1 [Source:HGNC Symbol;Acc:30025]	ILMN_1796210	PPRC1
mitogen-activated protein kinase kinase kinase 8 [Source:HGNC Symbol;Acc:6860]	ILMN_1741159	MAP3K8
Nipped-B homolog (Drosophila) [Source:HGNC Symbol;Acc:28862]	ILMN_2264625	NIPBL
B-cell CLL/lymphoma 3 [Source:HGNC Symbol;Acc:998]	ILMN_1710514	BCL3
forkhead box O3 [Source:HGNC Symbol;Acc:3821]	ILMN_1844692	FOXO3
GTP binding protein overexpressed in skeletal muscle [Source:HGNC Symbol;Acc:4234]	ILMN_1677092	GEM
GTP binding protein overexpressed in skeletal muscle [Source:HGNC Symbol;Acc:4234]	ILMN_2367883	GEM
Ras association (RalGDS/AF-6) and pleckstrin homology domains 1 [Source:HGNC Symbol;Acc:14436]	ILMN_1783846	RAPH1
TSC22 domain family, member 1 [Source:HGNC Symbol;Acc:16826]	ILMN_1787567	TSC22D1
HCG20425, isoform CRA_a; Uncharacterized protein; cDNA FLJ53815 [Source:UniProtKB/TrEMBL;Acc:B4DNA4]	ILMN_3244323	RP4-758J18.2
nucleoporin 153kDa [Source:HGNC Symbol;Acc:8062]	ILMN_1705907	NUP153
immediate early response 5-like [Source:HGNC Symbol;Acc:23679]	ILMN_1755620	IER5L
leucine carboxyl methyltransferase 2 [Source:HGNC Symbol;Acc:17558]	ILMN_1667577	LCMT2
K(lysine) acetyltransferase 6A [Source:HGNC Symbol;Acc:13013]	ILMN_2095840	KAT6A
CCAAT/enhancer binding protein (C/EBP), beta [Source:HGNC Symbol;Acc:1834]	ILMN_1693014	CEBPB
nitric oxide synthase 2, inducible [Source:HGNC Symbol;Acc:7873]	ILMN_3249361	NOS2
nitric oxide synthase 2, inducible [Source:HGNC Symbol;Acc:7873]	ILMN_2322831	NOS2
protein phosphatase 1, regulatory subunit 3C [Source:HGNC Symbol;Acc:9293]	ILMN_1736670	PPP1R3C
SERTA domain containing 2 [Source:HGNC Symbol;Acc:30784]	ILMN_1651347	SERTAD2
myosin X [Source:HGNC Symbol;Acc:7593]	ILMN_2232712	MYO10
nicotinamide N-methyltransferase [Source:HGNC Symbol;Acc:7861]	ILMN_1715508	NNMT
MROH7-TTC4 readthrough (NMD candidate) [Source:HGNC Symbol;Acc:49180]	ILMN_1678140	MROH7-TTC4
MID1 interacting protein 1 [Source:HGNC Symbol;Acc:20715]	ILMN_2165473	MID1IP1
tetratricopeptide repeat domain 4 [Source:HGNC Symbol;Acc:12394]	ILMN_1678140	TTC4
inhibitor of DNA binding 2, dominant negative helix-loop-helix protein [Source:HGNC Symbol;Acc:5361]	ILMN_2086095	ID2
inhibitor of DNA binding 2, dominant negative helix-loop-helix protein [Source:HGNC Symbol;Acc:5361]	ILMN_1793990	ID2
pleckstrin homology domain containing, family F (with FYVE domain) member 1 [Source:HGNC Symbol;Acc:20764]	ILMN_1708041	PLEKHF1
tripartite motif containing 47 [Source:HGNC Symbol;Acc:19020]	ILMN_1712708	TRIM47
GLTSCR1-like [Source:HGNC Symbol;Acc:21111]	ILMN_1696127	GLTSCR1L
B-cell translocation gene 1, anti-proliferative [Source:HGNC Symbol;Acc:1130]	ILMN_1775743	BTG1
heme oxygenase (decycling) 1 [Source:HGNC Symbol;Acc:5013]	ILMN_1800512	HMOX1
family with sequence similarity 46, member A [Source:HGNC Symbol;Acc:18345]	ILMN_1740466	FAM46A
ankyrin repeat domain 37 [Source:HGNC Symbol;Acc:29593]	ILMN_1756417	ANKRD37
La ribonucleoprotein domain family, member 6 [Source:HGNC Symbol;Acc:24012]	ILMN_1752810	LARP6

mRNAs with half life <6 hours in all samples examined

Norrie disease (pseudoglioma) [Source:HGNC Symbol;Acc:7678]	ILMN_1794803	NDP
kelch-like ECH-associated protein 1 [Source:HGNC Symbol;Acc:23177]	ILMN_2410771	KEAP1
TSPY-like 5 [Source:HGNC Symbol;Acc:29367]	ILMN_1737972	TSPYL5
forkhead box C1 [Source:HGNC Symbol;Acc:3800]	ILMN_1738401	FOXC1
phosphodiesterase 4B, cAMP-specific [Source:HGNC Symbol;Acc:8781]	ILMN_2340259	PDE4B
CCAAT/enhancer binding protein (C/EBP), delta [Source:HGNC Symbol;Acc:1835]	ILMN_1782050	CEBPD
adrenoceptor beta 2, surface [Source:HGNC Symbol;Acc:286]	ILMN_1695590	ADRB2
zinc finger protein 876, pseudogene [Source:HGNC Symbol;Acc:32472]	ILMN_3226663	ZNF876P
zinc finger protein 263 [Source:HGNC Symbol;Acc:13056]	ILMN_1692620	ZNF263
DNA meiotic recombinase 1 [Source:HGNC Symbol;Acc:2927]	ILMN_2162367	DMC1
TRAF3 interacting protein 2 [Source:HGNC Symbol;Acc:1343]	ILMN_1701514	TRAF3IP2
	ILMN_3227321	RP11-747H12.5
sprouty homolog 2 (Drosophila) [Source:HGNC Symbol;Acc:11270]	ILMN_2089329	SPRY2
snail family zinc finger 2 [Source:HGNC Symbol;Acc:11094]	ILMN_1655740	SNAI2
snail family zinc finger 2 [Source:HGNC Symbol;Acc:11094]	ILMN_2082585	SNAI2
forkhead box D1 [Source:HGNC Symbol;Acc:3802]	ILMN_1704418	FOXD1
dual specificity phosphatase 1 [Source:HGNC Symbol;Acc:3064]	ILMN_1781285	DUSP1
B-cell CLL/lymphoma 6 [Source:HGNC Symbol;Acc:1001]	ILMN_1737314	BCL6
Kruppel-like factor 9 [Source:HGNC Symbol;Acc:1123]	ILMN_1778523	KLF9
XK, Kell blood group complex subunit-related family, member 8 [Source:HGNC Symbol;Acc:25508]	ILMN_2087303	XKR8
vacuolar protein sorting 37 homolog B (S. cerevisiae) [Source:HGNC Symbol;Acc:25754]	ILMN_1710427	VPS37B
RNA binding motif protein 23 [Source:HGNC Symbol;Acc:20155]	ILMN_2363106	RBM23
RNA binding motif protein 23 [Source:HGNC Symbol;Acc:20155]	ILMN_1780756	RBM23
inhibitor of DNA binding 1, dominant negative helix-loop-helix protein [Source:HGNC Symbol;Acc:5360]	ILMN_1664861	ID1
BCL2-like 1 [Source:HGNC Symbol;Acc:992]	ILMN_1654118	BCL2L1
zinc finger, AN1-type domain 5 [Source:HGNC Symbol;Acc:13008]	ILMN_1795228	ZFAND5
mutS homolog 6 [Source:HGNC Symbol;Acc:7329]	ILMN_1729051	MSH6
glucosaminyl (N-acetyl) transferase 1, core 2 [Source:HGNC Symbol;Acc:4203]	ILMN_1666545	GCNT1
inositol 1,4,5-trisphosphate receptor interacting protein [Source:HGNC Symbol;Acc:29370]	ILMN_1805192	ITPRIP
inositol 1,4,5-trisphosphate receptor interacting protein [Source:HGNC Symbol;Acc:29370]	ILMN_3239181	ITPRIP
pelota homolog (Drosophila) [Source:HGNC Symbol;Acc:8829]	ILMN_1770811	PELO
AT rich interactive domain 5B (MRF1-like) [Source:HGNC Symbol;Acc:17362]	ILMN_1721626	ARID5B
kelch-like family member 21 [Source:HGNC Symbol;Acc:29041]	ILMN_1692785	KLHL21
cysteine-rich, angiogenic inducer, 61 [Source:HGNC Symbol;Acc:2654]	ILMN_2188264	CYR61
nuclear receptor binding factor 2 [Source:HGNC Symbol;Acc:19692]	ILMN_3237385	NRBF2
chromodomain helicase DNA binding protein 9 [Source:HGNC Symbol;Acc:25701]	ILMN_1762972	CHD9
chromosome 16 open reading frame 72 [Source:HGNC Symbol;Acc:30103]	ILMN_1773407	C16orf72
CXXC finger protein 5 [Source:HGNC Symbol;Acc:26943]	ILMN_1745256	CXXC5
CXXC finger protein 5 [Source:HGNC Symbol;Acc:26943]	ILMN_3307729	CXXC5
TD (carboxy-terminal domain, RNA polymerase II, polypeptide A) small phosphatase 2 [Source:HGNC Symbol;Acc:1707	ILMN_1692962	CTDSP2
ERBB receptor feedback inhibitor 1 [Source:HGNC Symbol;Acc:18185]	ILMN_1665510	ERRFI1
succinate dehydrogenase complex assembly factor 1 [Source:HGNC Symbol;Acc:33867]	ILMN_2070355	SDHAF1
growth arrest-specific 1 [Source:HGNC Symbol;Acc:4165]	ILMN_1772910	GAS1
splA/ryanodine receptor domain and SOCS box containing 1 [Source:HGNC Symbol;Acc:30628]	ILMN_1714170	SPSB1
IMP3, U3 small nucleolar ribonucleoprotein, homolog (yeast) [Source:HGNC Symbol;Acc:14497]	ILMN_1733696	IMP3
nuclear factor, interleukin 3 regulated [Source:HGNC Symbol;Acc:7787]	ILMN_1707312	NFIL3
syntabulin (syntaxin-interacting) [Source:HGNC Symbol;Acc:26011]	ILMN_1738989	SYBU
lysine-rich coiled-coil 1 [Source:HGNC Symbol;Acc:28039]	ILMN_2091375	KRCC1
lysine-rich coiled-coil 1 [Source:HGNC Symbol;Acc:28039]	ILMN_1745620	KRCC1
leucine zipper, down-regulated in cancer 1-like [Source:HGNC Symbol;Acc:13343]	ILMN_1691425	LDOC1L
hypoxia inducible lipid droplet-associated [Source:HGNC Symbol;Acc:28859]	ILMN_1659990	HILPDA
cysteine-rich hydrophobic domain 2 [Source:HGNC Symbol;Acc:1935]	ILMN_1679428	CHIC2
PHD finger protein 21A [Source:HGNC Symbol;Acc:24156]	ILMN_1699496	PHF21A
ubiquitin C [Source:HGNC Symbol;Acc:12468]	ILMN_2038773	UBC
ubiquitin C [Source:HGNC Symbol;Acc:12468]	ILMN_2331501	UBC
ring finger protein 216 pseudogene 1 [Source:HGNC Symbol;Acc:33610]	ILMN_3225534	RNF216P1
growth arrest and DNA-damage-inducible, beta [Source:HGNC Symbol;Acc:4096]	ILMN_1718977	GADD45B
tumor necrosis factor receptor superfamily, member 1B [Source:HGNC Symbol;Acc:11917]	ILMN_1764788	TNFRSF1B
Kruppel-like factor 4 (gut) [Source:HGNC Symbol;Acc:6348]	ILMN_1779857	KLF4
Kruppel-like factor 4 (gut) [Source:HGNC Symbol;Acc:6348]	ILMN_2137789	KLF4
AAR2 splicing factor homolog (S. cerevisiae) [Source:HGNC Symbol;Acc:15886]	ILMN_1721225	AAR2
FERM domain containing 6 [Source:HGNC Symbol;Acc:19839]	ILMN_2330787	FRMD6
FERM domain containing 6 [Source:HGNC Symbol;Acc:19839]	ILMN_1769282	FRMD6
BRF2, RNA polymerase III transcription initiation factor 50 kDa subunit [Source:HGNC Symbol;Acc:17298]	ILMN_1665554	BRF2
lipoma HMGIC fusion partner-like 2 [Source:HGNC Symbol;Acc:6588]	ILMN_1747744	LHFPL2
6-phosphofructo-2-kinase/fructose-2,6-biphosphatase 4 [Source:HGNC Symbol;Acc:8875]	ILMN_1653292	PFKFB4
v-ets avian erythroblastosis virus E26 oncogene homolog 1 [Source:HGNC Symbol;Acc:3488]	ILMN_2122103	ETS1
v-ets avian erythroblastosis virus E26 oncogene homolog 1 [Source:HGNC Symbol;Acc:3488]	ILMN_1687538	ETS1
Rho/Rac guanine nucleotide exchange factor (GEF) 2 [Source:HGNC Symbol;Acc:682]	ILMN_1703477	ARHGEF2
E3 ubiquitin-protein ligase RNF31 [Source:UniProtKB/TrEMBL;Acc:H0YM83]	ILMN_1745471	RP11-468E2.4
interferon regulatory factor 9 [Source:HGNC Symbol;Acc:6131]	ILMN_1745471	IRF9
arginine vasopressin-induced 1 [Source:HGNC Symbol;Acc:30898]	ILMN_1671731	AVPI1
6-phosphofructo-2-kinase/fructose-2,6-biphosphatase 3 [Source:HGNC Symbol;Acc:8874]	ILMN_2186061	PFKFB3
RAS, dexamethasone-induced 1 [Source:HGNC Symbol;Acc:15828]	ILMN_1740426	RASD1
Kruppel-like factor 2 (lung) [Source:HGNC Symbol;Acc:6347]	ILMN_1735930	KLF2
DNA-damage-inducible transcript 4-like [Source:HGNC Symbol;Acc:30555]	ILMN_1696537	DDIT4L
tumor necrosis factor receptor superfamily, member 1A [Source:HGNC Symbol;Acc:11916]	ILMN_1685005	TNFRSF1A
nuclear factor of kappa light polypeptide gene enhancer in B-cells inhibitor, zeta [Source:HGNC Symbol;Acc:29805]	ILMN_1719695	NFKBIZ
spen homolog, transcriptional regulator (Drosophila) [Source:HGNC Symbol;Acc:17575]	ILMN_1802611	SPEN
v-maf avian musculoaponeurotic fibrosarcoma oncogene homolog B [Source:HGNC Symbol;Acc:6408]	ILMN_1764709	MAFB
A kinase (PRKA) anchor protein 13 [Source:HGNC Symbol;Acc:371]	ILMN_2396956	AKAP13
SCO2 cytochrome c oxidase assembly protein [Source:HGNC Symbol;Acc:10604]	ILMN_1701621	SCO2
autism susceptibility candidate 2 [Source:HGNC Symbol;Acc:14262]	ILMN_1749081	AUTS2
serum/glucocorticoid regulated kinase 1 [Source:HGNC Symbol;Acc:10810]	ILMN_3229324	SGK1
serum/glucocorticoid regulated kinase 1 [Source:HGNC Symbol;Acc:10810]	ILMN_3305938	SGK1
serum/glucocorticoid regulated kinase 1 [Source:HGNC Symbol;Acc:10810]	ILMN_1702487	SGK1
distal-less homeobox 5 [Source:HGNC Symbol;Acc:2918]	ILMN_1759598	DLX5
ST3 beta-galactoside alpha-2,3-sialyltransferase 1 [Source:HGNC Symbol;Acc:10862]	ILMN_1683313	ST3GAL1
platelet-activating factor acetylhydrolase 1b, regulatory subunit 1 (45kDa) [Source:HGNC Symbol;Acc:8574]	ILMN_1722276	PAFAH1B1
nerve growth factor (beta polypeptide) [Source:HGNC Symbol;Acc:7808]	ILMN_1716608	NGF
T-box 15 [Source:HGNC Symbol;Acc:11594]	ILMN_1721580	TBX15
baculoviral IAP repeat containing 3 [Source:HGNC Symbol;Acc:591]	ILMN_2405684	BIRC3
potassium channel tetramerization domain containing 6 [Source:HGNC Symbol;Acc:22235]	ILMN_2146372	KCTD6
Rho family GTPase 3 [Source:HGNC Symbol;Acc:671]	ILMN_1759513	RND3
thioredoxin interacting protein [Source:HGNC Symbol;Acc:16952]	ILMN_1697448	TXNIP
HMG-box transcription factor 1 [Source:HGNC Symbol;Acc:23200]	ILMN_1685415	HBP1
peroxisomal biogenesis factor 11 beta [Source:HGNC Symbol;Acc:8853]	ILMN_1678546	PEX11B
SIX homeobox 4 [Source:HGNC Symbol;Acc:10890]	ILMN_1743402	SIX4
adhesion molecule with Ig-like domain 3 [Source:HGNC Symbol;Acc:24075]	ILMN_3187328	AMIGO3
adhesion molecule with Ig-like domain 3 [Source:HGNC Symbol;Acc:24075]	ILMN_1700231	AMIGO3
inositol hexakisphosphate kinase 1 [Source:HGNC Symbol;Acc:18360]	ILMN_3187328	IP6K1
inositol hexakisphosphate kinase 1 [Source:HGNC Symbol;Acc:18360]	ILMN_1700231	IP6K1
phosphoinositide-3-kinase, regulatory subunit 1 (alpha) [Source:HGNC Symbol;Acc:8979]	ILMN_1760303	PIK3R1
golgin, RAB6-interacting [Source:HGNC Symbol;Acc:25676]	ILMN_1761911	GORAB
regulator of G-protein signaling 16 [Source:HGNC Symbol;Acc:9997]	ILMN_1808226	RGS16
regulator of G-protein signaling 2, 24kDa [Source:HGNC Symbol;Acc:9998]	ILMN_2197365	RGS2
lysine (K)-specific demethylase 5B [Source:HGNC Symbol;Acc:18039]	ILMN_1755727	KDM5B

microRNA model	l_A	l_B	odds ratio	p-value	Genes
HSA-MIR-410.5	6 (112)	67 (15441)	12.35	0.0077	ADM ETS1 RGS16 CITED2 FAM46A KLHL9
HSA-MIR-323-3P.5	5 (112)	77 (15441)	8.95	0.01	PDE4B SGK1 AUTS2 FAM46A IRF2BPL
HSA-MIR-200B.5	11 (112)	406 (15441)	3.74	0.01	RND3 DUSP1 ETS1 GEM IRS1 NRIP1 KLF4 CITED2 KANK1 ERRFI1 XKR8
HSA-MIR-200C.4	16 (112)	857 (15441)	2.57	0.01	RND3 DUSP1 ETS1 GEM ID2 IRS1 SNAI2 NRIP1 KLF4 CITED2 KANK1 ZC3H4 ERRFI1 XKR8 NFKBIZ FRM
HSA-MIR-200B.4	17 (112)	901 (15441)	2.6	0.01	RND3 KLF9 DUSP1 ETS1 GEM ID2 IRS1 SNAI2 NRIP1 KLF4 CITED2 KANK1 ZC3H4 ERRFI1 XKR8 NFKBIZ
HSA-MIR-200C.5	11 (112)	382 (15441)	3.97	0.01	RND3 DUSP1 ETS1 ID2 IRS1 NRIP1 KLF4 CITED2 KANK1 ERRFI1 XKR8
HSA-MIR-429.4	18 (112)	870 (15441)	2.85	0.01	RND3 KLF9 CEBPD DUSP1 ETS1 GEM ID2 IRS1 SNAI2 NRIP1 KLF4 CITED2 KANK1 ZC3H4 ERRFI1 XKR8
