# Supplementary material for: Transcriptome-Wide Analysis of Messenger RNA Decay in Normal and Osteoarthritic Human Articular Chondrocytes
Source: Arthritis Rheumatol. 2014 Oct 26;66(11):3052–61. doi: 10.1002/art.38849 (PMC4233952; doi:10.1002/art.38849)
Supplement: Supplementary file 3 [file art0066-3052-sd3.pdf]

Tew et al - Transcriptome-wide analysis of mRNA decay in normal and osteoarthritic human articular cartilage

**Supplemental Data 3**

This contain comparisons of mRNA half lives between normal and osteoarthritic chondrocytes. Genes in these comparisons have a >2 fold change in half life and are considered significant if they exhibit a false discovery rate (FDR) < 0.05.

**Column Headings:**
Illumina_ID
Gene ID - official gene symbol
Normal_HL - mean mRNA half life in normal chondrocytes (hours)
Intact_HL - mean mRNA half life in Intact osteoarthritic chondrocytes (hours)
Fibrillated_HL - mean mRNA half life in fibrillated osteoarthritic chondrocytes (hours)
OA_HL - mean mRNA half life in all osteoarthritic chondrocytes (hours)
Normal_HL:OA_HL - Ratio of mean mRNA half life in Normal and all OA datasets
FDR - False Discovery Rate for comparison indicated by tab name

| Illumina_ID | GENE ID | Normal_HL | Intact_HL | Fibrillated_HL | OA_HL | Normal_HL: OA_HL | FDR |
|---|---|---|---|---|---|---|---|
| ILMN_1734611 | RP11-404P21.8 | 14.3 | 2.1 | 2.4 | 2.3 | 6.3 | 4.4E-03 |
| ILMN_1734611 | BDKRB1 | 14.3 | 2.1 | 2.4 | 2.3 | 6.3 | 4.4E-03 |
| ILMN_1670926 | CHST15 | 16.8 | 2.6 | 3.0 | 2.8 | 6.1 | 4.7E-03 |
| ILMN_1692056 | HS3ST3A1 | 18.9 | 3.3 | 3.7 | 3.5 | 5.4 | 2.1E-03 |
| ILMN_1741404 | MSC | 17.6 | 2.8 | 4.2 | 3.5 | 5.0 | 5.7E-03 |
| ILMN_1809364 | NTF3 | 24.0 | 5.0 | 5.8 | 5.4 | 4.5 | 1.3E-09 |
| ILMN_1796471 | GDF5 | 14.8 | 3.0 | 3.7 | 3.3 | 4.4 | 1.1E-02 |
| ILMN_1673566 | ADAMTS1 | 17.6 | 4.0 | 4.1 | 4.0 | 4.4 | 5.1E-03 |
| ILMN_1691846 | G0S2 | 10.0 | 2.3 | 2.5 | 2.4 | 4.2 | 2.2E-02 |
| ILMN_1787691 | CITED4 | 10.4 | 2.6 | 2.4 | 2.5 | 4.2 | 2.1E-02 |
| ILMN_1735045 | A4GALT | 15.4 | 3.6 | 4.0 | 3.8 | 4.1 | 3.7E-03 |
| ILMN_3237270 | MARCH3 | 13.9 | 3.3 | 3.7 | 3.5 | 4.0 | 1.1E-02 |
| ILMN_1792978 | HAS2 | 16.6 | 5.4 | 3.1 | 4.2 | 3.9 | 5.2E-03 |
| ILMN_1707350 | TUSC1 | 10.9 | 2.6 | 3.0 | 2.8 | 3.9 | 1.9E-02 |
| ILMN_1671142 | GPR68 | 18.8 | 4.6 | 5.2 | 4.9 | 3.8 | 3.6E-03 |
| ILMN_1702973 | EVA1A | 23.4 | 8.9 | 3.6 | 6.3 | 3.7 | 3.3E-03 |
| ILMN_1795317 | SCAND1 | 15.5 | 4.5 | 3.9 | 4.2 | 3.7 | 8.0E-03 |
| ILMN_1798459 | PPAN-P2RY11 | 11.1 | 3.3 | 2.7 | 3.0 | 3.7 | 1.8E-02 |
| ILMN_1798459 | PPAN | 11.1 | 3.3 | 2.7 | 3.0 | 3.7 | 1.8E-02 |
| ILMN_1812702 | SIX1 | 9.6 | 2.3 | 3.0 | 2.6 | 3.6 | 2.8E-02 |
| ILMN_1747227 | ADORA1 | 12.7 | 3.3 | 3.8 | 3.5 | 3.6 | 2.0E-02 |
| ILMN_1745271 | EXOSC4 | 14.0 | 4.6 | 3.1 | 3.9 | 3.6 | 1.6E-02 |
| ILMN_1745271 | EXOSC4 | 14.0 | 4.6 | 3.1 | 3.9 | 3.6 | 1.6E-02 |
| ILMN_1819384 | VTRNA1-3 | 12.9 | 3.4 | 3.9 | 3.6 | 3.6 | 1.2E-02 |
| ILMN_1794914 | UBTD1 | 13.3 | 3.2 | 4.3 | 3.7 | 3.6 | 1.9E-02 |
| ILMN_1811258 | RELB | 20.1 | 5.4 | 6.0 | 5.7 | 3.5 | 3.8E-03 |
| ILMN_3239378 | TADA2B | 13.8 | 4.5 | 3.3 | 3.9 | 3.5 | 1.9E-02 |
| ILMN_1754969 | LMCD1 | 9.8 | 2.8 | 2.8 | 2.8 | 3.5 | 2.7E-02 |
| ILMN_1654385 | ASB13 | 9.0 | 2.7 | 2.4 | 2.6 | 3.5 | 2.8E-02 |
| ILMN_2111237 | MN1 | 8.2 | 2.4 | 2.4 | 2.4 | 3.5 | 4.7E-02 |
| ILMN_1671891 | PID1 | 15.5 | 3.4 | 5.5 | 4.5 | 3.5 | 5.2E-03 |
| ILMN_1653001 | CABLES1 | 15.0 | 4.5 | 4.2 | 4.3 | 3.5 | 9.6E-03 |
| ILMN_1699644 | MARCH3 | 17.7 | 4.7 | 5.6 | 5.1 | 3.5 | 6.9E-03 |
| ILMN_1913678 | IRAK3 | 19.1 | 4.4 | 6.7 | 5.5 | 3.5 | 3.6E-03 |
| ILMN_1717706 | PLK2 | 17.0 | 5.1 | 4.9 | 5.0 | 3.4 | 1.2E-02 |
| ILMN_1677402 | C11orf96 | 7.0 | 2.0 | 2.1 | 2.1 | 3.4 | 1.7E-02 |
| ILMN_1714700 | TRIB2 | 11.9 | 3.2 | 3.9 | 3.5 | 3.4 | 1.8E-02 |
| ILMN_1795930 | PTGER4 | 13.7 | 3.7 | 4.4 | 4.1 | 3.4 | 2.0E-02 |
| ILMN_1779428 | C12orf68 | 15.0 | 4.5 | 4.4 | 4.5 | 3.4 | 1.2E-02 |
| ILMN_1760412 | SHISA2 | 16.8 | 4.5 | 5.7 | 5.1 | 3.3 | 3.8E-03 |
| ILMN_1794122 | ZNF79 | 15.3 | 5.3 | 4.0 | 4.7 | 3.3 | 1.4E-02 |
| ILMN_1781001 | SOCS3 | 12.0 | 4.4 | 2.9 | 3.7 | 3.3 | 2.1E-02 |
| ILMN_1726809 | BHLHE41 | 11.3 | 3.2 | 3.7 | 3.4 | 3.3 | 2.3E-02 |
| ILMN_1672121 | C12orf68 | 11.0 | 3.5 | 3.2 | 3.4 | 3.3 | 2.0E-02 |
| ILMN_2166972 | BBS12 | 17.4 | 5.7 | 5.0 | 5.3 | 3.3 | 8.0E-03 |
| ILMN_1747409 | MT1L | 20.0 | 7.0 | 5.4 | 6.2 | 3.2 | 4.7E-03 |
| ILMN_1741628 | ABL2 | 17.2 | 5.4 | 5.3 | 5.3 | 3.2 | 8.8E-03 |
| ILMN_2376723 | CDKN2B | 10.5 | 3.3 | 3.2 | 3.3 | 3.2 | 1.5E-02 |
| ILMN_1652486 | THAP7 | 14.6 | 4.8 | 4.3 | 4.6 | 3.2 | 1.8E-02 |
| ILMN_1796737 | ZBTB7C | 12.0 | 4.1 | 3.5 | 3.8 | 3.2 | 2.1E-02 |
| ILMN_1751171 | IRF2BP1 | 16.2 | 6.6 | 3.6 | 5.1 | 3.2 | 2.0E-02 |
| ILMN_1794501 | HAS3 | 22.8 | 9.4 | 5.1 | 7.2 | 3.2 | 3.5E-03 |
| ILMN_1742782 | GPANK1 | 17.3 | 5.6 | 5.4 | 5.5 | 3.2 | 9.0E-03 |
| ILMN_1752914 | ARHGAP31 | 16.8 | 5.9 | 4.9 | 5.4 | 3.1 | 1.3E-02 |
| ILMN_1771601 | UFSP1 | 16.8 | 6.3 | 4.4 | 5.4 | 3.1 | 1.1E-02 |
| ILMN_3194638 | EVA1A | 24.0 | 9.2 | 6.3 | 7.7 | 3.1 | 2.5E-05 |

| | | | | | | | |
|---|---|---|---|---|---|---|---|
| ILMN_2197128 | OSR1 | 8.9 | 2.7 | 3.0 | 2.9 | 3.1 | 4.3E-02 |
| ILMN_1690839 | PPAPDC3 | 11.9 | 3.7 | 4.0 | 3.8 | 3.1 | 1.8E-02 |
| ILMN_1811468 | IRX3 | 20.0 | 9.0 | 3.9 | 6.5 | 3.1 | 1.2E-02 |
| ILMN_1693004 | SOGA1 | 16.2 | 5.0 | 5.5 | 5.2 | 3.1 | 8.2E-03 |
| ILMN_2232368 | PCIF1 | 14.3 | 4.7 | 4.6 | 4.6 | 3.1 | 1.6E-02 |
| ILMN_2406304 | PDZRN3 | 17.9 | 5.1 | 6.6 | 5.8 | 3.1 | 7.7E-03 |
| ILMN_1765746 | SFT2D3 | 18.3 | 6.7 | 5.2 | 6.0 | 3.1 | 4.5E-03 |
| ILMN_1680856 | MAMLD1 | 18.8 | 6.8 | 5.5 | 6.1 | 3.1 | 4.9E-03 |
| ILMN_1746579 | UBE2O | 10.5 | 3.3 | 3.5 | 3.4 | 3.1 | 2.4E-02 |
| ILMN_1669046 | FOXQ1 | 24.0 | 9.3 | 6.5 | 7.9 | 3.0 | 3.1E-03 |
| ILMN_1657679 | VAV3 | 14.4 | 4.5 | 5.0 | 4.7 | 3.0 | 1.1E-02 |
| ILMN_1682034 | HEY2 | 24.0 | 9.3 | 6.7 | 8.0 | 3.0 | 1.4E-06 |
| ILMN_1801833 | ARHGAP24 | 15.4 | 4.7 | 5.6 | 5.1 | 3.0 | 4.1E-03 |
| ILMN_1672660 | MBP | 20.1 | 8.8 | 4.6 | 6.7 | 3.0 | 1.2E-02 |
| ILMN_1736093 | SNX33 | 8.8 | 3.0 | 2.9 | 2.9 | 3.0 | 4.8E-02 |
| ILMN_1900270 | ETS1 | 13.3 | 4.1 | 4.8 | 4.4 | 3.0 | 2.1E-02 |
| ILMN_1701441 | LPAR1 | 12.6 | 3.6 | 4.8 | 4.2 | 3.0 | 1.7E-02 |
| ILMN_1732197 | MN1 | 9.8 | 3.3 | 3.3 | 3.3 | 3.0 | 3.0E-02 |
| ILMN_1658094 | ZNF365 | 18.9 | 6.5 | 6.2 | 6.4 | 3.0 | 4.7E-03 |
| ILMN_2391891 | MAP2K5 | 18.1 | 6.3 | 5.9 | 6.1 | 2.9 | 6.1E-03 |
| ILMN_1651438 | ZFPM1 | 9.0 | 3.3 | 2.8 | 3.1 | 2.9 | 4.4E-02 |
| ILMN_1913060 | CMKLR1 | 11.8 | 4.1 | 3.9 | 4.0 | 2.9 | 1.6E-02 |
| ILMN_1741021 | CH25H | 24.0 | 4.5 | 11.9 | 8.2 | 2.9 | 4.6E-03 |
| ILMN_1807050 | SHC4 | 18.9 | 5.4 | 7.6 | 6.5 | 2.9 | 1.9E-03 |
| ILMN_2399463 | VAV3 | 16.2 | 8.1 | 3.1 | 5.6 | 2.9 | 3.7E-02 |
| ILMN_1660031 | P2RY6 | 21.5 | 7.6 | 7.2 | 7.4 | 2.9 | 8.7E-04 |
| ILMN_1679217 | FAM110B | 6.2 | 2.1 | 2.1 | 2.1 | 2.9 | 1.8E-02 |
| ILMN_1727553 | C5orf54 | 13.2 | 5.2 | 3.8 | 4.5 | 2.9 | 2.2E-02 |
| ILMN_1677429 | TWIST2 | 19.2 | 7.1 | 6.1 | 6.6 | 2.9 | 3.8E-03 |
| ILMN_3305466 | ZNF788 | 13.9 | 4.8 | 4.8 | 4.8 | 2.9 | 1.6E-02 |
| ILMN_3305466 | ZNF788 | 13.9 | 4.8 | 4.8 | 4.8 | 2.9 | 1.6E-02 |
| ILMN_2373444 | ADORA1 | 18.6 | 5.9 | 7.0 | 6.4 | 2.9 | 4.3E-03 |
| ILMN_1672908 | TWIST1 | 17.0 | 4.7 | 7.1 | 5.9 | 2.9 | 1.4E-02 |
| ILMN_1782922 | PDE4B | 5.8 | 2.0 | 2.0 | 2.0 | 2.9 | 2.1E-02 |
| ILMN_3245707 | RIMKLB | 22.2 | 7.0 | 8.4 | 7.7 | 2.9 | 1.5E-04 |
| ILMN_1651767 | MKL1 | 14.9 | 5.6 | 4.7 | 5.2 | 2.9 | 1.6E-02 |
| ILMN_1677962 | GPHN | 11.1 | 4.3 | 3.5 | 3.9 | 2.9 | 1.1E-02 |
| ILMN_2372915 | P2RY2 | 18.4 | 5.7 | 7.2 | 6.4 | 2.9 | 6.0E-03 |
| ILMN_2402600 | GLIS3 | 10.9 | 3.4 | 4.2 | 3.8 | 2.9 | 2.4E-02 |
| ILMN_1713803 | C17orf97 | 13.5 | 5.7 | 3.8 | 4.7 | 2.9 | 2.4E-02 |
| ILMN_1703955 | FBXO32 | 9.5 | 4.2 | 2.4 | 3.3 | 2.9 | 2.1E-02 |
| ILMN_1719232 | DGCR14 | 15.0 | 6.1 | 4.4 | 5.3 | 2.9 | 1.6E-02 |
| ILMN_2153466 | FAM50B | 8.6 | 2.9 | 3.1 | 3.0 | 2.8 | 2.2E-02 |
| ILMN_1746465 | FJX1 | 13.2 | 5.9 | 3.5 | 4.7 | 2.8 | 2.1E-02 |
| ILMN_1765001 | NAT6 | 14.0 | 4.6 | 5.3 | 5.0 | 2.8 | 1.8E-02 |
| ILMN_1727671 | SSH1 | 20.3 | 7.3 | 7.1 | 7.2 | 2.8 | 2.4E-03 |
| ILMN_1672589 | SEMA4B | 11.9 | 5.1 | 3.3 | 4.2 | 2.8 | 2.0E-02 |
| ILMN_1681886 | ADAMTS5 | 19.7 | 6.3 | 7.7 | 7.0 | 2.8 | 3.0E-03 |
| ILMN_2169736 | PGBD4 | 20.4 | 7.2 | 7.3 | 7.3 | 2.8 | 2.1E-03 |
| ILMN_1690252 | ALKBH2 | 13.9 | 4.9 | 5.0 | 4.9 | 2.8 | 2.0E-02 |
| ILMN_1655796 | MARCH3 | 15.1 | 4.8 | 6.0 | 5.4 | 2.8 | 1.5E-02 |
| ILMN_1805543 | ADAMTS9 | 17.7 | 5.3 | 7.4 | 6.3 | 2.8 | 9.1E-03 |
| ILMN_1677432 | SRGAP1 | 20.9 | 6.7 | 8.4 | 7.6 | 2.8 | 3.6E-03 |
| ILMN_1659537 | MED8 | 14.2 | 5.9 | 4.5 | 5.2 | 2.8 | 2.2E-02 |
| ILMN_1795704 | KIAA0232 | 12.9 | 5.3 | 4.1 | 4.7 | 2.7 | 1.8E-02 |
| ILMN_2226304 | ANKRD50 | 6.8 | 2.5 | 2.4 | 2.5 | 2.7 | 3.5E-02 |
| ILMN_2413572 | MARK2 | 14.8 | 4.8 | 6.0 | 5.4 | 2.7 | 1.6E-02 |
| ILMN_1659106 | PHLDA3 | 14.0 | 4.1 | 6.1 | 5.1 | 2.7 | 2.5E-02 |

| | | | | | | | |
|---|---|---|---|---|---|---|---|
| ILMN_3235312 | MAFG-AS1 | 16.6 | 5.3 | 6.9 | 6.1 | 2.7 | 1.8E-02 |
| ILMN_1683932 | ZNF425 | 15.5 | 6.2 | 5.2 | 5.7 | 2.7 | 7.8E-03 |
| ILMN_1681949 | PDGFRA | 13.1 | 3.8 | 5.9 | 4.8 | 2.7 | 3.1E-02 |
| ILMN_1798926 | SOCS2 | 12.7 | 4.9 | 4.5 | 4.7 | 2.7 | 3.6E-03 |
| ILMN_1728071 | KRAS | 14.1 | 4.8 | 5.6 | 5.2 | 2.7 | 1.9E-02 |
| ILMN_1792951 | ZHX2 | 7.0 | 2.6 | 2.6 | 2.6 | 2.7 | 2.9E-02 |
| ILMN_1849186 | ZNF704 | 20.2 | 6.6 | 8.4 | 7.5 | 2.7 | 5.3E-03 |
| ILMN_1757847 | C11orf68 | 13.9 | 5.3 | 5.1 | 5.2 | 2.7 | 1.8E-02 |
| ILMN_1682081 | RNF19B | 8.8 | 3.4 | 3.1 | 3.3 | 2.7 | 2.8E-02 |
| ILMN_1685109 | POLR3D | 16.1 | 6.9 | 5.0 | 6.0 | 2.7 | 1.1E-02 |
| ILMN_1736628 | AC135048.13 | 15.2 | 6.2 | 5.2 | 5.7 | 2.7 | 1.9E-02 |
| ILMN_1736628 | ORAI3 | 15.2 | 6.2 | 5.2 | 5.7 | 2.7 | 1.9E-02 |
| ILMN_1783606 | KMT2E | 10.5 | 3.9 | 4.0 | 3.9 | 2.7 | 1.8E-02 |
| ILMN_1838942 | MT2A | 10.2 | 3.8 | 3.9 | 3.8 | 2.7 | 3.6E-02 |
| ILMN_1711005 | CDC25A | 18.6 | 9.8 | 4.1 | 6.9 | 2.7 | 1.9E-02 |
| ILMN_3307906 | PALMD | 8.0 | 3.0 | 3.0 | 3.0 | 2.7 | 8.7E-04 |
| ILMN_1701998 | AFAP1 | 16.3 | 6.2 | 6.1 | 6.2 | 2.6 | 4.9E-03 |
| ILMN_2400322 | DYRK3 | 9.7 | 3.8 | 3.6 | 3.7 | 2.6 | 1.4E-02 |
| ILMN_1717799 | PRKCE | 19.4 | 7.1 | 7.7 | 7.4 | 2.6 | 6.3E-03 |
| ILMN_1737650 | DIO2 | 23.1 | 7.2 | 10.4 | 8.8 | 2.6 | 1.7E-03 |
| ILMN_2399304 | NAV2 | 19.2 | 6.1 | 8.5 | 7.3 | 2.6 | 3.7E-03 |
| ILMN_3272768 | LINC00339 | 17.3 | 6.9 | 6.3 | 6.6 | 2.6 | 1.5E-02 |
| ILMN_3251298 | THNSL1 | 12.0 | 4.9 | 4.3 | 4.6 | 2.6 | 2.0E-02 |
| ILMN_1680111 | AHDC1 | 16.4 | 5.5 | 7.1 | 6.3 | 2.6 | 2.0E-02 |
| ILMN_1678494 | ZNF438 | 13.7 | 5.4 | 5.1 | 5.2 | 2.6 | 2.4E-02 |
| ILMN_1757237 | ASAP2 | 19.0 | 7.3 | 7.3 | 7.3 | 2.6 | 7.6E-03 |
| ILMN_1717639 | SIK1 | 19.4 | 10.3 | 4.6 | 7.5 | 2.6 | 1.7E-02 |
| ILMN_1743933 | TSHZ3 | 13.5 | 5.8 | 4.6 | 5.2 | 2.6 | 1.3E-02 |
| ILMN_1813641 | TOR4A | 13.5 | 4.7 | 5.7 | 5.2 | 2.6 | 1.8E-02 |
| ILMN_1734440 | VIPAS39 | 14.9 | 5.5 | 6.0 | 5.8 | 2.6 | 1.6E-02 |
| ILMN_1740200 | USP18 | 15.7 | 6.3 | 5.9 | 6.1 | 2.6 | 1.2E-02 |
| ILMN_2386444 | ANGPTL4 | 10.5 | 3.6 | 4.5 | 4.1 | 2.6 | 1.2E-02 |
| ILMN_1683178 | JAK2 | 8.9 | 3.2 | 3.7 | 3.4 | 2.6 | 3.3E-02 |
| ILMN_1807072 | GOSR2 | 21.1 | 9.3 | 7.1 | 8.2 | 2.6 | 3.5E-03 |
| ILMN_1675756 | KCNJ15 | 23.8 | 9.8 | 8.8 | 9.3 | 2.6 | 3.0E-04 |
| ILMN_1695485 | FAM225A | 20.2 | 6.6 | 9.1 | 7.9 | 2.6 | 5.4E-03 |
| ILMN_1813489 | RAF1 | 15.8 | 6.3 | 6.1 | 6.2 | 2.5 | 1.7E-02 |
| ILMN_1787541 | SPSB2 | 12.3 | 5.3 | 4.4 | 4.8 | 2.5 | 2.2E-02 |
| ILMN_1737005 | SMG9 | 12.1 | 5.1 | 4.4 | 4.8 | 2.5 | 2.2E-02 |
| ILMN_1722891 | CHST10 | 20.6 | 7.6 | 8.6 | 8.1 | 2.5 | 4.6E-03 |
| ILMN_2106902 | FOXN3 | 13.6 | 5.0 | 5.7 | 5.4 | 2.5 | 2.4E-02 |
| ILMN_1764723 | SH3PXD2B | 16.9 | 7.2 | 6.1 | 6.6 | 2.5 | 1.1E-02 |
| ILMN_2390526 | RARB | 22.7 | 9.9 | 8.0 | 8.9 | 2.5 | 9.8E-06 |
| ILMN_1763634 | PEX14 | 11.7 | 4.7 | 4.5 | 4.6 | 2.5 | 2.8E-02 |
| ILMN_1704472 | EID2 | 9.7 | 4.6 | 3.0 | 3.8 | 2.5 | 3.2E-02 |
| ILMN_1774387 | ZHX3 | 18.1 | 7.5 | 6.7 | 7.1 | 2.5 | 5.6E-03 |
| ILMN_1754921 | FAM43B | 12.0 | 4.3 | 5.2 | 4.7 | 2.5 | 1.8E-02 |
| ILMN_1780334 | KCNJ2 | 17.8 | 4.2 | 9.8 | 7.0 | 2.5 | 2.0E-02 |
| ILMN_2367258 | SMOX | 12.3 | 3.7 | 6.0 | 4.9 | 2.5 | 1.8E-02 |
| ILMN_1795218 | MIR1226 | 18.7 | 7.7 | 7.1 | 7.4 | 2.5 | 4.1E-03 |
| ILMN_1795218 | DHX30 | 18.7 | 7.7 | 7.1 | 7.4 | 2.5 | 4.1E-03 |
| ILMN_1720771 | STX11 | 11.4 | 4.9 | 4.1 | 4.5 | 2.5 | 4.0E-02 |
| ILMN_1738816 | FOXO1 | 4.5 | 1.8 | 1.8 | 1.8 | 2.5 | 1.8E-02 |
| ILMN_1718968 | MT1E | 6.6 | 2.7 | 2.6 | 2.6 | 2.5 | 3.0E-02 |
| ILMN_1705066 | BTBD11 | 8.3 | 2.7 | 3.9 | 3.3 | 2.5 | 4.8E-02 |
| ILMN_1668639 | TBC1D10B | 13.7 | 6.1 | 4.8 | 5.5 | 2.5 | 2.8E-02 |
| ILMN_3240901 | ZBTB7C | 15.2 | 7.3 | 4.8 | 6.1 | 2.5 | 3.1E-02 |
| ILMN_2391345 | RPP25L | 18.1 | 7.5 | 7.0 | 7.2 | 2.5 | 8.0E-03 |

| | | | | | | | |
|---|---|---|---|---|---|---|---|
| ILMN_2391345 | DCTN3 | 18.1 | 7.5 | 7.0 | 7.2 | 2.5 | 8.0E-03 |
| ILMN_2211122 | TRAPPC2P1 | 16.1 | 5.3 | 7.6 | 6.5 | 2.5 | 1.0E-02 |
| ILMN_1668411 | FHL2 | 10.0 | 3.8 | 4.2 | 4.0 | 2.5 | 3.8E-02 |
| ILMN_1743367 | FZD4 | 12.7 | 5.2 | 5.0 | 5.1 | 2.5 | 2.0E-02 |
| ILMN_1733045 | RAB36 | 18.7 | 7.3 | 7.7 | 7.5 | 2.5 | 5.1E-03 |
| ILMN_1793522 | PRKAB1 | 13.8 | 6.1 | 5.0 | 5.5 | 2.5 | 1.9E-02 |
| ILMN_1713486 | RNF25 | 16.8 | 8.1 | 5.4 | 6.8 | 2.5 | 9.1E-03 |
| ILMN_2074258 | BARD1 | 23.6 | 11.3 | 7.6 | 9.5 | 2.5 | 3.7E-03 |
| ILMN_1789243 | VPS33B | 11.7 | 5.4 | 4.0 | 4.7 | 2.5 | 2.6E-02 |
| ILMN_2207393 | CNOT3 | 18.5 | 8.6 | 6.3 | 7.4 | 2.5 | 1.6E-02 |
| ILMN_1736828 | CHST10 | 9.1 | 3.8 | 3.6 | 3.7 | 2.5 | 2.4E-02 |
| ILMN_1726704 | DDI2 | 17.0 | 8.7 | 5.1 | 6.9 | 2.5 | 2.0E-02 |
| ILMN_1726704 | RSC1A1 | 17.0 | 8.7 | 5.1 | 6.9 | 2.5 | 2.0E-02 |
| ILMN_3256471 | FAM87A | 20.3 | 8.2 | 8.2 | 8.2 | 2.5 | 8.0E-03 |
| ILMN_3256471 | FAM87B | 20.3 | 8.2 | 8.2 | 8.2 | 2.5 | 8.0E-03 |
| ILMN_1752520 | SLFN11 | 18.7 | 8.1 | 7.0 | 7.6 | 2.5 | 1.5E-02 |
| ILMN_1699249 | ZNF649 | 14.8 | 5.8 | 6.2 | 6.0 | 2.5 | 1.5E-02 |
| ILMN_1773959 | ST6GALNAC5 | 21.8 | 10.0 | 7.6 | 8.8 | 2.5 | 5.1E-03 |
| ILMN_1656335 | RIT1 | 12.3 | 5.4 | 4.6 | 5.0 | 2.5 | 2.1E-02 |
| ILMN_1744239 | FEM1B | 15.1 | 6.9 | 5.3 | 6.1 | 2.5 | 1.8E-02 |
| ILMN_1793474 | INSIG1 | 10.0 | 4.0 | 4.1 | 4.1 | 2.5 | 3.7E-02 |
| ILMN_1701477 | CCDC101 | 17.5 | 7.2 | 7.0 | 7.1 | 2.5 | 1.5E-02 |
| ILMN_1860638 | ST3GAL1 | 6.7 | 2.8 | 2.7 | 2.7 | 2.5 | 1.9E-02 |
| ILMN_2072178 | ECHDC3 | 11.6 | 4.6 | 4.8 | 4.7 | 2.5 | 9.1E-03 |
| ILMN_1801610 | METRNL | 14.1 | 5.5 | 6.0 | 5.8 | 2.5 | 2.2E-02 |
| ILMN_1672295 | ZC3H12A | 10.1 | 4.8 | 3.5 | 4.1 | 2.5 | 2.0E-02 |
| ILMN_1723198 | CDKN2B | 17.4 | 8.1 | 6.1 | 7.1 | 2.5 | 7.2E-03 |
| ILMN_1737360 | TSPY26P | 8.2 | 3.6 | 3.1 | 3.4 | 2.4 | 1.5E-02 |
| ILMN_1741356 | PRICKLE1 | 10.8 | 5.0 | 3.8 | 4.4 | 2.4 | 3.7E-02 |
| ILMN_1746846 | TTLL4 | 13.3 | 4.9 | 5.9 | 5.4 | 2.4 | 2.0E-02 |
| ILMN_1757956 | PCGF1 | 12.1 | 5.4 | 4.5 | 5.0 | 2.4 | 1.9E-02 |
| ILMN_1674376 | ANGPTL4 | 12.9 | 6.2 | 4.3 | 5.3 | 2.4 | 1.3E-02 |
| ILMN_1657898 | SEC14L2 | 16.5 | 8.9 | 4.7 | 6.8 | 2.4 | 1.8E-02 |
| ILMN_1657898 | RP4-539M6.19 | 16.5 | 8.9 | 4.7 | 6.8 | 2.4 | 1.8E-02 |
| ILMN_1657898 | MTFP1 | 16.5 | 8.9 | 4.7 | 6.8 | 2.4 | 1.8E-02 |
| ILMN_1797031 | HSPBAP1 | 15.4 | 7.7 | 4.9 | 6.3 | 2.4 | 1.8E-02 |
| ILMN_1705733 | RPRD2 | 16.4 | 6.3 | 7.2 | 6.7 | 2.4 | 2.1E-02 |
| ILMN_1705733 | RPRD2 | 16.4 | 6.3 | 7.2 | 6.7 | 2.4 | 2.1E-02 |
| ILMN_1705266 | RELA | 15.0 | 5.7 | 6.7 | 6.2 | 2.4 | 1.9E-02 |
| ILMN_1652185 | IL4R | 18.9 | 9.9 | 5.7 | 7.8 | 2.4 | 1.9E-02 |
| ILMN_1744240 | TBC1D31 | 20.1 | 10.6 | 6.0 | 8.3 | 2.4 | 1.5E-02 |
| ILMN_1714741 | RP11-395G23.3 | 19.8 | 9.1 | 7.2 | 8.2 | 2.4 | 3.7E-03 |
| ILMN_2175265 | TMEM11 | 12.4 | 6.4 | 3.8 | 5.1 | 2.4 | 3.7E-02 |
| ILMN_1718129 | MAP2K5 | 15.4 | 7.2 | 5.6 | 6.4 | 2.4 | 1.8E-02 |
| ILMN_2358540 | RBMS1P1 | 14.4 | 4.8 | 7.1 | 5.9 | 2.4 | 8.1E-03 |
| ILMN_2358540 | RBMS1 | 14.4 | 4.8 | 7.1 | 5.9 | 2.4 | 8.1E-03 |
| ILMN_1718907 | TSHZ1 | 9.8 | 4.2 | 3.9 | 4.0 | 2.4 | 2.8E-02 |
| ILMN_1676905 | TIGD7 | 9.3 | 3.9 | 3.9 | 3.9 | 2.4 | 1.4E-02 |
| ILMN_3234997 | MAP7D1 | 10.3 | 3.9 | 4.7 | 4.3 | 2.4 | 3.5E-02 |
| ILMN_1734410 | BNIP1 | 12.4 | 6.1 | 4.2 | 5.2 | 2.4 | 2.6E-02 |
| ILMN_3271098 | EVA1A | 24.0 | 9.2 | 10.8 | 10.0 | 2.4 | 2.3E-03 |
| ILMN_2075603 | MRGPRF | 11.8 | 4.6 | 5.3 | 4.9 | 2.4 | 3.6E-02 |
| ILMN_1797191 | KIAA0040 | 17.9 | 8.3 | 6.6 | 7.5 | 2.4 | 1.2E-02 |
| ILMN_1786310 | MVK | 14.3 | 6.9 | 5.0 | 5.9 | 2.4 | 1.3E-02 |
| ILMN_1776213 | RGMB | 20.1 | 9.7 | 7.1 | 8.4 | 2.4 | 9.1E-03 |
| ILMN_2391750 | SFMBT1 | 14.7 | 7.8 | 4.5 | 6.1 | 2.4 | 2.1E-02 |
| ILMN_1705570 | H2AFY2 | 17.2 | 7.2 | 7.2 | 7.2 | 2.4 | 1.0E-02 |
| ILMN_1748283 | PIM2 | 20.6 | 10.9 | 6.3 | 8.6 | 2.4 | 1.5E-02 |

| | | | | | | | |
|---|---|---|---|---|---|---|---|
| ILMN_1854469 | BICC1 | 15.0 | 5.6 | 6.9 | 6.3 | 2.4 | 1.8E-02 |
| ILMN_2234710 | C12orf60 | 16.4 | 8.7 | 5.1 | 6.9 | 2.4 | 2.4E-02 |
| ILMN_1772910 | GAS1 | 3.6 | 1.4 | 1.6 | 1.5 | 2.4 | 2.8E-02 |
| ILMN_1692896 | JMJD4 | 13.6 | 7.1 | 4.3 | 5.7 | 2.4 | 2.8E-02 |
| ILMN_1882248 | USP51 | 15.2 | 6.4 | 6.3 | 6.4 | 2.4 | 2.0E-02 |
| ILMN_1705442 | CMTM3 | 18.3 | 7.1 | 8.2 | 7.7 | 2.4 | 6.2E-03 |
| ILMN_1665217 | CEP19 | 24.0 | 8.9 | 11.3 | 10.1 | 2.4 | 4.7E-05 |
| ILMN_1666502 | SOBP | 10.6 | 4.6 | 4.3 | 4.4 | 2.4 | 3.5E-02 |
| ILMN_1807372 | SPECC1L-ADORA2A | 20.0 | 7.2 | 9.6 | 8.4 | 2.4 | 2.0E-02 |
| ILMN_1807372 | ADORA2A | 20.0 | 7.2 | 9.6 | 8.4 | 2.4 | 2.0E-02 |
| ILMN_1801124 | MSANTD4 | 4.9 | 2.2 | 1.9 | 2.1 | 2.4 | 2.4E-02 |
| ILMN_1728228 | ZNF548 | 13.9 | 5.3 | 6.4 | 5.8 | 2.4 | 2.2E-02 |
| ILMN_2389347 | NR3C1 | 12.4 | 4.9 | 5.5 | 5.2 | 2.4 | 3.1E-02 |
| ILMN_1807515 | CSTF2T | 14.2 | 6.5 | 5.5 | 6.0 | 2.4 | 2.4E-02 |
| ILMN_3241262 | PABPC4L | 22.3 | 9.4 | 9.3 | 9.4 | 2.4 | 2.4E-03 |
| ILMN_1718831 | TMEM57 | 16.0 | 6.0 | 7.6 | 6.8 | 2.4 | 1.4E-02 |
| ILMN_1660288 | ZNF503-AS2 | 21.7 | 9.0 | 9.4 | 9.2 | 2.4 | 2.1E-03 |
| ILMN_2339655 | MAPK7 | 10.0 | 4.5 | 4.0 | 4.2 | 2.4 | 3.5E-02 |
| ILMN_1684086 | BDKRB2 | 22.1 | 8.5 | 10.2 | 9.4 | 2.4 | 6.1E-03 |
| ILMN_1691702 | ZNF775 | 18.1 | 6.0 | 9.3 | 7.7 | 2.4 | 1.3E-02 |
| ILMN_1703324 | PDSS1 | 20.2 | 10.4 | 6.7 | 8.6 | 2.4 | 1.4E-02 |
| ILMN_1700168 | LARS2 | 17.6 | 9.9 | 5.0 | 7.5 | 2.4 | 2.2E-02 |
| ILMN_1686929 | GPATCH3 | 13.7 | 7.0 | 4.7 | 5.8 | 2.4 | 1.8E-02 |
| ILMN_3245310 | CCDC94 | 15.0 | 7.1 | 5.7 | 6.4 | 2.3 | 2.0E-02 |
| ILMN_1669142 | NAA15 | 19.8 | 8.3 | 8.5 | 8.4 | 2.3 | 1.5E-02 |
| ILMN_2362232 | ZNF331 | 9.1 | 3.9 | 3.9 | 3.9 | 2.3 | 1.3E-02 |
| ILMN_1734276 | PMEPA1 | 21.9 | 8.0 | 10.6 | 9.3 | 2.3 | 2.3E-03 |
| ILMN_2055156 | PAG1 | 22.6 | 12.0 | 7.3 | 9.6 | 2.3 | 4.6E-03 |
| ILMN_1764851 | TP53RK | 11.0 | 6.0 | 3.5 | 4.7 | 2.3 | 3.9E-02 |
| ILMN_3248511 | FAM167A | 24.0 | 9.3 | 11.2 | 10.3 | 2.3 | 3.7E-03 |
| ILMN_1654566 | HSPA1L | 13.3 | 6.2 | 5.2 | 5.7 | 2.3 | 1.1E-02 |
| ILMN_2374244 | DYRK2 | 13.9 | 6.6 | 5.2 | 5.9 | 2.3 | 2.0E-02 |
| ILMN_1657148 | CIRBP-AS1 | 23.3 | 11.3 | 8.6 | 10.0 | 2.3 | 1.6E-04 |
| ILMN_1672834 | SSH2 | 16.1 | 6.6 | 7.1 | 6.9 | 2.3 | 1.5E-02 |
| ILMN_1739840 | LRRC8A | 16.5 | 6.5 | 7.6 | 7.0 | 2.3 | 8.1E-03 |
| ILMN_1811650 | DUS2 | 17.3 | 7.4 | 7.4 | 7.4 | 2.3 | 6.1E-03 |
| ILMN_1681135 | SPATA2 | 16.0 | 6.9 | 6.8 | 6.8 | 2.3 | 1.5E-02 |
| ILMN_1752810 | LARP6 | 4.8 | 2.1 | 2.0 | 2.1 | 2.3 | 3.4E-02 |
| ILMN_1739156 | DOPEY1 | 16.5 | 6.7 | 7.4 | 7.1 | 2.3 | 2.1E-02 |
| ILMN_2212590 | TMEM170A | 17.9 | 10.5 | 4.9 | 7.7 | 2.3 | 2.5E-02 |
| ILMN_1757627 | ZMYND19 | 16.9 | 9.7 | 4.8 | 7.3 | 2.3 | 2.4E-02 |
| ILMN_1770850 | PNMA1 | 16.0 | 5.5 | 8.2 | 6.9 | 2.3 | 1.4E-02 |
| ILMN_1770850 | ELMSAN1 | 16.0 | 5.5 | 8.2 | 6.9 | 2.3 | 1.4E-02 |
| ILMN_1716608 | NGF | 5.0 | 2.1 | 2.2 | 2.1 | 2.3 | 2.4E-02 |
| ILMN_1658834 | ZC3H18 | 18.4 | 7.2 | 8.7 | 7.9 | 2.3 | 4.9E-03 |
| ILMN_1789596 | ETV6 | 12.6 | 5.3 | 5.5 | 5.4 | 2.3 | 2.3E-02 |
| ILMN_1763641 | ZNF614 | 12.3 | 5.1 | 5.5 | 5.3 | 2.3 | 2.7E-02 |
| ILMN_1764770 | OXNAD1 | 14.7 | 7.5 | 5.2 | 6.4 | 2.3 | 2.8E-02 |
| ILMN_1793732 | FARS2 | 12.4 | 5.3 | 5.4 | 5.3 | 2.3 | 1.8E-02 |
| ILMN_1771962 | GLI3 | 12.7 | 5.0 | 6.0 | 5.5 | 2.3 | 2.1E-02 |
| ILMN_1778168 | ELMO2 | 17.9 | 7.6 | 8.0 | 7.8 | 2.3 | 7.7E-03 |
| ILMN_1809708 | KCTD21 | 13.0 | 5.5 | 5.9 | 5.7 | 2.3 | 1.5E-02 |
| ILMN_1677261 | LZTS1 | 11.9 | 5.1 | 5.2 | 5.2 | 2.3 | 3.0E-02 |
| ILMN_1682930 | SIPA1 | 15.9 | 7.2 | 6.6 | 6.9 | 2.3 | 1.4E-02 |
| ILMN_1794230 | SCAND1 | 19.7 | 7.9 | 9.3 | 8.6 | 2.3 | 3.6E-03 |
| ILMN_1669982 | RP11-482H16.1 | 11.7 | 3.7 | 6.5 | 5.1 | 2.3 | 4.9E-02 |
| ILMN_1669982 | CCDC85A | 11.7 | 3.7 | 6.5 | 5.1 | 2.3 | 4.9E-02 |
| ILMN_1805330 | KLHL26 | 17.8 | 8.8 | 6.8 | 7.8 | 2.3 | 1.4E-02 |

| | | | | | | | |
|---|---|---|---|---|---|---|---|
| ILMN_2342066 | METRNL | 14.7 | 5.9 | 6.9 | 6.4 | 2.3 | 2.0E-02 |
| ILMN_1809040 | LDLRAP1 | 15.8 | 6.8 | 6.9 | 6.9 | 2.3 | 1.7E-02 |
| ILMN_2357134 | SPHK1 | 23.4 | 9.5 | 11.0 | 10.2 | 2.3 | 4.6E-03 |
| ILMN_1684197 | GPKOW | 22.0 | 11.0 | 8.3 | 9.6 | 2.3 | 8.6E-03 |
| ILMN_1696684 | FAM90A1 | 22.7 | 9.5 | 10.4 | 9.9 | 2.3 | 3.6E-03 |
| ILMN_2246956 | BCL2 | 11.4 | 4.4 | 5.5 | 5.0 | 2.3 | 3.4E-02 |
| ILMN_1764788 | TNFRSF1B | 4.9 | 2.2 | 2.0 | 2.1 | 2.3 | 2.4E-02 |
| ILMN_1806487 | FAM109B | 10.7 | 4.1 | 5.2 | 4.7 | 2.3 | 1.8E-02 |
| ILMN_3215206 | SMN2 | 22.1 | 11.7 | 7.6 | 9.7 | 2.3 | 6.9E-03 |
| ILMN_3215206 | SMN1 | 22.1 | 11.7 | 7.6 | 9.7 | 2.3 | 6.9E-03 |
| ILMN_1815718 | BTRC | 16.5 | 8.0 | 6.5 | 7.2 | 2.3 | 1.8E-02 |
| ILMN_1740345 | THYN1 | 18.5 | 8.3 | 8.0 | 8.1 | 2.3 | 1.4E-02 |
| ILMN_1807042 | MARCKS | 17.3 | 9.6 | 5.6 | 7.6 | 2.3 | 3.6E-02 |
| ILMN_1790460 | ZNF574 | 17.4 | 8.2 | 7.1 | 7.6 | 2.3 | 1.8E-02 |
| ILMN_1687275 | AJUBA | 15.7 | 9.1 | 4.7 | 6.9 | 2.3 | 4.5E-02 |
| ILMN_1803312 | DIMT1 | 13.3 | 7.0 | 4.7 | 5.8 | 2.3 | 2.8E-02 |
| ILMN_3310840 | MIR21 | 14.9 | 7.9 | 5.2 | 6.6 | 2.3 | 1.5E-02 |
| ILMN_3310840 | VMP1 | 14.9 | 7.9 | 5.2 | 6.6 | 2.3 | 1.5E-02 |
| ILMN_1652008 | KNSTRN | 22.1 | 11.9 | 7.5 | 9.7 | 2.3 | 6.9E-03 |
| ILMN_1665384 | SH3BP5L | 15.1 | 7.1 | 6.2 | 6.7 | 2.3 | 2.0E-02 |
| ILMN_1741585 | SFMBT1 | 18.4 | 10.8 | 5.5 | 8.1 | 2.3 | 2.0E-02 |
| ILMN_1671005 | IRF2BP2 | 7.8 | 3.9 | 3.0 | 3.4 | 2.3 | 2.0E-02 |
| ILMN_1658830 | WBP1L | 5.6 | 2.7 | 2.3 | 2.5 | 2.3 | 1.2E-02 |
| ILMN_1705035 | FBXL7 | 16.7 | 6.8 | 7.9 | 7.4 | 2.3 | 1.8E-02 |
| ILMN_1695108 | RSPO2 | 17.7 | 5.9 | 9.8 | 7.8 | 2.3 | 2.3E-02 |
| ILMN_3245057 | ASAP1 | 18.9 | 6.9 | 9.8 | 8.4 | 2.3 | 3.2E-03 |
| ILMN_1756935 | OSBPL6 | 20.1 | 11.8 | 6.0 | 8.9 | 2.3 | 1.9E-02 |
| ILMN_1784655 | TLCD1 | 19.1 | 8.5 | 8.4 | 8.5 | 2.3 | 9.0E-03 |
| ILMN_1810274 | HOXB2 | 21.3 | 12.7 | 6.1 | 9.4 | 2.3 | 8.0E-03 |
| ILMN_1812297 | CYP26B1 | 10.4 | 4.5 | 4.7 | 4.6 | 2.3 | 5.0E-02 |
| ILMN_1813701 | RASAL2 | 16.8 | 7.0 | 7.9 | 7.5 | 2.2 | 1.5E-02 |
| ILMN_1772821 | KIAA1671 | 17.0 | 9.4 | 5.7 | 7.6 | 2.2 | 3.0E-02 |
| ILMN_1691293 | RNF185 | 14.5 | 5.3 | 7.7 | 6.5 | 2.2 | 2.4E-02 |
| ILMN_1778876 | FAM179B | 14.0 | 6.5 | 6.0 | 6.3 | 2.2 | 1.9E-02 |
| ILMN_1751075 | SETD4 | 14.7 | 7.2 | 5.9 | 6.6 | 2.2 | 1.8E-02 |
| ILMN_1705116 | SLC22A23 | 20.3 | 8.4 | 9.7 | 9.1 | 2.2 | 1.1E-02 |
| ILMN_1718285 | HOXC8 | 13.6 | 5.4 | 6.7 | 6.1 | 2.2 | 2.2E-02 |
| ILMN_1673024 | RBM15B | 15.2 | 9.6 | 4.0 | 6.8 | 2.2 | 4.7E-02 |
| ILMN_1697548 | LPHN2 | 21.0 | 11.3 | 7.4 | 9.4 | 2.2 | 1.1E-02 |
| ILMN_1696048 | MEDAG | 18.8 | 5.5 | 11.4 | 8.4 | 2.2 | 1.8E-02 |
| ILMN_1729455 | EML1 | 24.0 | 7.3 | 14.3 | 10.8 | 2.2 | 5.1E-03 |
| ILMN_1776674 | SAC3D1 | 11.5 | 5.6 | 4.7 | 5.2 | 2.2 | 4.0E-02 |
| ILMN_1736112 | ARHGAP10 | 10.3 | 4.8 | 4.5 | 4.6 | 2.2 | 4.7E-02 |
| ILMN_1756590 | SYS1 | 15.1 | 8.2 | 5.3 | 6.8 | 2.2 | 3.0E-02 |
| ILMN_1801441 | RFTN2 | 15.5 | 5.6 | 8.3 | 7.0 | 2.2 | 1.9E-02 |
| ILMN_2364535 | CTD-2323K18.1 | 11.3 | 5.1 | 5.1 | 5.1 | 2.2 | 4.7E-02 |
| ILMN_2364535 | SNUPN | 11.3 | 5.1 | 5.1 | 5.1 | 2.2 | 4.7E-02 |
| ILMN_3245410 | LINC00638 | 20.1 | 7.8 | 10.2 | 9.0 | 2.2 | 9.8E-03 |
| ILMN_1695945 | MEIS2 | 9.2 | 5.2 | 3.1 | 4.1 | 2.2 | 3.4E-02 |
| ILMN_1714170 | SPSB1 | 4.8 | 2.2 | 2.1 | 2.1 | 2.2 | 3.7E-02 |
| ILMN_1739496 | PRRX1 | 9.6 | 3.6 | 5.0 | 4.3 | 2.2 | 2.4E-02 |
| ILMN_2103388 | ZBTB49 | 16.5 | 8.0 | 6.8 | 7.4 | 2.2 | 1.7E-02 |
| ILMN_1785644 | AP5S1 | 14.1 | 6.3 | 6.5 | 6.4 | 2.2 | 3.2E-02 |
| ILMN_1785177 | DNAJC14 | 10.5 | 5.2 | 4.3 | 4.7 | 2.2 | 4.0E-02 |
| ILMN_1729987 | SRC | 9.8 | 5.1 | 3.8 | 4.4 | 2.2 | 4.8E-02 |
| ILMN_1694923 | PTPN9 | 17.4 | 7.2 | 8.5 | 7.9 | 2.2 | 1.7E-02 |
| ILMN_1799814 | SNRNP40 | 20.5 | 11.5 | 7.1 | 9.3 | 2.2 | 1.2E-02 |
| ILMN_1805590 | NAA38 | 19.9 | 11.0 | 7.0 | 9.0 | 2.2 | 1.8E-02 |

| | | | | | | | |
|---|---|---|---|---|---|---|---|
| ILMN_1904628 | S100A6 | 15.5 | 8.7 | 5.3 | 7.0 | 2.2 | 3.5E-02 |
| ILMN_1686454 | TIFA | 16.9 | 10.5 | 4.8 | 7.7 | 2.2 | 3.6E-02 |
| ILMN_1863185 | RP1-39G22.7 | 12.3 | 6.7 | 4.5 | 5.6 | 2.2 | 4.4E-02 |
| ILMN_3250446 | ZNF626 | 19.6 | 10.0 | 7.8 | 8.9 | 2.2 | 9.2E-03 |
| ILMN_1751051 | C7orf25 | 9.6 | 5.0 | 3.7 | 4.4 | 2.2 | 2.7E-02 |
| ILMN_1751051 | PSMA2 | 9.6 | 5.0 | 3.7 | 4.4 | 2.2 | 2.7E-02 |
| ILMN_1773927 | C12orf61 | 17.8 | 7.4 | 8.8 | 8.1 | 2.2 | 1.6E-02 |
| ILMN_2131493 | MAVS | 16.0 | 8.3 | 6.2 | 7.3 | 2.2 | 2.0E-02 |
| ILMN_2080611 | PDSS1 | 18.6 | 11.4 | 5.6 | 8.5 | 2.2 | 2.2E-02 |
| ILMN_1815130 | MICALL1 | 21.1 | 9.8 | 9.5 | 9.6 | 2.2 | 7.7E-03 |
| ILMN_1809566 | ZSCAN16 | 15.9 | 9.8 | 4.8 | 7.3 | 2.2 | 4.2E-02 |
| ILMN_2179083 | LOXL4 | 19.1 | 7.2 | 10.3 | 8.7 | 2.2 | 9.3E-03 |
| ILMN_1776181 | BIRC3 | 5.8 | 2.6 | 2.7 | 2.6 | 2.2 | 3.1E-02 |
| ILMN_1727574 | ZNF827 | 13.3 | 6.0 | 6.1 | 6.1 | 2.2 | 7.0E-03 |
| ILMN_1806946 | UBTF | 19.0 | 8.1 | 9.3 | 8.7 | 2.2 | 9.1E-03 |
| ILMN_1838313 | LRRC8B | 24.0 | 13.0 | 9.0 | 11.0 | 2.2 | 3.6E-03 |
| ILMN_1666004 | WASL | 18.7 | 10.3 | 6.9 | 8.6 | 2.2 | 2.0E-02 |
| ILMN_1743034 | KIF1B | 16.3 | 9.7 | 5.2 | 7.5 | 2.2 | 3.0E-02 |
| ILMN_1743034 | RN7SL731P | 16.3 | 9.7 | 5.2 | 7.5 | 2.2 | 3.0E-02 |
| ILMN_1829989 | ZEB1-AS1 | 13.4 | 5.8 | 6.5 | 6.2 | 2.2 | 2.0E-02 |
| ILMN_1660847 | PFKFB3 | 13.5 | 6.8 | 5.5 | 6.2 | 2.2 | 3.7E-02 |
| ILMN_1681780 | MKX | 18.3 | 10.6 | 6.1 | 8.4 | 2.2 | 2.4E-02 |
| ILMN_2157951 | STX6 | 17.1 | 9.9 | 5.7 | 7.8 | 2.2 | 3.9E-02 |
| ILMN_1763328 | GZF1 | 19.1 | 11.5 | 6.0 | 8.7 | 2.2 | 1.9E-02 |
| ILMN_1737254 | USP1 | 15.2 | 9.5 | 4.5 | 7.0 | 2.2 | 4.9E-02 |
| ILMN_1687213 | FAM167A | 22.1 | 9.7 | 10.7 | 10.2 | 2.2 | 2.1E-03 |
| ILMN_1781906 | RBM17 | 13.6 | 7.6 | 4.9 | 6.3 | 2.2 | 3.7E-02 |
| ILMN_1695290 | FERMT2 | 12.0 | 4.5 | 6.6 | 5.5 | 2.2 | 2.6E-02 |
| ILMN_1664034 | ZNF485 | 18.8 | 11.5 | 5.8 | 8.7 | 2.2 | 1.6E-02 |
| ILMN_1655608 | KLHL18 | 15.1 | 9.4 | 4.6 | 7.0 | 2.2 | 4.9E-02 |
| ILMN_1741976 | SMARCAD1 | 8.8 | 5.1 | 3.0 | 4.0 | 2.2 | 4.7E-02 |
| ILMN_1679655 | WDR82 | 9.1 | 3.9 | 4.6 | 4.2 | 2.2 | 1.5E-02 |
| ILMN_2199298 | TPGS1 | 18.9 | 8.5 | 8.9 | 8.7 | 2.2 | 7.7E-03 |
| ILMN_1687743 | BTBD7 | 10.1 | 5.1 | 4.3 | 4.7 | 2.2 | 2.8E-02 |
| ILMN_2186137 | RRAD | 19.3 | 6.6 | 11.3 | 9.0 | 2.2 | 2.5E-02 |
| ILMN_3178406 | KLHL29 | 17.5 | 6.5 | 9.8 | 8.1 | 2.2 | 1.8E-02 |
| ILMN_1806845 | ALG3 | 20.4 | 12.0 | 6.9 | 9.5 | 2.2 | 1.5E-02 |
| ILMN_1718712 | FAM217B | 20.5 | 12.8 | 6.2 | 9.5 | 2.2 | 1.4E-02 |
| ILMN_1708203 | OTUD4 | 17.0 | 10.1 | 5.7 | 7.9 | 2.1 | 2.2E-02 |
| ILMN_1661653 | DET1 | 17.9 | 6.6 | 10.1 | 8.3 | 2.1 | 1.6E-02 |
| ILMN_2376520 | PPP2R3A | 15.9 | 7.0 | 7.9 | 7.4 | 2.1 | 1.3E-02 |
| ILMN_1718770 | MAMSTR | 20.9 | 9.5 | 10.1 | 9.8 | 2.1 | 2.2E-03 |
| ILMN_1733863 | UBALD1 | 14.7 | 7.9 | 5.9 | 6.9 | 2.1 | 3.6E-02 |
| ILMN_1673370 | FBXL5 | 17.5 | 9.2 | 7.2 | 8.2 | 2.1 | 1.8E-02 |
| ILMN_1758086 | SNAI1 | 16.7 | 7.3 | 8.4 | 7.8 | 2.1 | 1.2E-02 |
| ILMN_1690546 | PPP3CC | 8.2 | 4.1 | 3.6 | 3.9 | 2.1 | 4.9E-02 |
| ILMN_1663976 | PDLIM4 | 20.8 | 6.9 | 12.6 | 9.7 | 2.1 | 1.3E-02 |
| ILMN_1691487 | TRAF2 | 16.4 | 7.9 | 7.5 | 7.7 | 2.1 | 1.7E-02 |
| ILMN_1758250 | TRAFD1 | 11.6 | 5.1 | 5.7 | 5.4 | 2.1 | 3.5E-03 |
| ILMN_3238740 | CENPBD1 | 8.4 | 4.4 | 3.4 | 3.9 | 2.1 | 3.4E-02 |
| ILMN_3187254 | RP11-209M4.1 | 16.9 | 10.3 | 5.6 | 7.9 | 2.1 | 4.0E-02 |
| ILMN_1695961 | CLK3 | 18.1 | 11.6 | 5.5 | 8.5 | 2.1 | 2.7E-02 |
| ILMN_1704842 | ARL4A | 23.3 | 14.2 | 7.7 | 10.9 | 2.1 | 4.4E-03 |
| ILMN_1726368 | ZNF135 | 18.0 | 8.8 | 8.1 | 8.5 | 2.1 | 2.1E-02 |
| ILMN_1741985 | BBS10 | 13.7 | 7.0 | 5.9 | 6.4 | 2.1 | 1.6E-02 |
| ILMN_1695246 | KLHDC8B | 16.6 | 8.0 | 7.6 | 7.8 | 2.1 | 3.9E-03 |
| ILMN_1693702 | MRFAP1L1 | 18.1 | 7.6 | 9.4 | 8.5 | 2.1 | 9.2E-03 |
| ILMN_1705433 | CBLL1 | 15.6 | 6.9 | 7.8 | 7.4 | 2.1 | 1.9E-02 |

| | | | | | | | |
|---|---|---|---|---|---|---|---|
| ILMN_1743275 | SH3RF3 | 20.5 | 9.5 | 9.8 | 9.7 | 2.1 | 1.1E-02 |
| ILMN_1777397 | MSX1 | 15.3 | 9.8 | 4.6 | 7.2 | 2.1 | 4.5E-02 |
| ILMN_2342695 | PDGFA | 18.8 | 6.9 | 10.9 | 8.9 | 2.1 | 2.9E-02 |
| ILMN_1789775 | WDR74 | 10.6 | 6.1 | 3.9 | 5.0 | 2.1 | 4.6E-02 |
| ILMN_1752283 | ITCH | 13.6 | 6.5 | 6.4 | 6.4 | 2.1 | 3.7E-02 |
| ILMN_1801121 | SENP2 | 19.4 | 11.2 | 7.2 | 9.2 | 2.1 | 1.8E-02 |
| ILMN_2201966 | N4BP1 | 12.8 | 5.8 | 6.3 | 6.1 | 2.1 | 2.0E-02 |
| ILMN_1705201 | FOXC2 | 14.1 | 7.2 | 6.2 | 6.7 | 2.1 | 3.4E-02 |
| ILMN_2353240 | USF1 | 23.5 | 11.7 | 10.6 | 11.2 | 2.1 | 7.8E-03 |
| ILMN_1754121 | CSK | 20.5 | 8.6 | 10.8 | 9.7 | 2.1 | 5.1E-03 |
| ILMN_1746276 | EPC1 | 15.7 | 9.2 | 5.7 | 7.5 | 2.1 | 3.3E-02 |
| ILMN_1663772 | SIX2 | 16.5 | 6.5 | 9.2 | 7.9 | 2.1 | 2.3E-02 |
| ILMN_1798256 | UPP1 | 19.8 | 9.5 | 9.3 | 9.4 | 2.1 | 5.6E-03 |
| ILMN_3257145 | ARHGAP42 | 20.9 | 10.5 | 9.4 | 9.9 | 2.1 | 1.1E-02 |
| ILMN_1651692 | STK10 | 18.8 | 10.8 | 7.0 | 8.9 | 2.1 | 1.5E-02 |
| ILMN_1849228 | LINC01096 | 15.3 | 6.9 | 7.6 | 7.3 | 2.1 | 3.1E-02 |
| ILMN_1815759 | CTDP1 | 17.5 | 11.1 | 5.5 | 8.3 | 2.1 | 3.6E-02 |
| ILMN_1882590 | NACC2 | 13.4 | 7.4 | 5.4 | 6.4 | 2.1 | 2.1E-02 |
| ILMN_2200915 | RIPPLY2 | 21.6 | 8.1 | 12.5 | 10.3 | 2.1 | 9.3E-03 |
| ILMN_1813423 | NAA60 | 13.5 | 6.9 | 5.9 | 6.4 | 2.1 | 3.6E-02 |
| ILMN_1813423 | NAA60 | 13.5 | 6.9 | 5.9 | 6.4 | 2.1 | 3.6E-02 |
| ILMN_1857897 | AGAP1 | 20.4 | 10.6 | 8.9 | 9.7 | 2.1 | 1.3E-02 |
| ILMN_1797046 | MTHFSD | 17.7 | 8.0 | 8.9 | 8.4 | 2.1 | 2.0E-02 |
| ILMN_1759232 | IRS1 | 4.1 | 2.2 | 1.8 | 2.0 | 2.1 | 4.1E-02 |
| ILMN_2100693 | MAP2K4 | 16.2 | 10.1 | 5.3 | 7.7 | 2.1 | 4.2E-02 |
| ILMN_2319952 | VDR | 17.1 | 10.6 | 5.7 | 8.2 | 2.1 | 4.2E-02 |
| ILMN_3239060 | KRBA1 | 13.8 | 6.8 | 6.4 | 6.6 | 2.1 | 2.0E-02 |
| ILMN_1692219 | RAB11FIP1 | 16.9 | 10.0 | 6.2 | 8.1 | 2.1 | 4.0E-02 |
| ILMN_1809245 | PITPNB | 10.9 | 5.6 | 4.9 | 5.2 | 2.1 | 3.9E-02 |
| ILMN_1709044 | TGIF2 | 17.2 | 10.4 | 6.1 | 8.2 | 2.1 | 4.0E-02 |
| ILMN_3307719 | ZNF490 | 17.6 | 10.5 | 6.3 | 8.4 | 2.1 | 3.6E-02 |
| ILMN_1688725 | UTP14A | 18.6 | 11.4 | 6.5 | 8.9 | 2.1 | 2.1E-02 |
| ILMN_1798817 | SDR42E1 | 16.4 | 7.8 | 7.9 | 7.9 | 2.1 | 1.4E-02 |
| ILMN_1751773 | POLD3 | 17.1 | 10.2 | 6.2 | 8.2 | 2.1 | 2.8E-02 |
| ILMN_1742923 | ZNF584 | 19.3 | 7.6 | 10.9 | 9.3 | 2.1 | 1.1E-02 |
| ILMN_3272603 | FAM60A | 20.4 | 11.2 | 8.4 | 9.8 | 2.1 | 2.0E-02 |
| ILMN_1703511 | PDZRN3 | 12.7 | 5.2 | 7.1 | 6.1 | 2.1 | 3.3E-02 |
| ILMN_1653730 | OXCT2P1 | 14.7 | 6.8 | 7.3 | 7.0 | 2.1 | 2.4E-02 |
| ILMN_1653730 | OXCT2 | 14.7 | 6.8 | 7.3 | 7.0 | 2.1 | 2.4E-02 |
| ILMN_1765500 | NDUFV3 | 12.1 | 5.9 | 5.7 | 5.8 | 2.1 | 3.4E-02 |
| ILMN_2354269 | ZC2HC1C | 17.7 | 10.9 | 6.2 | 8.5 | 2.1 | 3.3E-02 |
| ILMN_1805192 | ITPRIP | 4.1 | 2.1 | 1.8 | 2.0 | 2.1 | 2.4E-02 |
| ILMN_3237956 | ZC3H12C | 20.3 | 11.5 | 8.0 | 9.8 | 2.1 | 1.2E-02 |
| ILMN_1678075 | CDYL | 16.2 | 8.2 | 7.4 | 7.8 | 2.1 | 2.3E-02 |
| ILMN_1702763 | ZMYM1 | 20.0 | 11.3 | 8.0 | 9.7 | 2.1 | 1.4E-02 |
| ILMN_1720926 | PSMD5 | 20.1 | 13.0 | 6.5 | 9.7 | 2.1 | 1.8E-02 |
| ILMN_2323944 | FAM110A | 17.9 | 11.1 | 6.3 | 8.7 | 2.1 | 2.4E-02 |
| ILMN_1695847 | ZKSCAN5 | 16.0 | 8.9 | 6.6 | 7.7 | 2.1 | 1.4E-02 |
| ILMN_1695357 | SPDL1 | 19.9 | 11.4 | 7.9 | 9.6 | 2.1 | 1.7E-02 |
| ILMN_3235647 | SIK1 | 18.6 | 12.3 | 5.7 | 9.0 | 2.1 | 2.4E-02 |
| ILMN_1672135 | ZNF615 | 8.3 | 3.9 | 4.1 | 4.0 | 2.1 | 2.0E-02 |
| ILMN_1793543 | C1orf51 | 8.2 | 3.8 | 4.2 | 4.0 | 2.1 | 3.3E-02 |
| ILMN_3244176 | RP11-166D19.1 | 14.3 | 5.2 | 8.7 | 7.0 | 2.1 | 3.8E-02 |
| ILMN_1702691 | TNFAIP3 | 16.7 | 10.4 | 5.9 | 8.1 | 2.1 | 3.8E-02 |
| ILMN_1676010 | SP1 | 11.1 | 6.3 | 4.6 | 5.4 | 2.1 | 4.2E-02 |
| ILMN_2182647 | PINX1 | 21.1 | 11.2 | 9.4 | 10.3 | 2.1 | 1.3E-02 |
| ILMN_1755850 | ZNF350 | 12.4 | 6.3 | 5.9 | 6.1 | 2.0 | 3.1E-02 |
| ILMN_1742738 | LURAP1 | 16.4 | 8.8 | 7.2 | 8.0 | 2.0 | 2.0E-02 |

| | | | | | | | |
|---|---|---|---|---|---|---|---|
| ILMN_2205032 | MAGEE1 | 14.5 | 6.4 | 7.8 | 7.1 | 2.0 | 3.3E-02 |
| ILMN_2317730 | ELMO2 | 12.0 | 5.2 | 6.6 | 5.9 | 2.0 | 4.6E-02 |
| ILMN_2250923 | FOXP1 | 18.9 | 10.6 | 7.8 | 9.2 | 2.0 | 1.8E-02 |
| ILMN_2413041 | TEAD4 | 17.7 | 9.3 | 8.0 | 8.7 | 2.0 | 1.8E-02 |
| ILMN_1679891 | NAF1 | 20.5 | 12.1 | 8.0 | 10.0 | 2.0 | 1.4E-02 |
| ILMN_1682139 | RAI14 | 21.8 | 8.9 | 12.4 | 10.7 | 2.0 | 5.2E-03 |
| ILMN_1791002 | SKP2 | 20.7 | 13.9 | 6.3 | 10.1 | 2.0 | 1.8E-02 |
| ILMN_1793578 | ZFP37 | 6.8 | 3.3 | 3.3 | 3.3 | 2.0 | 1.8E-02 |
| ILMN_1791057 | IFNAR2 | 21.1 | 9.4 | 11.3 | 10.3 | 2.0 | 9.2E-03 |
| ILMN_1740490 | ZFP82 | 8.9 | 4.1 | 4.6 | 4.3 | 2.0 | 4.0E-02 |
| ILMN_2230577 | KCTD7 | 13.2 | 6.6 | 6.3 | 6.5 | 2.0 | 3.5E-02 |
| ILMN_2230577 | RABGEF1 | 13.2 | 6.6 | 6.3 | 6.5 | 2.0 | 3.5E-02 |
| ILMN_1677785 | ZNF559 | 13.8 | 7.1 | 6.4 | 6.8 | 2.0 | 1.4E-02 |
| ILMN_1783728 | TBRG4 | 11.0 | 6.2 | 4.6 | 5.4 | 2.0 | 3.7E-02 |
| ILMN_1686968 | AL513327.1 | 12.2 | 5.1 | 6.8 | 6.0 | 2.0 | 2.4E-02 |
| ILMN_1686968 | ZNF362 | 12.2 | 5.1 | 6.8 | 6.0 | 2.0 | 2.4E-02 |
| ILMN_1703926 | PTGER2 | 12.3 | 5.3 | 6.8 | 6.0 | 2.0 | 2.0E-02 |
| ILMN_1811972 | MYCBP2 | 17.0 | 7.4 | 9.4 | 8.4 | 2.0 | 1.8E-02 |
| ILMN_1718900 | KCTD2 | 22.8 | 10.0 | 12.5 | 11.2 | 2.0 | 2.2E-03 |
| ILMN_2176931 | PELI3 | 19.8 | 10.3 | 9.2 | 9.8 | 2.0 | 7.1E-03 |
| ILMN_1683082 | RPUSD1 | 15.8 | 10.0 | 5.6 | 7.8 | 2.0 | 4.5E-02 |
| ILMN_1753482 | FAM86C1 | 17.1 | 6.3 | 10.6 | 8.4 | 2.0 | 2.0E-02 |
| ILMN_2119535 | RIPK1 | 9.9 | 5.4 | 4.3 | 4.9 | 2.0 | 3.0E-02 |
| ILMN_1726153 | MIR4435-1HG | 14.7 | 9.7 | 4.9 | 7.3 | 2.0 | 4.1E-02 |
| ILMN_2369603 | CPEB1 | 22.2 | 10.8 | 11.2 | 11.0 | 2.0 | 3.5E-03 |
| ILMN_2370208 | CMTM3 | 22.0 | 8.1 | 13.6 | 10.9 | 2.0 | 5.6E-03 |
| ILMN_1704637 | TRMT44 | 22.1 | 13.5 | 8.3 | 10.9 | 2.0 | 3.4E-03 |
| ILMN_1781198 | PPP1R3D | 11.0 | 5.9 | 5.0 | 5.4 | 2.0 | 2.1E-02 |
| ILMN_1661695 | IRAK3 | 15.8 | 5.5 | 10.2 | 7.8 | 2.0 | 1.5E-02 |
| ILMN_1677404 | RAP2A | 21.5 | 11.6 | 9.8 | 10.7 | 2.0 | 1.6E-02 |
| ILMN_1654357 | ZNF24 | 18.0 | 11.9 | 5.9 | 8.9 | 2.0 | 3.1E-02 |
| ILMN_1808047 | PHC2 | 8.2 | 4.5 | 3.6 | 4.1 | 2.0 | 2.3E-02 |
| ILMN_1725312 | RASA1 | 13.0 | 5.7 | 7.2 | 6.5 | 2.0 | 1.8E-02 |
| ILMN_1703487 | LMO4 | 20.7 | 8.1 | 12.5 | 10.3 | 2.0 | 9.1E-03 |
| ILMN_2415421 | SLC30A2 | 16.3 | 6.8 | 9.4 | 8.1 | 2.0 | 4.2E-02 |
| ILMN_1752510 | FAM13A | 14.8 | 7.8 | 6.9 | 7.4 | 2.0 | 3.7E-02 |
| ILMN_2283196 | ZSWIM3 | 18.0 | 11.3 | 6.6 | 9.0 | 2.0 | 1.9E-02 |
| ILMN_1719064 | KCTD10 | 11.6 | 6.8 | 4.7 | 5.7 | 2.0 | 4.5E-02 |
| ILMN_1698038 | INMT-FAM188B | 24.0 | 9.0 | 14.9 | 11.9 | 2.0 | 3.6E-03 |
| ILMN_1698038 | FAM188B | 24.0 | 9.0 | 14.9 | 11.9 | 2.0 | 3.6E-03 |
| ILMN_2341254 | STARD13 | 4.4 | 2.1 | 2.3 | 2.2 | 2.0 | 2.3E-02 |
| ILMN_1749424 | SSR4P1 | 15.8 | 8.8 | 7.0 | 7.9 | 2.0 | 2.5E-02 |
| ILMN_1730794 | SERTAD4 | 21.8 | 7.4 | 14.3 | 10.8 | 2.0 | 1.3E-02 |
| ILMN_1679929 | KLF13 | 5.4 | 2.7 | 2.7 | 2.7 | 2.0 | 2.2E-02 |
| ILMN_1690241 | BATF2 | 21.4 | 11.5 | 9.7 | 10.6 | 2.0 | 3.7E-03 |
| ILMN_2414848 | TBRG4 | 12.1 | 7.4 | 4.7 | 6.0 | 2.0 | 2.0E-02 |
| ILMN_2197247 | POLR3A | 11.2 | 6.3 | 4.9 | 5.6 | 2.0 | 4.2E-02 |
| ILMN_1719202 | ZNF174 | 17.1 | 10.3 | 6.8 | 8.5 | 2.0 | 4.0E-02 |
| ILMN_3308118 | RP11-166D19.1 | 15.4 | 7.0 | 8.3 | 7.7 | 2.0 | 2.5E-02 |
| ILMN_3308118 | MIR125B1 | 15.4 | 7.0 | 8.3 | 7.7 | 2.0 | 2.5E-02 |
| ILMN_1783910 | TRAF6 | 15.9 | 8.9 | 6.9 | 7.9 | 2.0 | 3.6E-02 |
| ILMN_2314007 | TCF12 | 19.7 | 10.7 | 9.0 | 9.8 | 2.0 | 1.8E-02 |
| ILMN_1656373 | BNC2 | 7.4 | 3.6 | 3.7 | 3.7 | 2.0 | 1.4E-02 |
| ILMN_1692145 | ZNF14 | 6.7 | 12.0 | 14.9 | 13.4 | 0.5 | 4.6E-02 |
| ILMN_1689552 | FAM63A | 11.1 | 20.6 | 24.0 | 22.3 | 0.5 | 1.3E-02 |
| ILMN_2399310 | MLLT10 | 9.5 | 17.7 | 22.5 | 20.1 | 0.5 | 1.5E-02 |
| ILMN_2371590 | DDX17 | 10.1 | 19.5 | 24.0 | 21.7 | 0.5 | 1.8E-02 |
| ILMN_3243664 | NPIPB11 | 8.2 | 14.2 | 22.6 | 18.4 | 0.4 | 2.0E-02 |

| | | | | | | | |
|---|---|---|---|---|---|---|---|
| ILMN_2196347 | CDKN1B | 6.5 | 16.8 | 13.5 | 15.1 | 0.4 | 1.7E-02 |
| ILMN_2412380 | TSC22D1 | 3.9 | 11.5 | 10.8 | 11.1 | 0.4 | 3.7E-02 |
| ILMN_1680279 | USP49 | 7.0 | 19.8 | 21.9 | 20.8 | 0.3 | 2.1E-03 |
